# The 24-hour molecular landscape after exercise in humans reveals *MYC* is sufficient for muscle growth

**DOI:** 10.1038/s44319-024-00299-z

**Published:** 2024-10-31

**Authors:** Sebastian Edman, Ronald G Jones III, Paulo R Jannig, Rodrigo Fernandez-Gonzalo, Jessica Norrbom, Nicholas T Thomas, Sabin Khadgi, Pieter J Koopmans, Francielly Morena, Toby L Chambers, Calvin S Peterson, Logan N Scott, Nicholas P Greene, Vandre C Figueiredo, Christopher S Fry, Liu Zhengye, Johanna T Lanner, Yuan Wen, Björn Alkner, Kevin A Murach, Ferdinand von Walden

**Affiliations:** 1 Division of Pediatric Neurology, Department of Women’s and Children’s Health, Karolinska Institute, Stockholm, Sweden; 2 Exercise Science Research Center, Department of Health, Human Performance and Recreation, University of Arkansas, Fayetteville, AR USA; 3 Division of Clinical Physiology, Department of Laboratory Medicine, Karolinska Institute, Stockholm, Sweden; 4 Unit of Clinical Physiology, Karolinska University Hospital, Huddinge, Sweden; 5 Molecular Exercise Physiology Group, Department of Physiology and Pharmacology, Karolinska Institute, Stockholm, Sweden; 6 Center for Muscle Biology, University of Kentucky, Lexington, KY USA; 7 Department of Athletic Training and Clinical Nutrition, University of Kentucky, Lexington, KY USA; 8 Cell and Molecular Biology Graduate Program, University of Arkansas, Fayetteville, AR USA; 9 Department of Physiology, University of Kentucky, Lexington, KY USA; 10 Division of Biomedical Informatics, Department of Internal Medicine, University of Kentucky, Lexington, KY USA; 11 Department of Biological Sciences, Oakland University, Rochester Hills, MI USA; 12 Molecular Muscle Physiology & Pathophysiology Group, Department of Physiology & Pharmacology, Karolinska Institute, Stockholm, Sweden; 13 Department of Orthopaedic Surgery, Region Jönköping County, Eksjö, Sweden; 14 Department of Biomedical and Clinical Sciences, Linköping University, Linköping, Sweden

**Keywords:** Time Course, Biopsy, Transcriptome, Methylome, Transcription Factors, Methods & Resources, Musculoskeletal System

## Abstract

A detailed understanding of molecular responses to a hypertrophic stimulus in skeletal muscle leads to therapeutic advances aimed at promoting muscle mass. To decode the molecular factors regulating skeletal muscle mass, we utilized a 24-h time course of human muscle biopsies after a bout of resistance exercise. Our findings indicate: (1) the DNA methylome response at 30 min corresponds to upregulated genes at 3 h, (2) a burst of translation- and transcription-initiation factor-coding transcripts occurs between 3 and 8 h, (3) changes to global protein-coding gene expression peaks at 8 h, (4) ribosome-related genes dominate the mRNA landscape between 8 and 24 h, (5) methylation-regulated *MYC* is a highly influential transcription factor throughout recovery. To test whether MYC is sufficient for hypertrophy, we periodically pulse MYC in skeletal muscle over 4 weeks. Transient MYC increases muscle mass and fiber size in the soleus of adult mice. We present a temporally resolved resource for understanding molecular adaptations to resistance exercise in muscle (http://data.myoanalytics.com) and suggest that controlled MYC doses influence the exercise-related hypertrophic transcriptional landscape.

## Synopsis

A 24-h time course of recovery after an acute hypertrophic stimulus—resistance exercise—unravels the temporal dynamics of gene expression in healthy untrained human skeletal muscle. *MYC*’s pulsatile pattern after exercise likely facilitates its hypertrophic function in differentiated adult muscle cells.

- Global protein-coding gene expression changes peak at 8 h after an acute hypertrophic stimulus in human skeletal muscle, and early DNA methylation responses predict later gene expression changes.
- MYC is a key transcription factor regulating gene expression after resistance exercise, specifically during late recovery (8–24 h).
- Genetically controlled pulses of *Myc* in skeletal muscle of mice are sufficient for hypertrophy.

## Abstract (web summary)

A 24-h time course of recovery after an acute hypertrophic stimulus—resistance exercise—unravels the temporal dynamics of gene expression in human skeletal muscle. *MYC*’s pulsatile pattern after exercise likely facilitates its hypertrophic function in differentiated adult muscle cells.

## Graphical abstract

**Figure:**
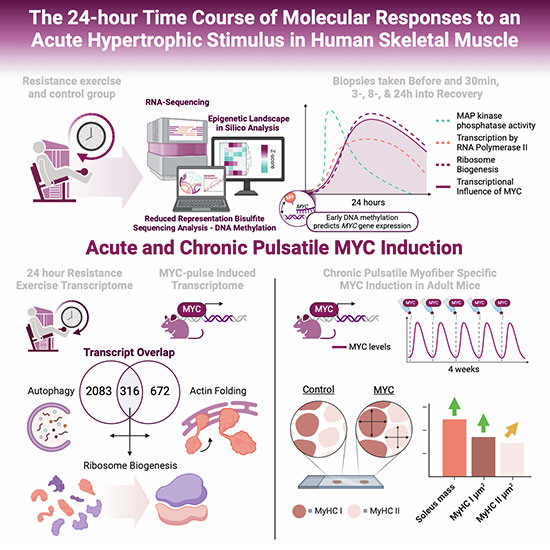

**Figure:**
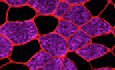

## Introduction

Molecular alterations after a bout of exercise in skeletal muscle precede hypertrophic adaptation and ultimately contribute to a change in phenotype (Egan et al, 2013; Egan and Sharples, 2022; Gustafsson et al, 1999; Jozsi et al, 2000; Kraniou et al, 2000; Perry et al, 2010; Pilegaard et al, 2000). Initial time course work in humans that used ≥3 post-exercise muscle biopsies established 2-4 h into recovery as the ideal time point for studying targeted changes in mRNA levels after a bout of exercise (Louis et al, 2007; Pilegaard et al, 2003; Vissing et al, 2005; Yang et al, 2005). Others that leveraged more comprehensive profiling of global gene expression (Mahoney et al, 2005; Neubauer et al, 2014; Zambon et al, 2003) demonstrated that many genes have delayed and/or biphasic responses to exercise in muscle that extend beyond 4 h. Recent work in skeletal muscle further emphasizes that gene expression data from a single time point after exercise is limiting when trying to capture the complex and dynamic nature of the adaptive response, and could even lead to inaccurate or misleading conclusions (Kuang et al, 2022). It is also important to consider the effects of the muscle biopsy (Vissing et al, 2005) and circadian rhythm (Zambon et al, 2003) in human exercise studies; these factors are typically overlooked. There is a critical need for temporally resolved and biopsy-only controlled investigations to explore and understand the molecular responses to resistance exercise since muscle mass and function is strongly associated with all-cause mortality (Isoyama et al, 2014; Li et al, 2018; Metter et al, 2002; Newman et al, 2006). A detailed understanding of the most influential molecular factors during the post-resistance exercise recovery period will help focus efforts at developing targeted therapies against muscle mass loss and/or enhancing hypertrophic responsiveness to exercise interventions.

Several seminal (Alway, 1997; Armstrong and Esser, 2005; Chen et al, 2002) and recent studies (Murach et al, 2022; Viggars et al, 2022a) suggest that the transcription factor *c-Myc* (referred to as *Myc* or *MYC* for mouse and human genes, respectively) is a key component of skeletal muscle hypertrophic adaptation to loading in animals. Our work using human skeletal muscle biopsies after a bout of resistance exercise (RE) (Figueiredo et al, 2021), as well as meta-analytical information that combines numerous human muscle gene expression datasets during the recovery period after exercise (Pillon et al, 2020), indicates that *MYC* is highly responsive to hypertrophic loading (Jones et al, 2022). MYC protein accumulates in human muscle following a bout of RE (Broholm et al, 2011; Brook et al, 2016; Figueiredo et al, 2016; Townsend et al, 2016) as well as in response to chronic training (Stec et al, 2016). Its expression may also differentiate between low and high hypertrophic responders (Stec et al, 2016). *Myc* is induced cell-autonomously in myotubes by electrical stimulation in vitro (Sidorenko et al, 2018) and is strongly upregulated in murine myonuclei during mechanical overload (Murach et al, 2022). MYC protein localizes to myonuclei during loading-induced hypertrophy (Alway, 1997; Armstrong and Esser, 2005), is considered pro-anabolic (Dang, 1999, 2012; Das et al, 2022), and can drive muscle protein synthesis and ribosome biogenesis in skeletal muscle (Brook et al, 2016; Mori et al, 2020; Wen et al, 2016; West et al, 2016). Loss of MYC results in lower muscle mass in preclinical models (Demontis and Perrimon, 2009; Wang et al, 2023). MYC is also estimated to target ~15% of the genome across different tissues and species (Dang et al, 2006). Still, the magnitude of its contribution to the exercise response in humans is not entirely understood (Phillips et al, 2013), nor is its sufficiency for muscle hypertrophy in preclinical models.

The current investigation details the global gene expression response to a bout of RE after 30 min, 3-, 8-, and 24-h using RNA-sequencing (RNA-seq) in skeletal muscle biopsy samples from healthy untrained humans. We reveal the effect of the muscle biopsy and inherent circadian rhythmicity using a biopsy-only, feeding and time point-matched control group. The human transcriptional time course data is provided in a publicly available user-friendly web-based application at http://data.myoanalytics.com. We then analyzed the human muscle methylome at 30 min after RE and combined these data with the transcriptome response to RE using a novel -omics integration approach. Integration of methylomics and transcriptomics sheds light on the molecular regulation of gene expression during recovery from exercise. With our transcriptome data, we infer the major transcriptional regulators of the exercise response using in silico ChIP-seq (Qin et al, 2020) that we have previously validated in skeletal muscle with a genetically modified mouse (Jones et al, 2022; Murach et al, 2022). These molecular and computational analyses identified *MYC* as an influential transcription factor controlling the exercise transcriptome throughout the time course of recovery after a bout of RE. Muscle-specific *Myc* overexpression data from the plantaris (Murach et al, 2022) and soleus (Jones et al, 2022) of mice reinforced the human exercise data. We employed a genetically modified mouse model to induce MYC in a pulsatile fashion specifically in skeletal muscle over 4 weeks to determine if MYC is sufficient for hypertrophy. Our genetically driven pulsatile approach avoids potential negative effects of chronically overexpressing a hypertrophic regulator (Castets et al, 2013; Ham et al, 2020) and more closely mimics the transient molecular response of exercise in skeletal muscle (Egan et al, 2013; Egan and Sharples, 2022; Perry et al, 2010; Smith et al, 2023). This work collectively illustrates the molecular landscape with temporal resolution after a bout of RE and places *MYC* at the center of the skeletal muscle RE response in mice and humans.

## Results

### Biopsy time course at rest and the transcriptional regulation of circadian genes in human skeletal muscle

**Figure 1:**
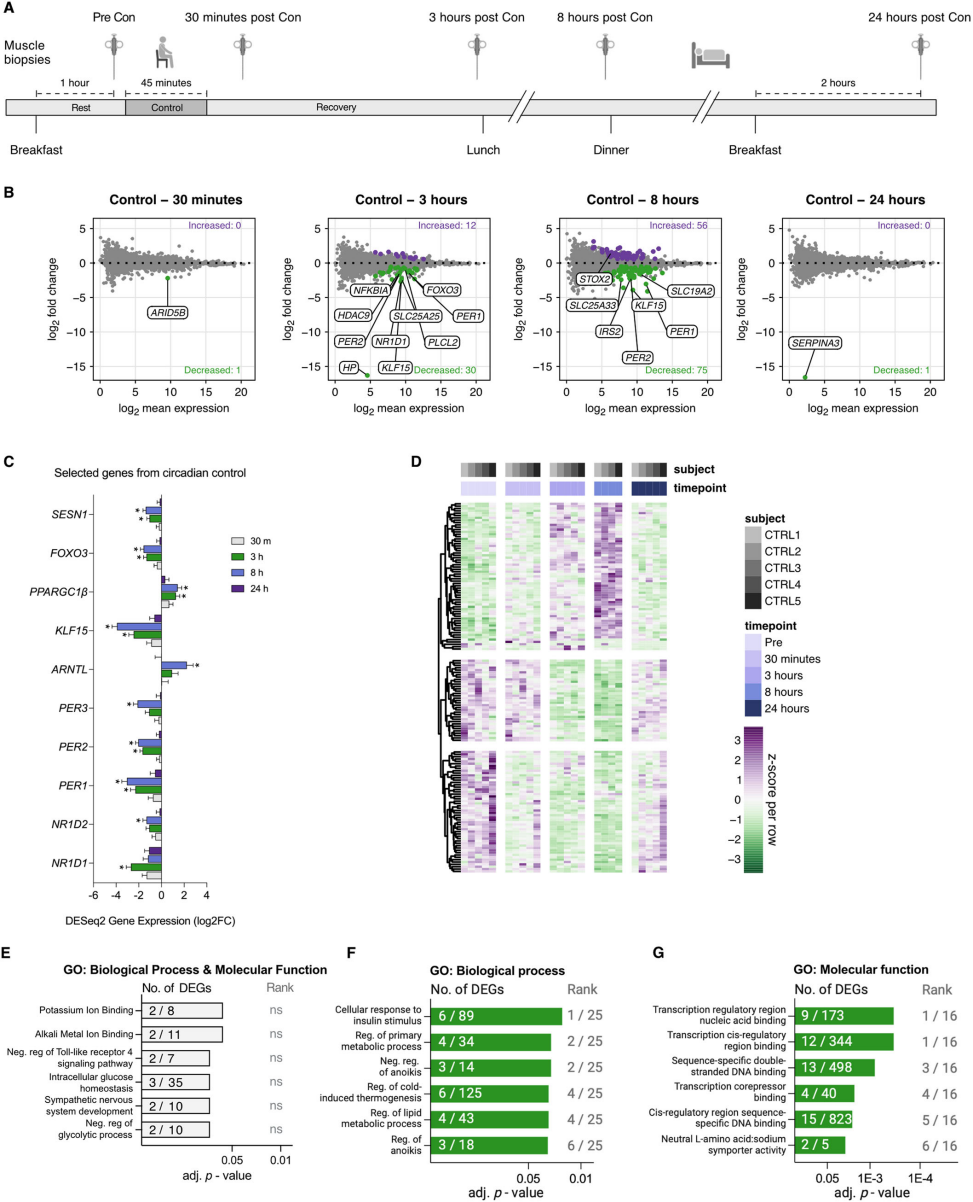
Gene expression patterns for biopsy-only control time course. (**A**) Schematic overview for the control arm of the human intervention, *n* = 5. (**B**) MA plots showing differentially expressed genes (DEG) vs pre-values, time matched to 30 min, 3, 8, & 24 h of recovery in the resistance exercise trial (see Fig. 2A). Purple and green dots indicate up- or downregulated regulated genes (adj. *p* < 0.05), respectively. Top genes for adj. *p*-value are highlighted in plots. (**C**) Fold-change for targeted DEGs in the control group, *n* = 5. *adj. *p* < 0.05. Values represent log_2_ fold-change ± SEM. *SESN1p* = 0.0034 at 3 h, *p* = 8.7E−6 at 8 h, FOXO3 adj. *p* = 0.0004 at 3 h, adj. *p* = 7.7E−6 at 8 h, *PPARGC1B* adj. *p* = 0.0486 at 3 h, adj. *p* = 0.0110 at 8 h, *KLF5* adj. *p* = 1.0E−5 at 3 h, adj. *p* = 3.5E−14 at 8 h, *ARNTL* adj. *p* = 0.0120 at 8 h, *PER3* adj. *p* = 7.8E−6 at 8 h, *PER2* adj. *p* = 6.8E−9 at 3 h, adj. *p* = 7.3E−13 at 8 h, *PER1* adj. *p* = 8.3E−5 at 3 h, adj. *p* = 3.0E−8 at 8 h, *NR1D2* adj. *p* = 0.0058 at 8 h, *NR1D1 p* = 1.1E−7 at 3 h. (**D**) Heatmap showing z-scores for 60 up-, and 90 downregulated DEGs across all time points and volunteers in the control trial. (**E**, **F**) Gene ontology (GO) gene set enrichment analysis on DEGs across the entire 24-h control period. Numbers within the bars indicate the proportion of DEGs in our dataset corresponding to the specific gene set. Rank values indicate the specific gene sets adj. *p*-value rank. (**E**) Upregulated Biological Processes and Molecular Functions, (**F**) Downregulated Biological Functions, (**G**) Downregulated Molecular Functions. (**B**, **C**) DESeq2 was calculated using a Wald test with a Benjamini–Hochberg *p*-value correction. (**E**–**G**) Gene ontology (GO) gene set enrichment analysis is analyzed using a Fisher exact test with Benjamini–Hochberg *p*-value correction. Con control situation, ns not significant, Neg. negative, Reg. regulation. Source data are available online for this figure.

**Figure 2:**
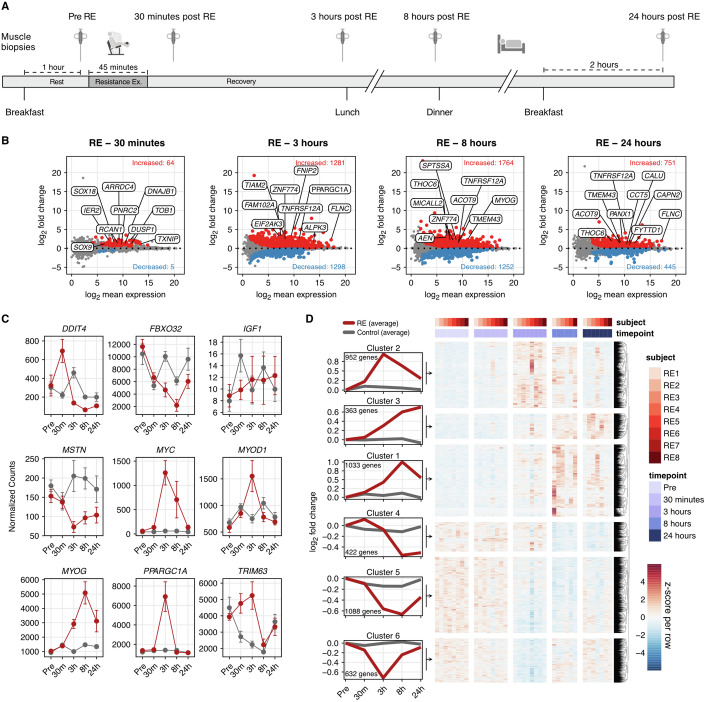
Gene expression patterns during 24 h of recovery from resistance exercise. (**A**) Schematic overview of resistance exercise (RE) intervention, *n* = 8. (**B**) MA plots showing differentially expressed genes (DEG) vs resting pre-values, following 30 min, 3, 8, & 24 h of recovery from RE (adj. *p* < 0.05). DESeq2 was calculated using a Wald test with a Benjamini–Hochberg *p*-value correction. Red and blue dots indicate up- or downregulated regulated genes (adj. *p* < 0.05), respectively. Top 10 genes for adj. *p*-value are highlighted in plots. (**C**) Normalized counts for targeted genes across the 24-h intervention, *n* = 8. Values represent normalized counts ± SEM. Red dots = RE trial, gray dots = Control trial. (**D**) Heatmap showing z-scores for 2399 up-, and 2126 downregulated DEGs across all recovery time points and volunteers in the RE trial. Genes are clustered according to their expression pattern across time points within the 24-h recovery period. (**B**–**D**) DESeq2 was calculated using a Wald test with a Benjamini–Hochberg *p*-value correction.

For the analysis of RNA-seq data, we focused on protein-coding genes as they are the most well-characterized. The results of these mRNAs are presented in Dataset EV1 and EV2. The Pre muscle biopsies were taken 15 min prior to the 45-min control protocol (equivalent to the 45 min of resistance exercise in RE group; Figs. 1A and 2A). Thus, in practice, the biopsy named 30 min post is taken 90 min after the Pre biopsy (15 min plus, 45 min, plus 30 min), the biopsy named 3 h is taken 4 h after the Pre biopsy, and so on. The biopsy time points, and their naming are chosen to precisely correspond to the post RE time points for the RE group (Fig. 2A). The first muscle biopsies were obtained within a 3.5 h window beginning at 7:30 AM for all participants in the study.

Differentially expressed genes (DEGs, adj. *p* < 0.05) were analyzed relative to the collected Pre biopsy. In the control group (CON, *n* = 5) the number of DEGs at the different recovery time points was: 30 min—0 upregulated, 1 downregulated; 3 h—12 upregulated, 30 downregulated; 8 h—55 upregulated, 75 downregulated; and 24 h—0 upregulated, 1 downregulated (Fig. 1B,D). Thus, the most protein-coding DEGs were observed at 8 h relative to Pre. Previous work involving human skeletal muscle biopsies revealed the rhythmic expression of muscle circadian core clock genes over 24 h (Perrin et al, 2018). We found that *NR1D1* (*REVERBα*) (adj. *p* = 0.01 × 10^−5^), *PER1* (adj. *p* = 0.0008), and *PER2* (adj. *p* = 0.007 × 10^−6^) were lower at the 3-h time point (Fig. 1C). At 8 h, *NR1D2* (*REVERBβ*) (adj. *p* = 0.005), *PER1* (adj. *p* = 0.03 × 10^−6^), *PER2* (adj. *p* = 0.007 × 10^−10^), and *PER3* (adj. *p* = 0.007 × 10^−3^) were lower, while *ARNTL*, also known as *BMAL1*, was upregulated (adj. *p* = 0.01) (Fig. 1C). *KLF15*, a circadian-regulated mediator of lipid metabolism (Perrin et al, 2018), was lower at the 3-h (adj. *p* = 0.0001) and 8-h time points (adj. *p* = 0.03 × 10^−12^) in the CON group (Fig. 1C). *PPARGC1β*, another circadian-controlled gene (Gidlund et al, 2015; McCarthy et al, 2007), was upregulated at 3 h (adj. *p* = 0.048) and 8 h (adj. *p* = 0.01; Fig. 1C). Also worth mentioning is that *FOXO3*, a central regulator of autophagy and mass in skeletal muscle (Mammucari et al, 2007; Sandri et al, 2006; Zhao et al, 2007), was lower at 3 h (adj. *p* = 0.0004) and 8 h (adj. *p* = 0.007 × 10^−3^, Fig. 1C). *SESN1*, which may also regulate muscle mass (Segalés et al, 2020), was lower at 3 h (adj. *p* = 0.003) and 8 h (adj. *p* = 0.008 × 10^−3^) (Fig. 1C).

To more broadly investigate what specific functions were being regulated as a consequence of our CON intervention, we ran background corrected gene set enrichment analysis using Enrichr with the 2023 gene ontology (GO) database as our cross reference (GO: biological process & molecular function) and DEGs combined across all time points (Aleksander et al, 2023; Chen et al, 2013; Kuleshov et al, 2016; Stokes et al, 2023; Xie et al, 2021). Collectively, no significantly upregulated gene sets were detected (Fig. 1E). A few negatively regulated GO-based gene sets were detected, indicating a reduced “cellular response to insulin” and reduced “transcriptional speed” (Fig. 1F,G). Although significantly enriched, only 2–15 genes were coding for each gene set, suggesting the effect was small. Nevertheless, it should be noted that the significantly downregulated gene sets are almost exclusively driven by DEGs expressed at 3 and 8 h. In addition to circadian rhythmicity of gene expression, DEGs across time points in CON could be related to feeding since the Pre, 30-min, and 24-h biopsies were taken 1, 2, and 2 h after feeding, respectively, while the biopsies at 3 and 8 h were both taken approximately 5 h after food intake (Fig. 1A). Thus, our finding of slightly downregulated insulin stimulation response and transcription genes at 3 and 8 h seems intuitive.

### Differentially expressed genes peaked 8 h after resistance exercise (RE) relative to pre

Following an acute bout of resistance exercise (RE, *n* = 8, Fig. 2A), the number of DEGs relative to the Pre time point (adj. *p* < 0.05) was: 30 min—64 upregulated, 5 downregulated; 3 h—1281 upregulated, 1298 downregulated; 8 h—1764 upregulated, 1253 downregulated; and 24 h—751 upregulated, 445 downregulated (Fig. 2B). Over 90% of the transcriptomic signatures were estimated to originate from myofibers irrespective of time point. This proportion was estimated using CIBERSORTx (Newman et al, 2019), a computational cellular devonvolution tool that we have used previously for inferring changes in cell type after an acute hypertrophic stimulus (Murach et al, 2022). A recent acute exercise and single-cell RNA-seq study in human skeletal muscle was used as the reference dataset (Lovrić et al, 2022). The exercise involved intense cycle sprint intervals and the muscle sampled was the vastus lateralis, which corresponds with our study design. In this dataset (Lovrić et al, 2022), “myocytes” were inferred to be myonuclei based on the expression of adult myosin heavy chains and other muscle fiber-enriched markers. Myonuclei usually appear in skeletal muscle single-cell datasets and are sequenced alongside mononuclear cells (McKellar et al, 2021; Murach et al, 2021c). When excluding “myocytes” (myofibers), no appreciable change in mononucleated cell proportions was estimated throughout recovery (Fig. EV1). As anticipated, we noted changes in genes previously recognized as responsive to RE and/or important for muscle remodeling (Fig. 2C) (Correia et al, 2023; Ferreira et al, 2019; Figueiredo et al, 2021; Pillon et al, 2020). Of all upregulated protein-coding genes across the 24-h recovery period in the RE group, 46% were differentially expressed at two or more time points while the proportion was 34% for downregulated genes. In total, 2399 unique upregulated and 2126 unique downregulated DEGs were identified throughout the 24-h recovery period (Fig. 2D). DEGs at each time point relative to Pre are presented in Dataset EV2.

**Figure EV1:**

Cell composition of muscle biopsies through data deconvolution. Cell composition of all skeletal muscle biopsy time points excluding “myocytes” (CTRL *n* = 5, RE *n* = 8). The box represents the 25th–75th percentile, the line represents the median, and the whiskers represent Min to Max, excluding outliers. SC satellite cell, CTRL control group, RE resistance exercise group.

### The integrated 24-h recovery transcriptome after acute RE

Using the two lists of all DEGs from across the entire 24-h recovery period after RE (up- or downregulated) relative to Pre generated in Fig. 2D (Complete list; Dataset EV2), we employed background corrected gene set analysis as described above on each list separately (Aleksander et al, 2023; Chen et al, 2013; Kuleshov et al, 2016; Stokes et al, 2023; Xie et al, 2021).

**Figure 3:**
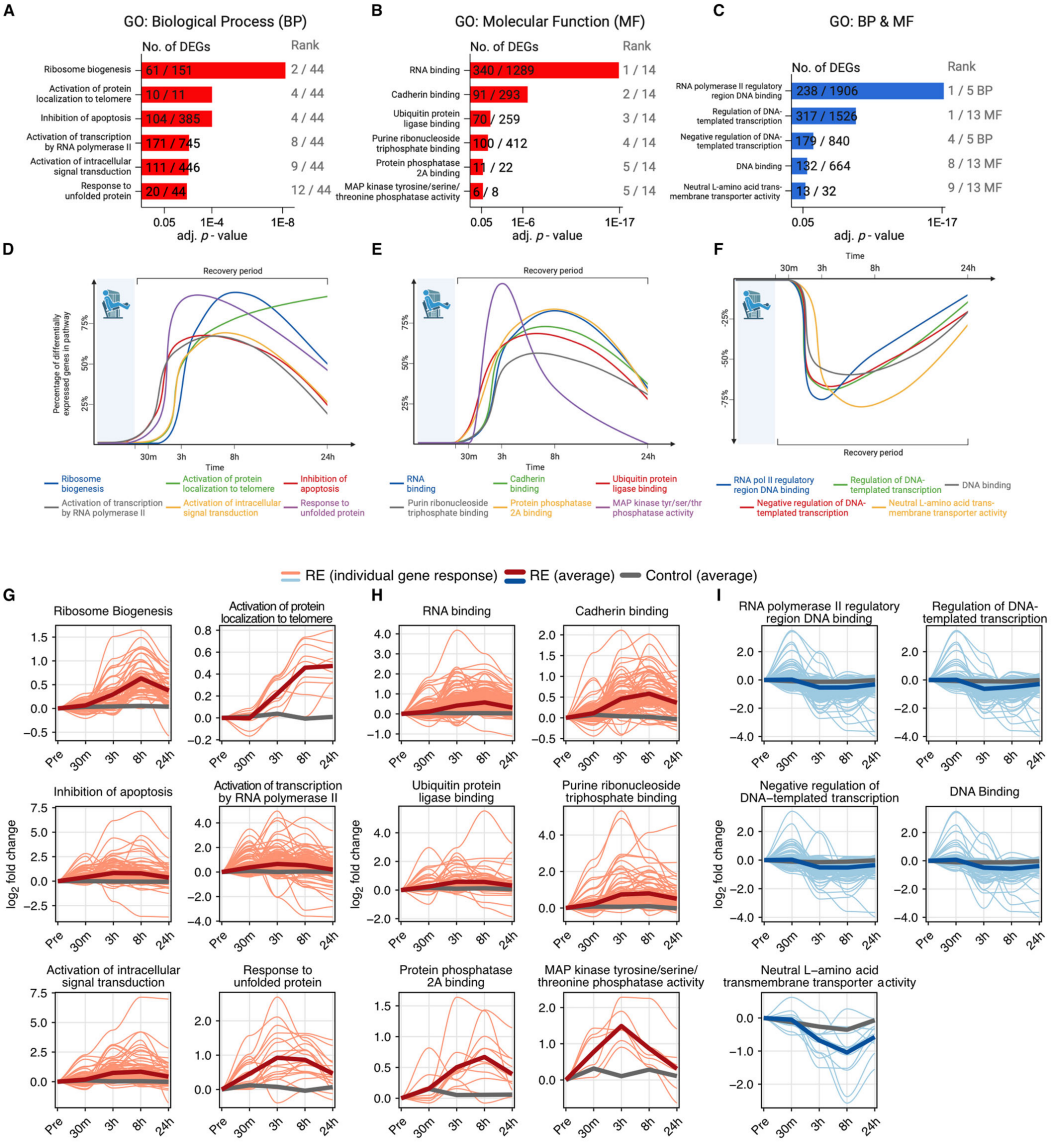
Gene set enrichment time course across 24 h of recovery from resistance exercise. (**A**–**C**) Gene ontology (GO) gene set enrichment analysis on DEGs across the entire 24-h recovery period. Numbers within the bars indicate the proportion of DEGs in our dataset corresponding to the specific gene set. Rank values indicate the specific gene sets adj. *p*-value rank. The corresponding timeline shows the proportion of DEGs with the specific gene sets across the 24-h recovery period. (**A**) Upregulated Biological Processes, (**B**) Upregulated Molecular function, and (**C**) Downregulated Biological Processes and Molecular function. (**D**–**F**) Timelines of the top (GO) gene sets from (**A**–**C**) expressed as a percentage of the number of DEGs within that gene set. (**D**) Upregulated Biological Processes, (**E**) Upregulated Molecular function, and (**F**) Downregulated Biological Processes and Molecular function. (**G**–**I**) Average fold-change (vs Pre) for highlighted gene sets (thick red lines) along individual DEGs within the specific gene set (lighter red lines). The average fold-change for the same genes in the control situation is presented in gray. (**G**) Upregulated Biological Processes, (**H**) Upregulated Molecular function, (**I**) Downregulated Biological Processes and Molecular Function. (**A**–**C**) Gene ontology (GO) gene set enrichment analysis is analyzed using a Fisher exact test with Benjamini–Hochberg *p*-value correction. Source data are available online for this figure.

For the 2399 upregulated protein-coding DEGs across the entire time course of recovery, 44 biological processes (adj. *p* < 0.05) were identified, with a large proportion of the gene sets in the 24-h post-RE window relating to transcription, translation, and the synthesis of new ribosomes. After exclusion of gene sets with large overlaps in underlying DEGs, the top (adj. *p*-value ranked) processes were ribosome biogenesis (GO:0042254), activation of protein localization to telomere (GO:1904816), inhibition of apoptosis (GO:0043066), activation of transcription by RNA polymerase II (GO:0045944), activation of intracellular signal transduction (GO:1902533), and response to unfolded protein (GO:00066986) (Fig. 3A). Fourteen molecular function gene sets were also identified (adj. *p* < 0.05). Of the molecular functions identified, RNA binding (GO:0003723), cadherin binding (GO:0045296), ubiquitin protein ligase binding (GO:0031625), purine ribonucleoside triphosphate binding (GO:0035639), protein phosphatase 2A binding (GO:0051721), and MAP kinase tyrosine/serine/threonine phosphatase activity (GO:0033550) were the top gene sets, again excluding gene sets with large overlap (Fig. 3B).

Next, we identified at which time point all DEGs within each specific gene set were differentially upregulated relative to Pre. Using the gene set analysis from the entire time course of recovery, the number of upregulated DEGs in a gene set at each specific time point was expressed as a percentage of the entire gene set response (e.g., 61 DEGs in our dataset were found to regulate ribosome biogenesis, and of these 61 genes, 57—or 93%—were enriched following 8 h of recovery). Plotting these values for each individual time point relative to Pre thus revealed a 24-h temporal pattern of each gene set following acute RE (Fig. 3D,E). None of the most highly enriched gene sets within our analysis peaked at 30 min post-exercise. However, a targeted analysis of enriched gene sets with peak expression at 30 min revealed growth factor- and glucocorticoid responses are strongest at 30 min post-RE, as well as stress response signaling and mRNA catabolism (Fig. EV2A). The peak in transcripts coding for mRNA catabolism 30 min after RE (*BTG2*; adj. *p* = 0.005, *ZC3H12A*; adj. *p* = 0.003, *ZFP36L1*; adj. *p* = 0.01, and *TOB1*; adj. *p* = 0.0005) precedes any marked downregulation of DEGs, suggesting catabolism of mRNA occurs in muscle following upregulation of anti-proliferative- and mRNA-decaying enzymes.

**Figure EV2:**

Targeted gene set enrichment analysis. Pooled gene ontology (GO) biological processes and molecular function gene sets. (**A**) Targeted analysis of gene sets peaking at 30 min post resistance exercise (RE). (**B**) Targeted analysis of gene sets significantly enriched in genes downregulated 3–8 h post RE. (**C**) Targeted analysis of biphasic DEGs composing cluster 4, as presented in Fig. 2D. *NR4A1* adj. *p* = 0.0049 at 30 min, adj. *p* = 6.5E−8 at 8 h, adj. *p* = 1.0E−6 at 24 h, *NR4A2* adj. *p* = 0.0135 at 30 min, adj. *p* = 0.0081 at 3 h, adj. *p* = 4.7E−6 at 8 h, adj. *p* = 2.4E−6 at 24 h, *NR4A3* adj. *p* = 1.8E−8 at 8 h. (**A**–**C**) Gene ontology (GO) gene set enrichment analysis is analyzed using a Fisher exact test with Benjamini–Hochberg *p*-value correction. (**D**) Gene expression of selected genes with a biphasic gene expression pattern, up early/down late, *n* = 8. DESeq2 was calculated using a Wald test with a Benjamini–Hochberg *p*-value correction. *p < 0.05 vs Pre values. Neg. negative, Reg. regulation.

At 3 h post-RE relative to Pre, we observed two major upregulated gene sets that were peaking: response to unfolded proteins (20/44 genes; Fig. 3A,D) and MAP kinase phosphatase activity (6/8 genes; Fig. 3B,E). The former of the two is primarily driven by genes coding the heat shock protein family, such as *DNAJA1* (adj. *p* = 8.5 × 10^−7^ at 3 h) and *HSPA1A* (adj. *p* = 8.5 × 10^−5^ at 3 h). The latter, MAP kinase phosphatase activity, is driven by genes encoding the dual specificity phosphatase protein family (DUSP), responsible for dephosphorylation of tyrosine/serine/threonine sites (*DUSP2, 4, 5, 8, 10* & *16*, adj. *p* < 0.05 at 3 h). DEGs within this gene set peaked at the 3-h time point, with only two remaining elevated at 8 h. This pattern was also reflected when mapping the fold change of DEGs within each gene set, rather than the number of DEGs, across the 24-h recovery (Fig. 3G,H). A clear peak in genes encoding phosphatase activity directed towards the MAP kinase superfamily early during RE recovery may be a response triggered by the rapid severalfold increase in protein phosphorylation of mTOR-targets such as S6K1 and 4EBP1 occurring at around 60–90 min post-RE (Apró et al, 2015; Moberg et al, 2016). According to this previous work, the rapid rise in anabolic signaling via protein phosphorylation at this time point is then followed by a swift decrease, with some signaling proteins showing close to baseline phosphorylation levels at 3 h of recovery (Apró et al, 2015; Moberg et al, 2016).

Several upregulated gene sets are overrepresented to a similar degree at 3 and 8 h of recovery such as ubiquitin protein ligase binding (70/259 genes; GO:0031625; Fig. 3B,E,H), activation of transcription by RNA polymerase II (171/745 genes; GO:0045944; Fig. 3A,D,G), and activation of intracellular signal transduction (111/446 genes; GO:1902533; Fig. 3A,D,G), pointing to increased protein turnover. At the same time, inhibition of apoptosis (104/385 genes; GO:0043066; Fig. 3A,D,G) was also upregulated, which may be a direct response to the increased transcriptional emphasis on the ubiquitin system. The post-translational modifications mediated by ubiquitination of pro-apoptotic Bcl-2 family and BH3-only proteins have been proposed to be crucial for cell survival (Roberts et al, 2022). For instance, the transcript *RNF144B* coding for the E3 ubiquitin ligase *IBRDC2*, which targets the Bcl-2 ‘executioner’ Bax for ubiquitination (Benard et al, 2010), is significantly upregulated at 3 h only (adj. *p* = 2.8 × 10^−7^).

Following the burst of transcription- and translation initiation-coding transcripts at 3 and 8 h, the upregulated mRNA landscape shifted toward the ribosome. At 8 and 24 h of recovery, regulation of ribosome biogenesis (61/151 genes; GO:0042254; Fig. 3A,D,G) and RNA binding (340/1289 genes; GO:0003723; Fig. 3B,E,H) appear to be the dominant gene sets. Within the RNA binding gene set, genes supporting ribosome assembly, posttranslational control of RNA, splicing via RNA-binding motif protein family members (e.g., *RBMX*, *RBM15*, *RBM39*), heterogeneous nuclear ribonucleoproteins (e.g., *HNRNPU*, *HNRNPR*, *HNRNPC*) and zinc fingers (e.g., *ZNF326*, *ZNF579, ZNF697*) were differentially expressed. Moreover, several transcripts coding ribosome biogenesis factors (e.g., *BMS1* and *LTV1*) as well as ribosomal assembly and transport proteins (e.g., *NIP7*, *NOP14*, *RPF2*) comprised the highly enriched ribosome biogenesis gene set (GO:0042254).

Gene sets enriched within the 2126 downregulated DEGs were considerably fewer compared to the upregulated genes (Fig. 3C,F,I). Here, five biological processes and 13 molecular functions were downregulated (adj. *p* < 0.05). Out of the five significant biological processes, four were related to transcription. The majority of DEGs within these transcription-related gene sets were classified as inhibitors of transcription, meaning RE likely acts on transcription by up-regulating activation (Fig. 3A) and by repressing suppressor genes (Fig. 3C) to a similar extent. In addition to transcription, histone H3 methyltransferase activity was one of the gene sets found to be significantly downregulated at 3 and 8 h following RE using a targeted analysis of these specific time points (Fig. EV2B).

### Information on genes with divergent responses throughout the time course of RE recovery

Specifically focusing on genes that were upregulated early after RE and downregulated later relative to Pre (Cluster 4; Fig. 2D), we found that some of the negative regulators of RNA Pol II transcription (GO:0045892, GO:0000122) followed this pattern—upregulated 30 min and/or 3 h while downregulated later at 8 and 24 h (Fig. EV2C). Nuclear receptor subfamily 4 group A genes *NR4A1* and *NR4A2* were among the 10 transcripts in the sequence-specific DNA binding gene set (GO:0043565) that most clearly followed a biphasic expression pattern (up early, down late; Fig. EV2D). Related to *NR4A1* and *NR4A2*, *NR4A3* showed similar biphasic tendencies, being upregulated at 3 h (4.93 log_2_FC, adj. *p* < 0.05) and shifting toward lower expression at 24 h relative to Pre (−0.78 log_2_FC, adj. *p* > 0.05; Fig. EV2D). This finding is in agreement with previous reports suggesting *NR4A* family transcripts are highly responsive early during exercise recovery (Amar et al, 2021; Pillon et al, 2020). Others have also shown that *NR4A3* is upregulated by seemingly opposing stimuli—following both acute exercise as well as long-term inactivity (Amar et al, 2021; Pillon et al, 2020). Many acute exercise interventions only sample muscle for 3–5 h during recovery, so a delayed depression of the *NR4A* genes (at 24 h into recovery) due to the biphasic nature of this gene family (Fig. EV2D) may be underappreciated. An interpretation could be that exercise ubiquitously drives *NR4A* expression, while in fact, the post-exercise induction and repression could be balanced, and this pattern may have a specific biological function pertaining to exercise adaptation. Regardless, the existence of biphasic genes within 24 h of RE recovery highlights the importance of considering muscle biopsy sampling time points when interpreting data.

### Harmonizing the biopsy-only time course with the RE recovery time course

The biopsy-only group that did not undergo exercise provides a lens into the effects of circadian rhythmicity, diet, and/or the effect of the muscle biopsy and how this relates to the RE response. Out of the 60 upregulated, and 90 downregulated genes expressed across the 24-h recovery in the biopsy-only group, 28 and 58 genes respectively were similarly regulated in the RE group (Fig. EV3). Among them were circadian genes *PER1* and *PER3*, but not *PER2* or *PPARGC1β* (presented in Fig. 1C). By contrast, 8 upregulated DEGs in the control group were downregulated by RE, and 9 genes that were downregulated in the control group were instead upregulated by RE. Of the genes that overlapped between the biopsy-only group and the RE group (e.g., *KLF15, NR1D1, NR1D2, PER1, SESN1*), RE tended to blunt their excursions and increase variability. For example, at the 8-h time point, *KLF15* was −3.9 log_2_FC lower (adj. *p* = 0.04 × 10^−12^) relative to Pre in the biopsy-only group, whereas after RE at the same time point, *KLF15* was −1.9 log_2_FC lower relative to Pre (adj. *p* = 0.02 × 10^−4^) with a larger range of expression. As expected, the effect of RE outweigh any other effects with respect to overall gene expression, but there appears to be interaction between exercise and the biopsy, feeding, and or circadian rhythmicity across time points that should be considered when designing muscle biopsy studies.

**Figure EV3:**

Venn diagram illustrating overlap in gene expression between the biopsy-only control group and the post-RE response at 3 and 8 h. (**A**) Venn-diagram of up and downregulated differentially expressed gene lists from the resistance exercise and control group. RE resistance exercise. (**B**) Genes corresponding to the overlap presented in (**A**).

### Inferring fiber type distribution from the transcriptional data across cohorts

**Figure EV4:**
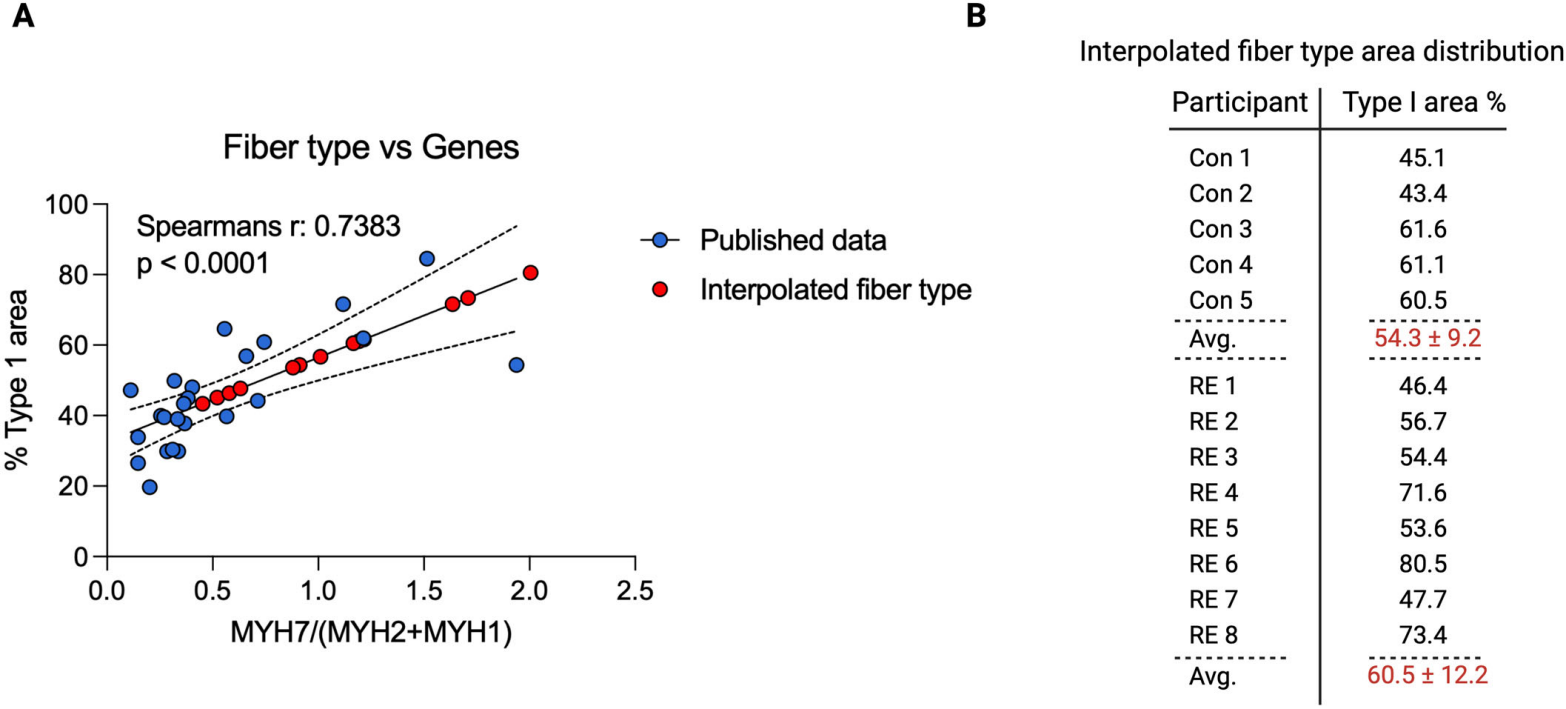
Interpolated fiber type area distribution of muscle samples. (**A**) Correlation of gene expression data with type I fiber area asses by muscle histology (*p* = 3.0E−5), data from Reitzner et al (2024). Blue dots = Data points from Reitzner et al (2024), Red dots = interpolated values based on gene data. (**B**) Data table of type I fiber area % in each participant.

To characterize the fiber type distribution of the RE and CON group, we used the gene counts of adult myosin heavy chain mRNAs (*MYH1*—Type IIX, *MYH2*—Type IIA, and *MYH7*—Type I) to interpolate skeletal muscle fiber type distribution. We accomplished this by leveraging a publicly available dataset that contained both transcriptional data and fiber type distribution using muscle histology (Reitzner et al, 2024). Correlating the % Type I fiber area from each individual to the MYH transcript ratio (*MYH7*/(*MYH2* + *MYH1*)) yielded a strong significant correlation of *r* = 0.7178 (*p* < 0.0001, Fig. EV4A). We then used these values to create a standard curve and inserted our *MYH*-transcript data along the fitted line, thus estimating a fiber-type distribution in our samples. The muscle samples from the CON group and RE group consisted of 54.3 ± 9.2 and 60.5 ± 12.2% type I fiber area (Fig. EV4B), respectively, with no difference between the two groups (*p* = 0.33).

### Changes to the muscle DNA methylome at 30 min of recovery after RE relates to mRNA responses at 3 h

Binding and expression target analysis (BETA) is a multi-omics integration method for understanding transcriptional regulation (Wang et al, 2013). We recently adapted this method for combining reduced representation bisulfite sequencing (RRBS) data with RNA-sequencing data to understand how DNA methylation regulates the transcriptome during an acute loading stimulus in mice (Ismaeel et al, 2023). We also used this technique to relate the methylome to the proteome after exercise training in skeletal muscle (Chambers et al, 2024). Briefly, BETA considers differential methylation status (both hypo- and hyper-methylation) in relation to transcription start sites using weighted scores to infer transcriptional regulation, which is then combined with transcriptomic data for validation. This method generates a regulatory potential score on a gene-by-gene basis as well as an overall *p* value for a cumulative distribution function (one-tailed Kolmogorov–Smirnov test) that discriminates global time point differences for up or down genes. In our recent work, myonuclear DNA methylation status coincided with changes in myonuclear gene expression as well as the acute metabolic responses that occurred during rapid muscle growth, giving us confidence in the validity of BETA (Ismaeel et al, 2023). We leveraged RRBS and RNA-sequencing data in the current study to provide a deeper understanding of transcriptional regulation in response to acute RE.

**Figure 4:**
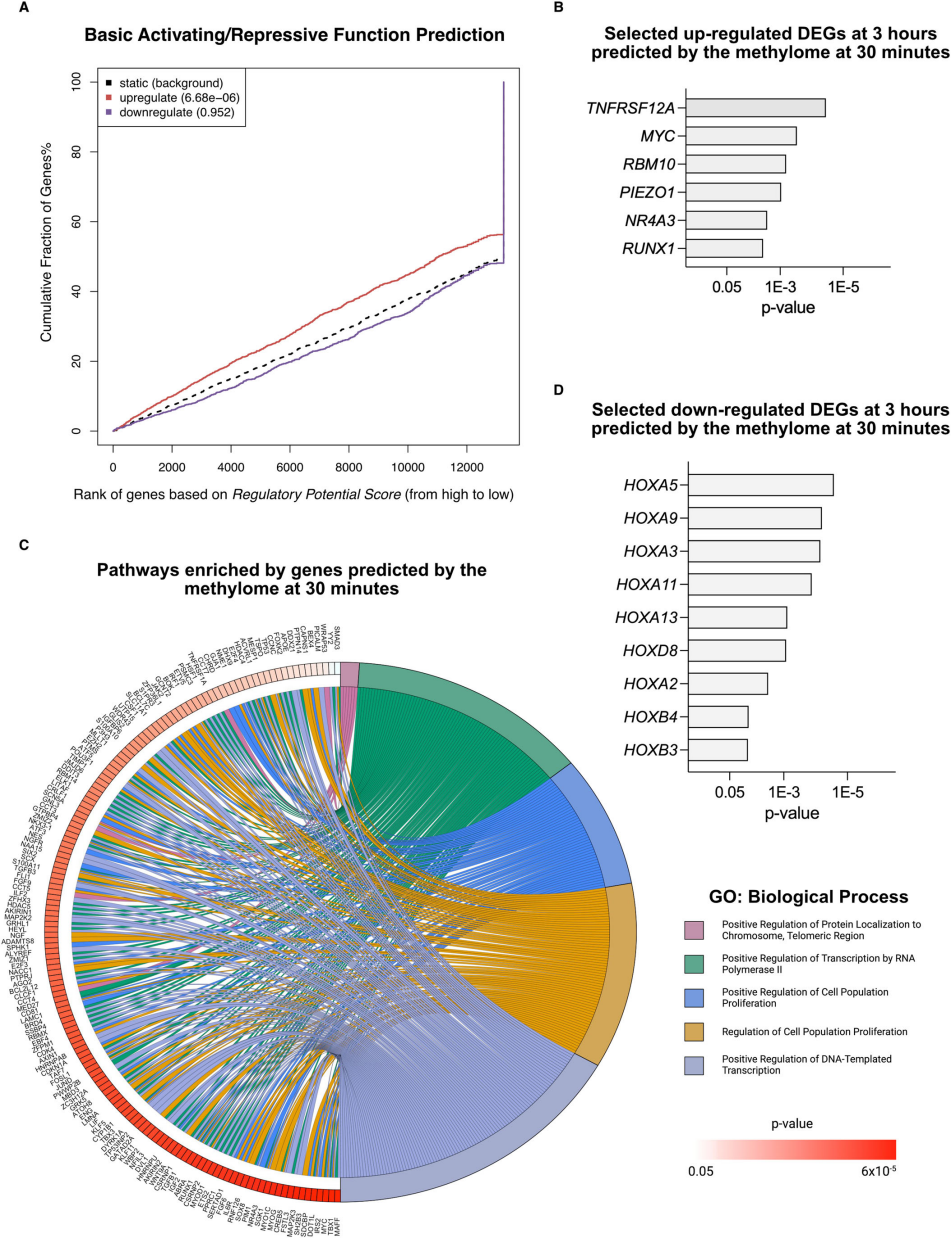
Immediate (30 min post-RE) DNA methylome changes after RE predict transcriptional regulation at 3 h. (**A**) Binding and expression target analysis (BETA) combining the DNA methylome at 30 min to differentially expressed genes (DEGs) at 3 h post resistance exercise (RE). BETA integration analysis of up- and downregulated genes to RRBS methylation performed relative to background with predictive interaction significance represented by *p*-values in parenthesis. (**B**) Selection of upregulated differentially expressed genes (DEGs) at 3 h post-RE significantly predicted by the methylome at 30 min. (**C**) Chord plot illustrating the top five biological processes (gene ontology) regulated at 3 h post exercise by genes predicted by the methylome at 30 min post exercise. Gene set-associated genes are ordered according to their *p*-values. (**D**) Selection of downregulated DEGs at 3 h post RE suggested being affected by methylation changes at 30 min post RE. (**A**–**D**) was calculated using a one-tailed Kolmogorov–Smirnov test, while Gene ontology (GO) gene set enrichment analysis for (**C**) was analyzed using a Fisher exact test with Benjamini–Hochberg *p*-value correction. Source data are available online for this figure.

We first used BETA to compare the methylome and transcriptome responses to RE at 30 min of recovery versus Pre (Dataset EV3 shows processed methylation data for 30 min post-RE versus Pre). Combining datasets at this time point revealed <10 genes were likely being regulated at the level of methylation. This result seems intuitive since changes in DNA methylation typically precede changes in gene expression (Barres et al, 2012), which does not peak until later time points in our data. As such, we combined the 30 min methylome data with the later transcriptome time points after RE. Changes to the methylome 30 min after RE were strongly predictive of the changes observed in gene expression at 3 h after RE versus Pre (Fig. 4A), but not later time points. This analysis inferred significant methylation control of 936 upregulated genes at 3 h (*p* = 0.000007), and 805 downregulated genes were identified according to BETA (*p* < 0.05), but the overall regulation of repressed genes was not significant according to the Kolmogorov–Smirnov test (*p* = 0.952). It is important to note that the lack of significance according to BETA for downregulated genes does not mean that methylation is not regulating gene expression on a gene-by-gene basis, but that the global regulatory potential score did not achieve significance. Thus, we present the BETA analysis for individual genes to provide additional insights.

Of upregulated genes with a coordinated methylome and transcriptome response, *TNFRSF12A* (*FN14*) was the most significant (*p* = 0.000035; Fig. 4B). Upregulation of the TWEAK receptor *FN14* occurs during the muscle hypertrophic response to exercise specifically in fast-twitch type 2 fibers of humans (Murach et al, 2014; Raue et al, 2012). This role for *FN14* induction during muscle adaptation could be related to non-canonical NF-*κ*B pathway activation (Raue et al, 2015). Furthermore, inhibition of *FN14* in human myotubes increases C/EPβ and MuRF (Walton et al, 2019). Alternatively, mechanistic work in rodents suggests *Fn14* knockout in muscle fibers improves endurance exercise capacity and inhibits neurogenic muscle atrophy (Tomaz da Silva et al, 2022), but ablation in satellite cells attenuates muscle regeneration (da Silva et al, 2023). More gain and loss of function studies are needed to clarify the role of *Fn14* in hypertrophic muscle adaptation (Dungan et al, 2022; Pascoe et al, 2020). Other notable genes with a coordinated upregulated response to RE included: *RUNX1* (Fig. 4B), which regulates muscle mass (Wang et al, 2005) and is enriched in myonuclei during rapid load-induced hypertrophy (Murach et al, 2022); *RBM10* (Fig. 4B), an RNA splicing factor that we previously showed is altered at the methylation level in muscle with late-life hypertrophic exercise in mice (Dungan et al, 2022; Murach et al, 2021a); and *NR4A3* (Fig. 4B), among the most exercise-responsive genes in skeletal muscle that controls metabolism (Pillon et al, 2020). We previously reported that promoter region CpG hypomethylation of *Myc* in myonuclei (von Walden et al, 2020) coincided with strong upregulation of myonuclear and muscle tissue *Myc* levels during acute mechanical overload in mice (Murach et al, 2022; von Walden et al, 2020). BETA also suggested coordinated methylation and transcriptional regulation of *MYC* by RE in human muscle here (Fig. 4B). Evidence in cancer cells indeed suggests that *MYC* transcription is regulated by DNA methylation status (Cheah et al, 1984; de Souza et al, 2013; Kaneko et al, 1985; Rao et al, 1989; Tsujiuchi et al, 1999), in addition to regulation by other epigenetic layers (Fan et al, 2016; Lüscher, 2001) and G-quadruplexes (Brooks and Hurley, 2010). Of the 936 methylation-controlled upregulated genes predicted by BETA, 155 were coding for five biological processes as suggested by gene set enrichment analysis (Fig. 4C).

BETA integration of 30-min methylome responses with 3-h transcriptome responses to RE was not significant overall for downregulated genes. However, gene-by-gene analysis revealed methylation control for widespread downregulation of HOX genes—*HOXA2*, *HOXA3*, *HOXA5*, *HOXA9*, *HOXA11*, *HOXA13*, *HOXB3*, *HOXB4*, and *HOXD8* (Fig. 4D). In muscle, *HOX* genes are highly regulated by DNA methylation (Tsumagari et al, 2013), and are methylation hotspots during aging that are influenced at the methylation and mRNA levels by physical activity in humans (Turner et al, 2020; Voisin et al, 2021). We previously reported methylation changes around *HOX* genes in myonuclei during hypertrophy (Murach et al, 2021b) and with exercise during aging in muscle tissue (Chambers et al, 2024; Murach et al, 2021a). *HOX* genes control muscle development (Alvares et al, 2003; Poliacikova et al, 2021), but little is known about their role in RE adaptation in adult skeletal muscle.

### MYC governs the late stage RE response via several processes

**Figure 5:**
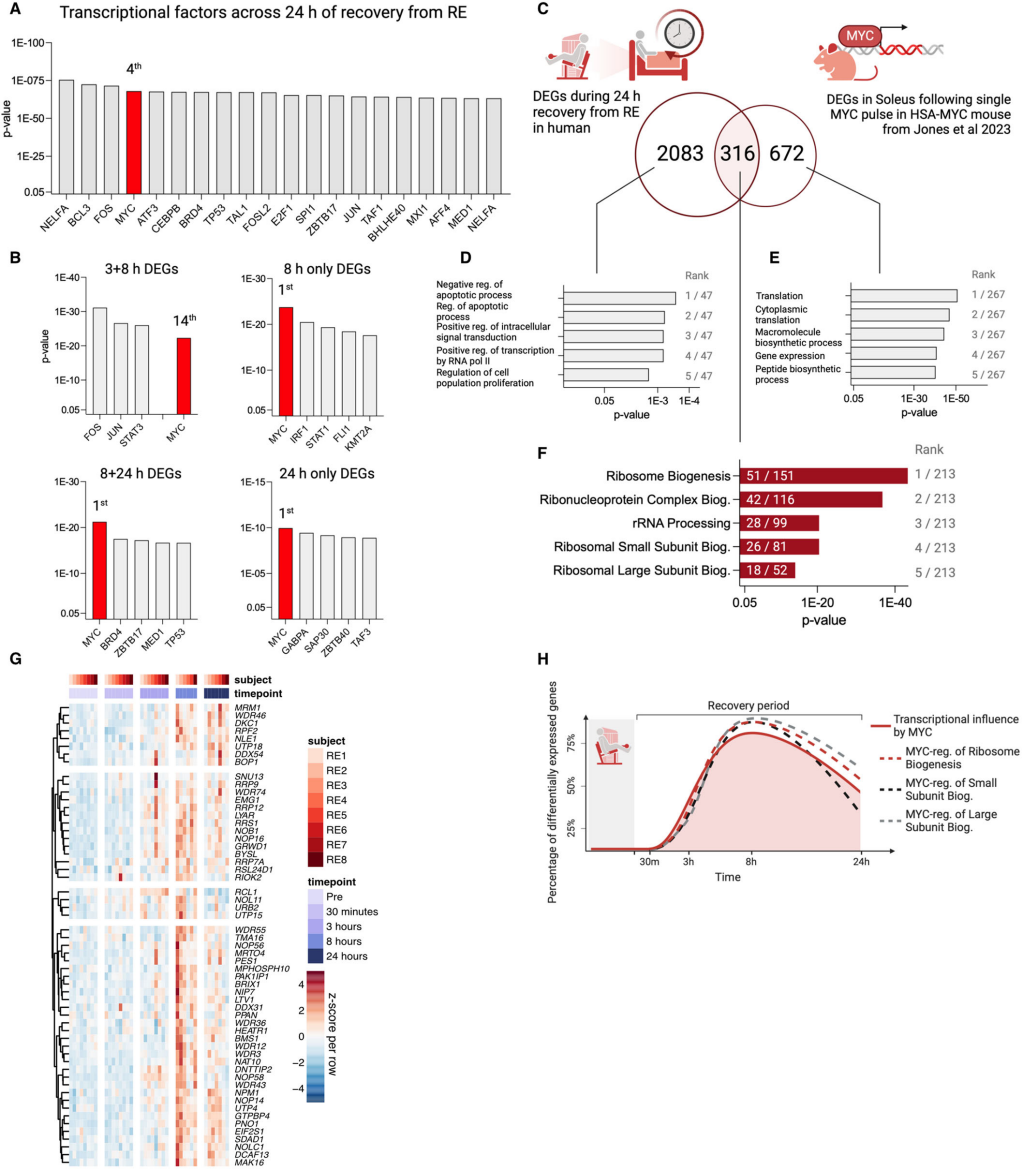
The transcription factor MYC dominates late-stage acute recovery from RE by regulating ribosome biogenesis. (**A**) Transcription factors predicted to be active during the 24-h recovery period from resistance exercise (RE) sorted by *p*-value. (**B**) Transcription factors predicted to regulate the genes expressed exclusively at the later stages of acute recovery. (**C**) Comparison of upregulated DEGs across 24 h of RE recovery in humans (*n* = 8) vs soleus muscle from MYC-overexpressing mice from Jones et al (2022). (**D**–**F**) The top five gene sets (GO: Biological Processes) based on DEGs in (**D**) the human exclusive gene list, (**E**) MYC mouse exclusive gene list, and (**F**) overlapping gene list, respectively. Gene sets are ranked according to their adj. *p*-values. (**G**) Heatmap showing DEG pattern for ribosome-related genes overlapping human RE response to a MYC response in mouse soleus muscle. Genes retrieved from all five gene sets presented in 3F. (**H**) Time courses for MYC’s transcriptional influence (Red solid line), as well as three MYC-regulated gene sets (dashed lines). (**A**, **B**) Is analyzed with epigenetic Landscape In Silico deletion Analysis (Lisa) using a one-sided Wilcoxon rank-sum test. (**D**–**F**) Gene ontology (GO) gene set enrichment analysis is analyzed using a Fisher exact test with Benjamini–Hochberg *p*-value correction. Source data are available online for this figure.

Due to the overall dominance of upregulated genes coding for ribosomal biogenesis and RNA-binding (Fig. 3A,B), we asked which transcription factors may be steering transcription toward these gene sets. To answer this, we first ran an epigenetic Landscape In Silico deletion Analysis (Lisa) (Qin et al, 2020) on all upregulated genes across the 24-h time course (Dataset EV2). We previously validated the accuracy of this computational approach for *Myc* in skeletal muscle (Jones et al, 2022; Murach et al, 2022). The five top transcription factors influencing the totality of the 24-h recovery period were *NEFLA*, *BCL3*, *FOS*, *MYC*, and *ATF3* (Fig. 5A). Since ribosome-related gene expression primarily dominated late-stage recovery at 8 and 24 h (Fig. 3D,E,G,H), we modeled which transcription factors were controlling expression of DEGs upregulated at the later time points of recovery. The influence of *MYC* on transcription coincided with the transcriptome shift towards the ribosome (Appendix Fig. S1). *MYC* was the number one transcription factor for genes expressed in the later stages of recovery—that is, genes exclusively expressed at 8 and 24 h relative to Pre (Fig. 5B).

**Figure EV5:**
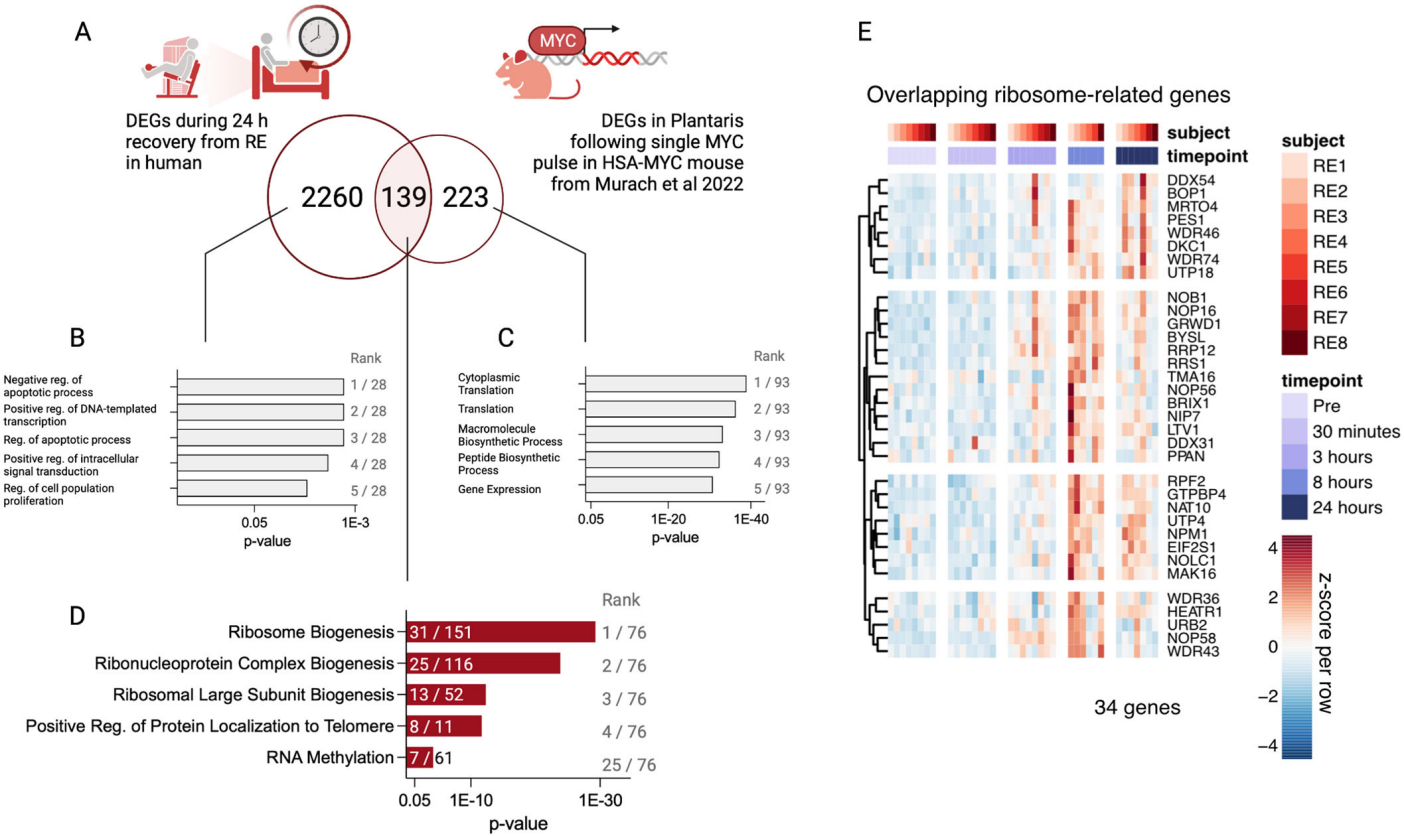
Transcriptional similarities between human RE recovery and MYC overexpression in mouse plantaris muscle. (**A**) Comparison of upregulated DEGs across 24 h of RE recovery in humans (*n* = 8) vs plantaris muscle from MYC-overexpressing mice from Murach et al (2022). (**B**–**D**) Top gene sets (GO: Biological processes) based on DEGs in (**B**) the human exclusive gene list, (**C**) MYC mouse exclusive gene list, and (**D**) overlapping gene list, respectively. (**B**–**D**) Gene ontology (GO) gene set enrichment analysis is analyzed using a Fisher exact test with Benjamini–Hochberg *p*-value correction. Gene sets are ranked according to their adj. *p*-values. (**G**) Heatmap showing DEG pattern for ribosome-related genes overlapping human RE response to a MYC response in mouse plantaris muscle. Source data are available online for this figure.

Next, we compared the human 24-h post-exercise transcriptional landscape to our previously published datasets on MYC overexpression in muscle of mice (Jones et al, 2022; Murach et al, 2022). Briefly, for these experiments, we generated a doxycycline-inducible muscle-specific model of pulsed MYC induction, called HSA-MYC (human skeletal actin reverse tetracycline transactivator tetracycline response element “tet-on” MYC) (Jones et al, 2022; Murach et al, 2022). Twelve hours of doxycycline in drinking water, followed by 12 h of non-supplemented water, causes upregulation of MYC protein in skeletal muscle (Jones et al, 2022). MYC protein returns to baseline levels after 24 h of drinking un-supplemented water (Appendix Fig. S2). We profiled the transcriptome in the plantaris and soleus muscles 12 h after doxycycline administration (Jones et al, 2022; Murach et al, 2022). Of the 2399 upregulated DEGs induced by RE over 24 h, 316 upregulated genes overlapped the response elicited in the mouse soleus muscle by a single MYC pulse (Fig. 5D–F, Dataset EV4; Jones et al, 2022). Removing the overlapping genes from the human RE response subsequently steered the transcriptional landscape away from the ribosome, as indicated by gene set enrichment analysis (GO: biological processes) on the remaining 2083 DEGs (Fig. 5D). Consequently, using the same gene set enrichment analysis on the 316 genes overlapping the human RE response and soleus transcriptome from the MYC overexpression data generated gene sets largely related to the ribosome (Fig. 5F–H)—specifically, genes coding proteins involved in ribosome biogenesis, assembly, and translation initiation and elongation (e.g., *EEF* and *EIF* genes). The gene expression time course of ribosome biogenesis-related genes under the influence of MYC largely reflected the time point-specific Lisa analysis, suggesting MYC’s influence is greatest at 8 h of recovery (Fig. 5G,H). The 672 DEGs exclusive to the MYC induction mouse mainly regulated genes associated with acute changes to transcriptional and translational speed (Fig. 5E). Regulation of overlapping genes was also evident, albeit to a lesser extent, when comparing the human RE response to the smaller MYC-mediated transcriptional response in the plantaris muscle (Fig. EV5; Murach et al, 2022).

Beyond regulation of the ribosome, other genes upregulated by both MYC induction and RE (both with adj. *p* < 0.05) included those involved in actin folding by CCT/TriC (*CCT2*, *CCT3*, *CCT4, CCT5*, *CCT6A*, *CCT7*, *CCT8*, *TCP1*), a chaperonin complex that controls sarcomere assembly and organization in striated muscle (Berger et al, 2018; Melkani et al, 2017). Genes associated with metabolism of nucleotides (*AMPD2*, *AK6*, *GART*, *IMPDH1*, *IMPDH2*, *NME1*, *NME2*, *PPAT*, *UCK2*), autophagy (*ATG3*, *HSF1*, *HSPA8*, *HSP90AA1*, *PGAM5*, *TOMM5*, *TOMM22*, *TOMM40*), translation initiation (*EIF1AD*, *EIF2S1*, *EIF2S2*, *EIF3B*, *EIF4A1*, *EIF4A3*, and *EIF5B*), as well as RNA helicases (*DDX21*, *DDX24*, *DDX31*, *DDX54*, *DDX56*, *DHX15*, *DHX30*, and *DHX33*) were also upregulated (Dataset EV4).

Downregulated genes shared by RE in humans and MYC induction in mice included *DNMT3A*, a regulator of DNA methylation in skeletal muscle (Small et al, 2021; Villivalam et al, 2021), and genes involved in ErbB signaling (*CAMK2G*, *CDKN1B*, *ERBB3*, *GAB1*) (Dataset EV4). Taken together, these data suggest that MYC induction by acute RE in healthy untrained humans may influence the muscle transcriptome in part by directing transcriptional machinery toward the formation of new ribosomes and enhanced translation. MYC may also regulate several other processes involved in skeletal muscle exercise adaptation including actin folding, autophagy, and DNA methylation.

### Pulsed muscle fiber specific MYC induction in mice is sufficient for soleus muscle hypertrophy

Our data so far suggests that MYC is a major transcriptional regulator during the acute recovery from RE in human skeletal muscle. An association between skeletal muscle hypertrophy and MYC-controlled acute exercise responses such as enhanced ribosome biogenesis is established (Figueiredo et al, 2021; Hammarström et al, 2020; Stec et al, 2016; von Walden et al, 2012; West et al, 2016), and inhibiting MYC in myotubes blunts ribosome biogenesis and protein synthesis (West et al, 2016). Still, it is unclear whether repeated MYC stimuli alone are sufficient to induce hypertrophy. To address this, we utilized our murine doxycycline-inducible muscle-specific model of pulsatile MYC overexpression: HSA-MYC (Jones et al, 2022; Murach et al, 2022).

**Figure 6:**
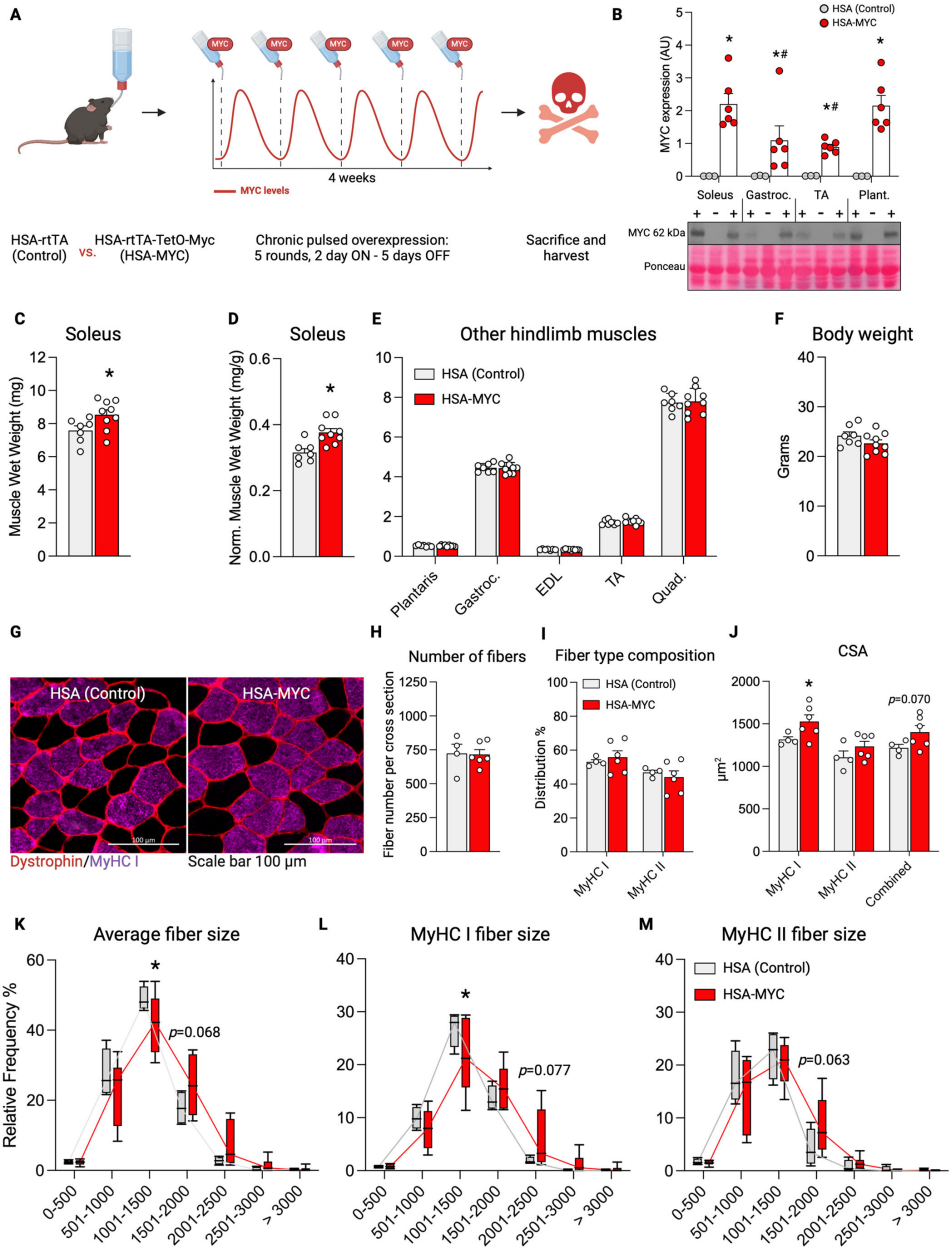
Four weeks of pulsed MYC induction is sufficient to elicit muscle fiber type specific hypertrophy. (**A**) Graphical representation of the experimental design. (**B**) Soleus (*p* = 7.1E−6 vs Con), Gastrocnemius (Gastroc; *p* = 0.0136 vs Con, *p* = 0.0254 vs Soleus), Tibialis anterior (TA; *p* = 0.0422 vs Con, *p* = 0.0048 vs Soleus), and Plantaris (Plant; *p* = 1.0E−5 vs Con) muscle probed for MYC after 48 h of doxycycline administration. + = HSA-MYC (*n* = 6), − = HSA Control (*n* = 3). (**C**) Soleus muscle weight of HSA Control and HSA MYC mice after 5 bolus exposures over 4 weeks (*p* = 0.0200 vs Con). (**D**) Soleus muscle wet weight normalized to body weight (*p* = 0.0014 vs Con). (**E**) Hindlimb muscle weight of plantaris, Gastroc, extensor digitorum longus (EDL), TA, and quadriceps (Quad.) normalized to body weight. (**F**) Body weight of mice. (**C**–**F**) *n* = 7 HSA Control, *n* = 9 HSA-MYC. (**G**) Representative images of MyHC I (purple) and MyHC II (black) muscle fiber size in HSA Control (left) and HSA-MYC (right) mice. Dystrophin is outlining fiber borders (red). Scale bar is 100 μm. (**H**) Total number of fibers per soleus muscle. (**I**) Distribution of MyHC I and MyHC II fibers expressed as a percentage. (**J**) Cross sectional area of MyHC I (*p* = 0.0433 vs Con) and MyHC II fibers, and their combined average. (**K**–**M**) Frequency distribution plot for average CSA (**K**; *p* = 0.0486 at 1001–1500 μm range vs Con) and fiber type size of MyHC I (**L**; *p* = 0.0171 at 1001–1500 μm range vs Con) and MyHC II (**M**) fibers. (**G**–**M**) *n* = 4 HSA Control, *n* = 6 HSA-MYC. Dots are biological replicates. (**B**–**F**, **H**–**J**) Values represent mean ± SEM. (**K**–**M**) The box represents the 25th–75th percentile, the line represents the median, and the whiskers represent Min to Max. *p < 0.05 vs control, ^#^*p* < 0.05 vs soleus. Analyzed using Two-way ANOVA with Bonferroni posthoc (**B**) or Fisher’s LSD (**K**–**M**), Welch’s T-test (**C**–**J**). MyHC myosin heavy chain, CSA cross sectional area. Source data are available online for this figure.

We provided doxycycline-supplemented drinking water to 4-month-old female HSA-MYC mice for 48 h, followed by 5 days of un-supplemented water for 4 weeks (five total MYC treatments in *n* = 9 animals). Doxycycline-treated littermate HSA mice were controls (*n* = 7 animals) (Fig. 6A). The doxycycline treatment strategy is similar to the approach from the Belmonte laboratory for overexpressing Yamanaka factors specifically in muscle fibers (Wang et al, 2021). The doxycycline treatment caused MYC to be significantly induced in muscle specifically (Fig. 6B; Appendix Figs. S2 and S3). The 48-h pulse strategy induced a similar amount of MYC protein in the soleus and plantaris muscles and a weaker induction in the gastrocnemius and tibialis anterior muscles; however, the induction was significant across all muscles. The administration pattern was chosen to approximate MYC induction in skeletal muscle by a regular weekly RE regimen.

Pulsed MYC induction resulted in a larger absolute mass (+12.5%, *p* = 0.002; Fig. 6B) and normalized mass (+20.7%, *p* = 0.025; Fig. 6C) of the soleus muscle relative to controls. This magnitude of soleus muscle growth is similar to what is observed after 4 weeks of progressive weighted wheel running (Englund et al, 2020) or 3 weeks of testosterone administration (Englund et al, 2019) in adult female mice. The murine soleus muscle contains a myosin heavy chain (MyHC) fiber type distribution similar to young healthy human vastus lateralis muscle (~50% MyHC I and ~50% MyHC IIa) (Bloemberg and Quadrilatero, 2012; Jones et al, 2022; Murach et al, 2020), which is the muscle from which biopsies were obtained for the current study. The mass of other predominantly fast-twitch mouse hindlimb muscles (containing MyHC 2B and 2X, as well as 2A) was not different with MYC induction versus controls (*p* > 0.05) (Fig. 6E). Likewise, the body weight of the mice was not different between groups (*p* = 0.49, Fig. 6F), nor was food intake in a subset of mice. These data collectively suggest a muscle and/or fiber-type-dependent effect of MYC for inducing muscle hypertrophy.

To further interrogate this muscle-specific growth, we performed immunohistochemistry on soleus muscle (Fig. 6G). There were no changes in the total amount of fibers within the soleus (Fig. 6H) after pulsatile MYC induction nor were there major shifts in muscle fiber type distribution (Fig. 6I). Overall (+15.1%, *p* = 0.069) and MyHC I fiber cross sectional area (+16.1%, *p* = 0.043) was larger with pulsatile MYC induction relative to controls (Fig. 6J). There was a rightward shift in overall (Fig. 6K) and MyHC I fiber size (Fig. 6L). Fibers expressing MyHC II had a more modest response to pulsatile MYC induction, showing +11.6% difference and a less pronounced rightward shift (*p* = 0.22; Fig. 6J,M). Our prior work showed that the global transcriptional response to a single pulse of MYC is most prominent in the soleus (~1400 DEGs) relative to the plantaris (~500 DEGs) and the quadriceps (<50 DEGs) (Jones et al, 2022; Murach et al, 2022). Given the western blot data presented above across muscle groups, we infer that the soleus muscle is more sensitive to MYC induction than other muscles, specifically the plantaris. These differences in gene expression between muscles likely contributed to soleus-specific mass gains. Given the fiber type and/or muscle-specific effects of *Myc* induction seen in the current and previous work, we revisited our human time course data (Figs. 1 and 2), asking if the degree of *MYC* expression could be related to fiber type distribution. However, no such indications were found, with peak *MYC* expression at 3 and 8 h (Fig. 2C) showing correlations of *r* = 0.37 (*p* = 0.29; Spearman) and *r* = −0.31 (*p* = 0.38; Spearman) vs type I fiber distribution, respectively. Future investigations will probe deeper into MYC dynamics across muscles in our model as well as the specific mechanism(s) by which MYC mediates growth of the soleus. Nevertheless, we provide the first evidence that MYC is sufficient for muscle hypertrophy in the predominant myosin heavy chain fiber types expressed in human skeletal muscle.

## Discussion

The 24-h time course of molecular responses to RE in human skeletal muscle revealed several fundamental aspects of hypertrophic exercise adaptation: (1) the DNA methylome response to RE at 30 min clearly associated with global gene expression at 3 h, (2) a burst of translation and transcription initiation coding transcripts occurs between 3 and 8 h, (3) global gene expression peaks at 8 h after an RE bout, (4) ribosomal-related gene expression dominates the mRNA landscape between 8 and 24 h during recovery after RE, (5) MYC is predicted as a highly influential transcription factor throughout the 24 h recovery period and plays a primary role in ribosomal and translation-related transcription between 8 and 24 h, as well as other processes such as autophagy and actin folding, and (6) periodic pulses of MYC are sufficient to drive muscle growth in the mixed fiber type soleus muscle of female mice.

Two to four hours after RE is typically considered the ideal time to detect changes in gene expression in skeletal muscle (Dickinson et al, 2018; Lavin et al, 2023; Louis et al, 2007; Raue et al, 2012; Yang et al, 2005). By contrast, our data show that the largest number of protein coding DEGs is detected 8 h into recovery. These new findings may influence the design of future RE studies that aim to evaluate gene expression and inform when single time-point biopsies should be taken to interrogate specific recovery processes (e.g., ribosome biogenesis versus MAPK gene expression versus ubiquitin and apoptotic gene expression). Using the same human muscle samples from this study, we previously reported that ribosome biogenesis peaks at 3 h after RE, recovers at 8 h, then rises again at 24 h (Figueiredo et al, 2021). The induction of ribosomal-related mRNAs between 8 and 24 h likely relates to the biphasic increase in rRNA that may support translational capacity for muscle growth (von Walden, 2019; Wen et al, 2016). To this point, our data reveal unique and sometimes multimodal or divergent patterns of gene expression across gene categories over the 24-h time course of recovery after RE. These patterns lend perspective to instances where opposite results in specific gene responses at different post-exercise time points are reported (Amar et al, 2021; Pillon et al, 2020).

Previous studies report mixed findings regarding the agreement between methylation and gene expression in skeletal muscle with exercise training in humans; the relationship may be weak (Robinson et al, 2017) or relatively strong (Lindholm et al, 2014; Seaborne et al, 2018b). With acute exercise, however, the acute methylation response may predict mRNA levels when specifically evaluating the promoter of exercise-responsive genes (Barres et al, 2012; Turner et al, 2019). Using a time course approach with high temporal resolution and a novel and holistic -omic integration technique, we show a strong relationship between the 30-min post-exercise global methylome response and the 3-h post-exercise transcriptome of upregulated genes in skeletal muscle from recreationally active but untrained individuals. This relationship may change with additional structured training, however. For instance, the *Myc* response to repeated bouts of RE tends to become blunted over time (Viggars et al, 2022a); this may contribute to hypertrophic response heterogeneity between individuals (Lavin et al, 2021; Phillips et al, 2013; Sparks, 2017; Stec et al, 2016). BETA analysis predicted MYC transcription to be regulated at the epigenetic level, consistent with work in cancer cells (Cheah et al, 1984; de Souza et al, 2013; Kaneko et al, 1985; Rao et al, 1989; Tsujiuchi et al, 1999). Our current and previous findings suggest that *Myc* is regulated by DNA methylation status in muscle fibers during hypertrophy (von Walden et al, 2020). More work is needed to determine whether epigenetic changes underpin lower transcriptional sensitivity of *Myc* with repeated bouts (Viggars et al, 2022a), as well as reduced training responsiveness between individuals (Stec et al, 2016). Another possibility is that the timing of transcriptional responses to acute exercise in the trained state experiences a “phase shift” relative to untrained muscle, and that this is related to “priming” by altered DNA methylation. Such a phenomenon was recently described in mouse muscle after endurance exercise training (Furrer et al, 2023). There is a need for time course studies in humans evaluating the molecular responses to RE in the trained versus untrained state so that correct interpretations can be made regarding differential expression of genes (such as *MYC*) versus differential timing of expression. Regardless, our current findings reinforce the evidence for acute exercise responses operating under the control of early methylation events (Barres et al, 2012; Seaborne et al, 2018a) and support data suggesting that methylation changes are central to exercise training adaptations in humans (Egan and Sharples, 2022; Roberts et al, 2023; Sharples and Seaborne, 2019; Turner et al, 2019).

Our work points to MYC as a key player in controlling hypertrophic adaptation to exercise in skeletal muscle. In mice, *Myc* is actively transcribed and enriched in myonuclei during mechanical overload (Murach et al, 2022). A single pulse of *Myc* in skeletal muscle markedly alters the transcriptome (soleus>plantaris>quadriceps) (Jones et al, 2022; Murach et al, 2022) and rewires the DNA methylation landscape (Jones et al, 2022). In humans, MYC may act directly on rDNA after RE to influence ribosome biogenesis (Figueiredo et al, 2021), consistent with MYC’s occupation of the rDNA promoter during load-induced muscle hypertrophy in mice (von Walden et al, 2012). MYC controls ribosome biogenesis as well as skeletal muscle protein synthesis independent from mTORC1 activation (Mori et al, 2020; West et al, 2016), but its ability to drive muscle growth has been unclear (Mori et al, 2020; Phillips et al, 2013). It is likely that chronic induction of MYC in muscle is detrimental to mass and function, similar to what occurs with prolonged chronic mTORC1 activation (Castets et al, 2013; Ham et al, 2020). By using a doxycycline-inducible and pulsatile model of MYC induction in skeletal muscle, we show for the first time that MYC can promote growth of skeletal muscle mass in the murine soleus. Our prior and current work suggests this hypertrophy could be due to ribosomal regulation—biogenesis, efficiency, and/or specialization (Jones et al, 2022; Murach et al, 2022). Larger muscle size caused by MYC may also be attributable to the regulation of several other processes such as actin folding and/or autophagy among others. Transcriptome data from MYC induction in the soleus indicates that a variety of other processes contribute to hypertrophy since >1300 genes are altered by a single MYC pulse (Jones et al, 2022). Our findings encourage further investigation of how pulsatile MYC supports long-term anabolism at the molecular, signaling, and cellular level across muscles, fiber types, sexes, and ages.

Collectively, the time course of -omics responses to RE in healthy untrained humans, alongside the repeated biopsy control group, is a valuable resource to the skeletal muscle field. Our results define the molecular landscape after exercise at high temporal resolution and will help inform the design of future human exercise studies. We detail the interplay between the methylome and transcriptome, identify MYC as a key component of the RE response, and show that MYC is sufficient for muscle growth. This study complements previous and ongoing efforts at defining the acute muscle -omics responses to RE in humans (Amar et al, 2021; Pillon et al, 2020; Sanford et al, 2020) to uncover new molecular regulators of hypertrophic physical activity. We lay the groundwork for future investigations that will expand on how transcriptional regulators such as *MYC* control muscle mass and adaptation in skeletal muscle.

## Methods

**Table:** Reagents and tools table

Reagent/Resource	Reference or Source	Identifier or Catalog Number
**Experimental Models**		
Human skeletal actin reverse tetracycline transactivator tetracycline response element “tet-on” HSA-MYC Mice	Jackson Laboratory	Strains 038301 & 019736
**Recombinant DNA**		
**Antibodies**		
Dystrophin primary antibody	Abcam, St. Louis, MO, USA	ab15277
MyHC 1	Developmental Studies Hybridoma Bank, Iowa City, IA, USA	BA-D5
Anti-MYC	Cell Signaling, Danvers, MA, USA	D84C12 cat. 5605
Secondary antibody utilized for MYC stain	LI-COR Biosciences, Lincoln, NE	IRDye 800CW/680RD
**Oligonucleotides and other sequence-based reagents**		
**Chemicals, Enzymes and other reagents**		
TRI Reagent	Sigma-Aldrich, St Louis, MO, USA	
Doxycycline hyclate	Sigma	D9891-5G
**Software**		
MyoVision	Viggars et al, 2022b; Wen et al, 2017	
HISAT2 (2.0.5)	Kim et al, 2015	
featureCounts (1.5.0-p3)	Liao et al, 2014	
R (Version: 4.1.0)	https://cran.r-project.org/bin/windows/base/old/4.1.0/	
DESeq2 (1.42.0)	Love et al, 2014	
org.Hs.eg.db: Genome wide annotation for Human. R package (version 3.8.2)	Carlson et al, 2019	
CIBERSORTx	Newman et al, 2019	
Seurat analysis pipeline	Hao et al, 2021	
methylKit R package	Akalin et al, 2012	
Enrichr with 2023 gene ontology database	https://maayanlab.cloud/Enrichr/ 2023-Aug-16 Aleksander et al, 2023; Chen et al, 2013; Kuleshov et al, 2016; Xie et al, 2021	
Landscape In Silico deletion (LISA) Analysis	Qin et al, 2020	
Venny (2.1)	https://bioinfogp.cnb.csic.es/tools/venny/index.html 2023-Aug-23	
BETA Analysis	Wang et al, 2013	
GraphPad Prism (version 7.00 for Mac OS X)	GraphPad Software, La Jolla, CA	
Rstudio	https://posit.co/download/rstudio-desktop/	
BioRender	https://www.biorender.com/	
Affinity Designer (2.3)	https://affinity.serif.com/en-us/designer/	
**Other**		
Illumina NovaSeq 6000	150 bp paired-end sequencing; Novogene Corp. Inc., Sacramento, CA, USA	
Single-cell RNA sequencing data from Lovrić et al (GSE214544)	Lovrić et al, 2022	
Previously published reduced representation bisulfite sequencing (RRBS) for ribosomal DNA (rDNA)	Figueiredo et al, 2021	
Trim Galore Wrapper (FastQC & Cutadapt)	https://github.com/FelixKrueger/TrimGalore	
Published gene- and fiber-type area distribution data	Reitzner et al, 2024	
RC/DC Protein Assay	BioRad, Hercules, CA, USA	cat. 500-0119

### Ethical approval

The regional Ethical Review Board in Linköping (2017/183-31) approved the study protocol for the human intervention. The volunteers received oral and written information about the study, and subsequently provided their informed consent prior to study enrollment. The study protocol conformed with the Declaration of Helsinki. IACUCs at the University of Arkansas (UA, AUP 21038) approved all animal procedures. Mice were housed in a temperature and humidity-controlled room, maintained on a 12:12 h light:dark cycles, and food and water were provided ad libitum throughout experimentation. All animals were sacrificed via cervical dislocation under deep anesthesia with inhaled isoflurane.

### Volunteers

A subset of thirteen volunteers was chosen for analysis from previously published studies (Figueiredo et al, 2021; von Walden et al, 2021). The subset was chosen based on which participants had the most complete set of biopsy materials still available. Eight recreationally active volunteers were analyzed from the RE group (5m/3f), and five from the CON group (3m/2f). The volunteers in the RE group had a mean age of 32 ± 5 years, height of 181 ± 9 cm, weight of 83 ± 8 kg, and body mass index (BMI) of 25.3 ± 2.0. The corresponding values for the CON group were an age of 30 ± 4 years, height of 177 ± 5 cm, weight of 85 ± 12 kg, and a BMI of 27.3 ± 3.6.

### Experimental protocol

The experimental protocol has been described elsewhere (Figueiredo et al, 2021; von Walden et al, 2021). In short, volunteers were instructed to not partake in any strenuous physical activity for the legs for 3 days prior to the intervention. Following an overnight fast, subjects consumed a breakfast consisting of a standardized amount of liquid formula supplying 1.05/0.28/0.25 grams of carbohydrates/protein/fat per kg of body weight (Resource Komplett Näring, Nestlé Health Science, Stockholm, Sweden). Skeletal muscle biopsies were collected from the vastus lateralis, using a Bergström needle with manually applied suction (Evans et al, 1982). Ninety minutes after breakfast, volunteers started a 45-min standardized RE session. The RE session consisted of a short warm-up on a cycle ergometer, followed by four sets at 7RM load with two min of rest using both leg press and leg extension machines. Muscle biopsies were collected 1 h after breakfast (Pre), as well as 30 min and 3 h after RE completion. Between the completion of the exercise and the 3-h biopsy, volunteers were resting in a seated position under the supervision of the test leaders. Immediately following the 3-h biopsy, another portion of the standardized liquid formula was administered for lunch to the volunteers (2.1/0.56/0.5 grams of carbohydrates/protein/fat per kg of body weight). Following the standardized lunch, volunteers were allowed to go home but were instructed to refrain from physical activity and food intake. Eight hours after RE ended, volunteers reported to the laboratory again, and another muscle biopsy was collected whereby the volunteers were sent home overnight. At home, volunteers were instructed to eat a standard dinner (a balanced meal of ~25% of a protein source and equal distribution of carbohydrate sources and greens) in the evening. Volunteers were again instructed to refrain from any physical activity other than light work. Volunteers got clear instructions to follow but were not monitored during the rest period between the 3-h and 8-h, as well as the 8-h and 24-h sampling. Another administered standardized liquid formula breakfast was ingested the following morning 90 min prior to reporting to the laboratory for the final muscle biopsy sampling 24 h after RE completion (breakfast ingested 2 h prior to biopsy sampling). The experimental protocol is depicted in Fig. 1A (CON) and Fig. 2A (RE). The sampling time points for the CON group was matched to the exercise group.

### RNA extraction, sequencing, and analysis

Approximately 25 mg of muscle tissue was homogenized in TRI Reagent (Sigma-Aldrich, St Louis, MO, USA) using a Bullet Blender Tissue Homogenizer (Next Advance, Troy, NY, USA). An RNA supernatant phase was then isolated using bromochloropropane and centrifugation. Next, the RNA phase was processed using Direct-zol filter columns (Zymo Research, Irvine, CA, USA). Finally, the RNA was treated with DNAse and eluted in DEPC-treated water prior to storage at −80 °C. Concentration, and purity of the RNA was determined using a BioTek PowerWave XS microplate reader (BioTek Instruments Inc., Winooski, VT, USA). Library preparation of mRNA was done using Poly A enrichment, followed by RNA sequencing by an Illumina NovaSeq 6000 (150 bp paired-end sequencing; Novogene Corp. Inc., Sacramento, CA, USA).

Quality control of raw sequencing reads was performed by removing adapters and low-quality reads. Subsequently, the reads were aligned to the human reference genome (GRCh38.p12) using HISAT2 (2.0.5) (Kim et al, 2015), and the quantification of reads mapped to each gene was conducted using featureCounts (1.5.0-p3) (Liao et al, 2014). Raw counts were used as inputs for the downstream analysis in R platform (Version: 4.1.0). After filtering out genes with low expression, DESeq2 (1.42.0) was used for the normalization and differential analyses in the comparison between different time point groups (Love et al, 2014). Genes with a false discovery rate (Benjamini–Hochberg method) adjusted *p*-value < 0.05 were identified as differentially expressed genes (DEGs). We have used org.Hs.eg.db (3.13.0) as reference for the annotation of human genes (org.Hs.eg.db: Genome wide annotation for Human. R package version 3.8.2.) (Carlson et al, 2019). To determine gene expression patterns among the DEGs, we computed z-score per gene along different time points for each group separately and employed the Euclidean hierarchical clustering method to identify clustered genes. The total number of clusters was determined empirically. Raw and processed files have been deposited in the GEO database (GSE252357).

### Data deconvolution

Leveraging single-cell RNA sequencing data, the relative abundance of different cell types from bulk tissue transcriptomes was estimated using the computational tool CIBERSORTx (Newman et al, 2019). Single-cell RNA sequencing data from Lovrić et al (GSE214544) (Lovrić et al, 2022) were used for constructing the reference matrix of the analyses. The original datasets based on 10X Genomics technology, were reanalyzed using the Seurat pipeline (Hao et al, 2021). Different cell types were separated with the resolution parameter set to 0.5 and then annotated based on marker genes from the previous publication (Hao et al, 2021). For determination of mononuclear cells, cell populations annotated as “myocytes” were excluded from the analyses as they dominate the transcriptome in the deconvolution. Subsequently, the normalized gene expression data of individual cells were compiled to create a comprehensive signature matrix encompassing the entire spectrum of different cell types. This matrix served as the basis of cell type proportion prediction in CIBERSORTx, with 1000 permutations to ensure the robustness and accuracy of the predictions.

### DNA methylation data processing and statistical analysis

We previously published reduced representation bisulfite sequencing (RRBS) for ribosomal DNA (rDNA) (Figueiredo et al, 2021). Here, we used this RRBS dataset for global DNA methylation analysis (von Walden et al, 2020). Quality control and adapter sequence trimming were performed using FastQC and Cutadapt, respectively as parts of the Trim Galore wrapper. Low-quality base calls (Phred score <20) were removed prior to trimming adapter sequences. Bismark aligner was used to align the sequence reads to the bisulfite-converted GRCh38 genome prior to data processing. Coverage (.cov) files produced from Bismark aligner were used for data analysis in the methylKit R package (Akalin et al, 2012). MethylKit was used to pool samples into their respective groups to maximize read coverage across the genome using a minimum read cutoff of 10 reads per base, and minimum base coverage of 1 sample per group. Differentially methylated regions (DMRs) were determined by genomic ranges for every gene promoter as defined by the hg38.bed file obtained from NCBI. Fisher’s exact test with sliding linear model (SLIM) correction for false discovery (Wang et al, 2011) was used to qualify both differentially methylated sites and differentially methylated promoters within the dataset. Percent methylation and percent differential methylation were then obtained from methylKit following analysis.

### Pathway analysis, transcriptional regulators, and BETA analysis

The up- and downregulated DEG (adj. *p* < 0.05) were initially separated. All DEGs from 30-min, 3-, 8-, and 24-h post-exercise biopsies were collapsed into one DEG list across the 24-h recovery period for up- and downregulated genes, respectively. Gene set enrichment analyses were conducted on the collapsed gene lists using Enrichr (https://maayanlab.cloud/Enrichr/ 2023-Aug-16) with the 2023 gene ontology (GO) database as our cross reference (Aleksander et al, 2023; Chen et al, 2013; Kuleshov et al, 2016; Xie et al, 2021). We used all protein-coding genes detected in our muscle samples (14,392 genes, Figs. EV1 and 2) as our background correction for the pathway analysis, as suggested by Stokes et al (Stokes et al, 2023). The output for gene sets within the Biological Process and Molecular Function of the Gene Ontology database are reported as source data, and the number of genes, and adjusted *p*-values of selected enriched gene sets are presented in Fig. 2. The time course analysis for each gene set is based on the proportion of DEGs within each gene set in the current dataset expressed at each biopsy time point relative to Pre. Thus, the number of DEGs within a specific gene set per time point is divided by the number of DEGs within the same gene set from the pooled 24-h DEG list, described above.

Landscape In Silico deletion Analysis (Lisa) was run according to the recommended procedures as reported by Qin et al (Qin et al, 2020), consistent with our previous work (Jones et al, 2022; Murach et al, 2022). In brief, DEG lists (adj. *p* < 0.05) were run using software on http://lisa.cistrome.org (2023-Aug-20). If the number of DEGs was above 500, the analysis was run locally using the command line. Lisa analysis was performed on the collapsed upregulated 24-h gene list, and on upregulated genes expressed at different time points. The Cauchy combination *p*-value test was used to determine the overall influence of MYC. Overlapping DEGs between the human RE response and the MYC mouse was analyzed using Venny2.1 (https://bioinfogp.cnb.csic.es/tools/venny/index.html 2023-Aug-23).

We presented the method for incorporation of RRBS and RNA-seq data using BETA in a previous publication (Ismaeel et al, 2023). BETA is a software that provides an integrated analysis of transcription factor binding to genomic DNA and transcript abundance using chromatin immunoprecipitation sequencing (ChIP-seq) and transcriptomics (RNA-seq) datasets (Wang et al, 2013). BETA takes into consideration the distance of the regulatory element relative to the transcription start site (TSS) by modeling the effect of regulation using a natural log function termed the regulatory potential (Eq. 1), as described previously by Tang and colleagues (Tang et al, 2011). CpG islands were converted into “methylation peaks” similar to transcription factor binding peaks, which is built using the GRCm39 CpG island bedfile downloaded from the UCSC genome browser. Only genes differentially expressed with adjusted *p* < 0.05 from RNA sequencing analysis were included as input for gene expression. The BETA basic command was run with the following parameters “-c 0.05 --df 0.05 --da 500”.

1 $${s}_{g}={\sum }_{i=1}^{k}{e}^{-(0.5+4{\Delta }_{i})}$$

In the current study, a CpG island (*k*) within 100 kb of TSS of gene (*g*) is included in the calculation for the regulatory potential score (*s*). The distance between the CpG island and the TSS is expressed as a ratio relative to 100 kb (∆). The scoring is weighted based on the distance from the TSS (higher for smaller distances, lower for larger distances).

### BETA integration gene set enrichment analysis

Gene set analysis was performed using the enrichR R package. Up- and down-target genes resulting from BETA integration of DNA methylation and RNA-sequencing datasets were included in independent up and down gene sets. The 2023 Gene Ontology database Biological Processes was used to annotate up and down gene sets and determine enriched ontologies for each gene set. GOplot R package was used to combine log2 fold change for each gene with their respective enriched gene sets.

### Interpolation of fiber type distribution in muscle samples

Published gene- and fiber-type area distribution data from Reitzner et al (Reitzner et al, 2024) were obtained. A ratio of *MYH7* to *MYH1* & *MYH2* normalized counts was created for each baseline sample (*MYH7*/(*MYH1* + *MYH2*)). Next, a standard curve was created for estimating fiber type distributions based on the gene ratio (Fig. EV4A). An identical gene ratio (*MYH7*/(*MYH1* + MYH2)) was created for the baseline samples in our dataset, and type I fiber area % was subsequently interpolated using the standard curve created from the values obtained from Reitzner et al (Reitzner et al, 2024).

### Generation of HSA-MYC mice and in vivo pulsatile overexpression experiments

Human skeletal actin reverse tetracycline transactivator tetracycline response element “tet-on” MYC (HSA-MYC) mice were generated as previously described (Jones et al, 2022; Murach et al, 2022) (Jackson Laboratory Strains 038301 and 019736). A subset of mice (*n* = 8) was crossed with tet-on green fluorescent protein mice for myonuclear isolation experiments not presented here (Jackson Laboratory Strain 005104). For all MYC experiments, littermate mice (HSA or HSA-GFP) were controls; all mice were heterozygous for each transgene. At four months of age, control (*n* = 7) and MYC overexpressing female mice (*n* = 9) were given doxycycline in drinking water with sucrose (0.5 mg mL^–1^ with 2% sucrose) for 48 h. All mice were then given un-supplemented drinking water for the remaining 5 days of the week. This dosing strategy was repeated 5 total times. All mice were euthanized 24 h following the final doxycycline treatment. Some mice were used for analyses not described here, so the histology results are from *n* = 4 control and *n* = 6 MYC induction mice. Mice were euthanized in the morning (before 10:00 AM) and all tissues were harvested, weighed, and frozen in liquid nitrogen-cooled isopentane using optimal cutting temperature compound. The average mass of both muscles for every mouse is presented.

### Immunohistochemistry

Fiber cross sectional area and fiber type analyses on the soleus muscles were performed as previously described (Dungan et al, 2022; Murach et al, 2020). Briefly, 8 µm sections were cut using a cryostat and air dried for ≥1 h. Primary antibodies for dystrophin (1:100, ab15277, Abcam, St. Louis, MO, USA) and MyHC 1 (BA-D5, Developmental Studies Hybridoma Bank, Iowa City, IA, USA) were applied for ≥4 h in a PBS cocktail. After several PBS washes, isotype-specific secondary antibodies were applied for 1 h. Following several PBS washes, the slides were mounted with cover slips using a 50/50 solution of PBS and glycerol. Muscle cross sections were imaged using a Zeiss AxioImager M2. Fiber cross sectional area, fiber number, and fiber type distribution was analyzed using MyoVision (Viggars et al, 2022b; Wen et al, 2017), as we have previously described, using the entire muscle cross-section.

### Western blotting for MYC

Western blots were for MYC were carried out as previously described (Jones et al, 2022). Briefly, 20 mg of frozen muscles and liver were powdered and homogenized in Laemmli buffer. Following RC/DC assay (BioRad, Hercules, CA, USA, cat. 500-0119), 40 μg of total protein was subjected to SDS-PAGE using a 10% gel. Membranes were blocked with 5% of milk. Primary antibody incubation was conducted at 4 °C for ~72 hours using anti-MYC (D84C12 cat. 5605, Cell Signaling, Danvers, MA, USA) diluted 1:500. Secondary antibody (IRDye 800CW/680RD, LI-COR Biosciences, Lincoln, NE) was diluted 1:10,000 and membranes were imaged on LI-COR Odyssey FC using IR detection. All bands were normalized to the 45-kDa actin band of Ponceau S stain as a loading control.

### Statistical considerations

Figures were generated using GraphPad Prism version 7.00 for Mac OS X (GraphPad Software, La Jolla, CA), Rstudio, BioRender, and Affinity Designer 2.3. Data presented as mean ± standard deviation of mean unless otherwise stated. For the human study, sample sizes were based on tissue availability from a prior investigation where the most complete sample sets were used (Figueiredo et al, 2021). For the murine studies, sample size was based on mouse availability, but the magnitude of increase in soleus mass (~15%) that we previously observed with 4 weeks of murine exercise (*n* = 8/group) (Englund et al, 2020) or 3 weeks of testosterone administration (*n* = 6–8) (Englund et al, 2019) was also used to inform our analysis. Average muscle weights for each animal (mean of both muscles) and histology data were analyzed using two-tailed dependent t-tests with *p* < 0.05. Prior to t-tests, data were analyzed for normality using Shapiro–Wilks formula. For all -omics analyses, Benjamini–Hochberg adjusted *p* values (adj. *p* < 0.05) were utilized.

## Supplementary information

Appendix

Peer Review File

Dataset EV1

Dataset EV2

Dataset EV3

Dataset EV4

Source data Fig. 1

Source data Fig. 3

Source data Fig. 4

Source data Fig. 5

Source data Fig. 6

Appendix and EV Figure Source Data

Expanded View Figures

## Data Availability

RNA-sequencing data were deposited in GEO under accession GSE252357; processed data are provided as source data. A publicly available user-friendly web-based application is provided at http://data.myoanalytics.com for browsing the human transcriptional time course data. RRBS was deposited in association with our previous publication (Figueiredo et al, 2021). RRBS data are in GEO: GSE252357. The source data of this paper are collected in the following database record: biostudies:S-SCDT-10_1038-S44319-024-00299-z.

## References

[CR1] Akalin A, Kormaksson M, Li S, Garrett-Bakelman FE, Figueroa ME, Melnick A, Mason CE (2012) methylKit: a comprehensive R package for the analysis of genome-wide DNA methylation profiles. Genome Biol 13:R87 23034086 10.1186/gb-2012-13-10-r87 PMC3491415

[CR2] Aleksander SA, Balhoff J, Carbon S, Cherry JM, Drabkin HJ, Ebert D, Feuermann M, Gaudet P, Harris NL (2023) The Gene Ontology knowledgebase in 2023. Genetics 224:iyad031 36866529 10.1093/genetics/iyad031 PMC10158837

[CR3] Alvares LE, Schubert FR, Thorpe C, Mootoosamy RC, Cheng L, Parkyn G, Lumsden A, Dietrich S (2003) Intrinsic, Hox-dependent cues determine the fate of skeletal muscle precursors. Dev Cell 5:379–390 12967558 10.1016/s1534-5807(03)00263-6

[CR4] Alway SE (1997) Overload-induced C-Myc oncoprotein is reduced in aged skeletal muscle. J Gerontol Ser A 52:B203–B211 10.1093/gerona/52a.4.b203 9224425

[CR5] Amar D, Lindholm ME, Norrbom J, Wheeler MT, Rivas MA, Ashley EA (2021) Time trajectories in the transcriptomic response to exercise-a meta-analysis. Nat Commun 12:1–12 34108459 10.1038/s41467-021-23579-x PMC8190306

[CR6] Apró W, Moberg M, Hamilton DL, Ekblom B, Rooyackers O, Holmberg HC, Blomstrand E (2015) Leucine does not affect mechanistic target of rapamycin complex 1 assembly but is required for maximal ribosomal protein s6 kinase 1 activity in human skeletal muscle following resistance exercise. FASEB J 29:4358–4373 26169935 10.1096/fj.15-273474

[CR7] Armstrong DD, Esser KA (2005) Wnt/β-catenin signaling activates growth-control genes during overload-induced skeletal muscle hypertrophy. Am J Physiol Cell Physiol 289:C853–C859 15888552 10.1152/ajpcell.00093.2005

[CR8] Barres R, Yan J, Egan B, Treebak JT, Rasmussen M, Fritz T, Caidahl K, Krook A, O’Gorman DJ, Zierath JR (2012) Acute exercise remodels promoter methylation in human skeletal muscle. Cell Metab 15:405–411 22405075 10.1016/j.cmet.2012.01.001

[CR9] Benard G, Neutzner A, Peng G, Wang C, Livak F, Youle RJ, Karbowski M (2010) IBRDC2, an IBR-type E3 ubiquitin ligase, is a regulatory factor for Bax and apoptosis activation. EMBO J 29:1458–1471 20300062 10.1038/emboj.2010.39 PMC2868578

[CR10] Berger J, Berger S, Li M, Jacoby AS, Arner A, Bavi N, Stewart AG, Currie PD (2018) In vivo function of the chaperonin TRiC in α-actin folding during sarcomere assembly. Cell Rep 22:313–322 29320728 10.1016/j.celrep.2017.12.069

[CR11] Bloemberg D, Quadrilatero J (2012) Rapid determination of myosin heavy chain expression in rat, mouse, and human skeletal muscle using multicolor immunofluorescence analysis. PLoS ONE 7:e35273 22530000 10.1371/journal.pone.0035273 PMC3329435

[CR12] Broholm C, Laye MJ, Brandt C, Vadalasetty R, Pilegaard H, Pedersen BK, Scheele C (2011) LIF is a contraction-induced myokine stimulating human myocyte proliferation. J Appl Physiol 111:251–259 21527666 10.1152/japplphysiol.01399.2010

[CR13] Brook MS, Wilkinson DJ, Mitchell WK, Lund JN, Phillips BE, Szewczyk NJ, Greenhaff PL, Smith K, Atherton PJ (2016) Synchronous deficits in cumulative muscle protein synthesis and ribosomal biogenesis underlie age-related anabolic resistance to exercise in humans. J Physiol 594:7399–7417 27654940 10.1113/JP272857 PMC5157077

[CR14] Brooks TA, Hurley LH (2010) Targeting MYC expression through G-quadruplexes. Genes Cancer 1:641–649 21113409 10.1177/1947601910377493 PMC2992328

[CR15] Carlson M, Falcon S, Pages H, Li N (2019) org. Hs. eg. db: Genome wide annotation for Human. R package version 3:3

[CR16] Castets P, Lin S, Rion N, Di Fulvio S, Romanino K, Guridi M, Frank S, Tintignac LA, Sinnreich M, Rüegg MA (2013) Sustained activation of mTORC1 in skeletal muscle inhibits constitutive and starvation-induced autophagy and causes a severe, late-onset myopathy. Cell Metab 17:731–744 23602450 10.1016/j.cmet.2013.03.015

[CR17] Chambers TL, Dimet-Wiley A, Keeble AR, Haghani A, Lo W-J, Kang G, Brooke R, Horvath S, Fry CS, Watowich SJ et al (2024) Methylome-proteome integration after late-life voluntary exercise training reveals regulation and target information for improved skeletal muscle health. J Physiol. 10.1113/JP286681 10.1113/JP286681 PMC11702923 39058663

[CR18] Cheah MS, Wallace CD, Hoffman RM (1984) Hypomethylation of DNA in human cancer cells: a site-specific change in the c-myc oncogene. J Nat Cancer Inst 73:1057–1065 6092764

[CR19] Chen EY, Tan CM, Kou Y, Duan Q, Wang Z, Meirelles GV, Clark NR, Ma’ayan A (2013) Enrichr: interactive and collaborative HTML5 gene list enrichment analysis tool. BMC Bioinforma 14:1–14 10.1186/1471-2105-14-128 PMC3637064 23586463

[CR20] Chen YW, Nader GA, Baar KR, Fedele MJ, Hoffman EP, Esser KA (2002) Response of rat muscle to acute resistance exercise defined by transcriptional and translational profiling. J Physiol 545:27–41 12433947 10.1113/jphysiol.2002.021220 PMC2290672

[CR21] Correia JC, Jannig PR, Gosztyla ML, Cervenka I, Ducommun S, Præstholm SM, Dumont K, Liu Z, Liang Q, Edsgärd D et al (2023) Zfp697 is an RNA-binding protein that regulates skeletal muscle inflammation and regeneration. Proc Nat Acad Sci USA 124:e2319724121 10.1073/pnas.2319724121 PMC11348326 39141348

[CR22] da Silva MT, Joshi AS, Castillo MB, Koike TE, Roy A, Gunaratne PH, Kumar A (2023) Fn14 promotes myoblast fusion during regenerative myogenesis. Life Sci Alliance 6:lsa202302312 10.26508/lsa.202302312 PMC10561765 37813488

[CR23] Dang CV (1999) c-Myc target genes involved in cell growth, apoptosis, and metabolism. Mol Cell Biol 19:1–11 9858526 10.1128/mcb.19.1.1 PMC83860

[CR24] Dang CV (2012) MYC on the path to cancer. Cell 149:22–35 22464321 10.1016/j.cell.2012.03.003 PMC3345192

[CR25] Dang CV, O’Donnell KA, Zeller KI, Nguyen T, Osthus RC, Li F (2006) The c-Myc target gene network. In: Seminars in cancer biology. Elsevier, pp 253–264 10.1016/j.semcancer.2006.07.014 16904903

[CR26] Das SK, Lewis BA, Levens D (2022) MYC: a complex problem. Trend Cell Biol. 10.1016/j.tcb.2022.1007.1006 10.1016/j.tcb.2022.07.006 PMC9911561 35963793

[CR27] de Souza CRT, Leal MF, Calcagno DQ, Costa Sozinho EK, Borges BdN, Montenegro RC, Dos Santos AKCR, Dos Santos SEB, Ribeiro HF, Assumpção PP (2013) MYC deregulation in gastric cancer and its clinicopathological implications. PLoS ONE 8:e64420 23717612 10.1371/journal.pone.0064420 PMC3661519

[CR28] Demontis F, Perrimon N (2009) Integration of Insulin receptor/Foxo signaling and dMyc activity during muscle growth regulates body size in Drosophila. Development 135:983–993 10.1242/dev.027466 PMC2727562 19211682

[CR29] Dickinson JM, D’Lugos AC, Naymik MA, Siniard AL, Wolfe AJ, Curtis DR, Huentelman MJ, Carroll CC et al (2018) Transcriptome response of human skeletal muscle to divergent exercise stimuli. J Appl Physiol 124:1529–1540 29543133 10.1152/japplphysiol.00014.2018

[CR30] Dungan CM, Brightwell C, Wen Y, Zdunek CJ, Latham C, Thomas NT, Zagzoog AM, Brightwell BD, VonLehmden GL, Keeble AR et al (2022) Muscle-specific cellular and molecular adaptations to late-life voluntary concurrent exercise. Function 3:zqac027 35774589 10.1093/function/zqac027 PMC9233305

[CR31] Egan B, O’connor PL, Zierath JR, O’gorman DJ (2013) Time course analysis reveals gene-specific transcript and protein kinetics of adaptation to short-term aerobic exercise training in human skeletal muscle. PLoS ONE 8:e74098 24069271 10.1371/journal.pone.0074098 PMC3771935

[CR32] Egan B, Sharples AP (2022) Molecular responses to acute exercise and their relevance for adaptations in skeletal muscle to exercise training. Physiol Rev 103:2057–2170 36395350 10.1152/physrev.00054.2021

[CR33] Englund D, Figueiredo V, Dungan C, Murach K, Peck B, Petrosino J, Brightwell C, Dupont A, Neal A, Fry C et al (2020) Satellite cell depletion disrupts transcriptional coordination and muscle adaptation to exercise. Function 2:zqaa033 34109314 10.1093/function/zqaa033 PMC8179974

[CR34] Englund DA, Peck BD, Murach KA, Neal AC, Caldwell HA, McCarthy JJ, Peterson CA, Dupont-Versteegden EE et al (2019) Resident muscle stem cells are not required for testosterone-induced skeletal muscle hypertrophy. Am J Physiol Cell Physiol 317:C719–C724 31314585 10.1152/ajpcell.00260.2019 PMC6851003

[CR35] Evans W, Phinney S, Young V (1982) Suction applied to a muscle biopsy maximizes sample size. Med Sci Sport Exerc 14:101–102 7070249

[CR36] Fan L, Peng G, Sahgal N, Fazli L, Gleave M, Zhang Y, Hussain A, Qi J (2016) Regulation of c-Myc expression by the histone demethylase JMJD1A is essential for prostate cancer cell growth and survival. Oncogene 35:2441–2452 26279298 10.1038/onc.2015.309 PMC4757517

[CR37] Ferreira DM, Cheng AJ, Agudelo LZ, Cervenka I, Chaillou T, Correia JC, Porsmyr-Palmertz M, Izadi M, Hansson A, Martínez-Redondo V (2019) LIM and cysteine-rich domains 1 (LMCD1) regulates skeletal muscle hypertrophy, calcium handling, and force. Skelet Muscle 9:1–19 31666122 10.1186/s13395-019-0214-1 PMC6822430

[CR38] Figueiredo VC, Roberts LA, Markworth JF, Barnett MP, Coombes JS, Raastad T, Peake JM, Cameron-Smith D (2016) Impact of resistance exercise on ribosome biogenesis is acutely regulated by post-exercise recovery strategies. Physiol Rep 4:e12670 26818586 10.14814/phy2.12670 PMC4760384

[CR39] Figueiredo VC, Wen Y, Alkner B, Fernandez-Gonzalo R, Norrbom J, Vechetti JrIJ, Valentino T, Mobley CB, Zentner GE, Peterson CA et al (2021) Genetic and epigenetic regulation of skeletal muscle ribosome biogenesis with exercise. J Physiol 599:3363–3384 33913170 10.1113/JP281244

[CR40] Furrer R, Heim B, Schmid S, Dilbaz S, Adak V, Nordström KJ, Ritz D, Steurer SA, Walter J, Handschin C (2023) Molecular control of endurance training adaptation in male mouse skeletal muscle. Nat Metab 5:2020–2035 37697056 10.1038/s42255-023-00891-y PMC10663156

[CR41] Gidlund E-k, Ydfors M, Appel S, Rundqvist H, Sundberg CJ, Norrbom J (2015) Rapidly elevated levels of PGC-1α-b protein in human skeletal muscle after exercise: exploring regulatory factors in a randomized controlled trial. J Appl Physiol 119:374–384 26089547 10.1152/japplphysiol.01000.2014

[CR42] Gustafsson T, Puntschart A, Kaijser L, Jansson E, Sundberg CJ (1999) Exercise-induced expression of angiogenesis-related transcription and growth factors in human skeletal muscle. Am J Physiol Heart Circ Physiol 276:H679–H685 10.1152/ajpheart.1999.276.2.H679 9950871

[CR43] Ham DJ, Börsch A, Lin S, Thürkauf M, Weihrauch M, Reinhard JR, Delezie J, Battilana F, Wang X, Kaiser MS et al (2020) The neuromuscular junction is a focal point of mTORC1 signaling in sarcopenia. Nat Commun 11:4510 32908143 10.1038/s41467-020-18140-1 PMC7481251

[CR44] Hammarström D, Øfsteng S, Koll L, Hanestadhaugen M, Hollan I, Apro W, Whist JE, Blomstrand E, Rønnestad BR, Ellefsen S (2020) Benefits of higher resistance-training volume are related to ribosome biogenesis. J Physiol 598:543–565 31813190 10.1113/JP278455

[CR45] Hao Y, Hao S, Andersen-Nissen E, Mauck III, WM, Zheng S, Butler A, Lee MJ, Wilk AJ, Darby C, Zager M (2021) Integrated analysis of multimodal single-cell data. Cell 184:3573–3587.e3529 34062119 10.1016/j.cell.2021.04.048 PMC8238499

[CR46] Ismaeel A, Thomas NT, McCashland M, Vechetti Jr I, Edman S, Lanner JT, Figueiredo V, Fry C, McCarthy J, Wen Y et al (2023) Coordinated regulation of myonuclear DNA methylation, mRNA, and miRNA levels associates with the metabolic response to rapid synergist ablation-induced skeletal muscle hypertrophy in female mice. Function 5:zqad062 10.1093/function/zqad062 PMC10666992 38020067

[CR47] Isoyama N, Qureshi AR, Avesani CM, Lindholm B, Bàràny P, Heimbürger O, Cederholm T, Stenvinkel P, Carrero JJ (2014) Comparative associations of muscle mass and muscle strength with mortality in dialysis patients. Clin J Am Soc Nephrol 9:1720–1728 25074839 10.2215/CJN.10261013 PMC4186520

[CR48] Jones III RG, Dimet-Wiley A, Haghani A, da Silva FM, Brightwell CR, Lim S, Khadgi S, Wen Y, Dungan CM, Brooke RT et al (2022) A molecular signature defining exercise adaptation with ageing and in vivo partial reprogramming in skeletal muscle. J Physiol 601:763–782 10.1113/JP283836 PMC9987218 36533424

[CR49] Jozsi AC, Dupont-Versteegden EE, Taylor-Jones JM, Evans WJ, Trappe TA, Campbell WW, Peterson CA (2000) Aged human muscle demonstrates an altered gene expression profile consistent with an impaired response to exercise. Mech Age Dev 120:45–56 10.1016/s0047-6374(00)00178-0 11087903

[CR50] Kaneko Y, Shibuya M, Nakayama T, Hayashida N, Toda G, Endo Y, Oka H, Oda T (1985) Hypomethylation of c-myc and epidermal growth factor receptor genes in human hepatocellular carcinoma and fetal liver. Jpn J Cancer Res 76:1136–1140 3005205

[CR51] Kim D, Langmead B, Salzberg SL (2015) HISAT: a fast spliced aligner with low memory requirements. Nat Methods 12:357–360 25751142 10.1038/nmeth.3317 PMC4655817

[CR52] Kraniou Y, Cameron-Smith D, Misso M, Collier G, Hargreaves M (2000) Effects of exercise on GLUT-4 and glycogenin gene expression in human skeletal muscle. J Appl Physiol 88:794–796 10658052 10.1152/jappl.2000.88.2.794

[CR53] Kuang J, McGinley C, Lee MJ, Saner NJ, Garnham A, Bishop DJ (2022) Interpretation of exercise-induced changes in human skeletal muscle mRNA expression depends on the timing of the post-exercise biopsies. PeerJ 10:e12856 35186464 10.7717/peerj.12856 PMC8820226

[CR54] Kuleshov MV, Jones MR, Rouillard AD, Fernandez NF, Duan Q, Wang Z, Koplev S, Jenkins SL, Jagodnik KM, Lachmann A (2016) Enrichr: a comprehensive gene set enrichment analysis web server 2016 update. Nucleic Acid Res 44:W90–W97 27141961 10.1093/nar/gkw377 PMC4987924

[CR55] Lavin KM, Bell MB, McAdam JS, Peck BD, Walton RG, Windham ST, Tuggle SC, Long DE, Kern PA, Peterson CA et al (2021) Muscle transcriptional networks linked to resistance exercise training hypertrophic response heterogeneity. Physiol Genom 53:206–221 10.1152/physiolgenomics.00154.2020 PMC8424535 33870722

[CR56] Lavin KM, Graham ZA, McAdam JS, O’Bryan SM, Drummer D, Bell MB, Kelley CJ, Lixandrão ME, Peoples B, Tuggle SC et al (2023) Dynamic transcriptomic responses to acute exercise stimuli in young adults. Physiol Genom 55:194–212 10.1152/physiolgenomics.00144.2022 PMC10110731 36939205

[CR57] Li R, Xia J, Zhang X, Gathirua-Mwangi WG, Guo J, Li Y, McKenzie S, Song Y (2018) Associations of muscle mass and strength with all-cause mortality among US older adults. Med Sci Sport Exerc 50:458 10.1249/MSS.0000000000001448 PMC5820209 28991040

[CR58] Liao Y, Smyth GK, Shi W (2014) featureCounts: an efficient general purpose program for assigning sequence reads to genomic features. Bioinformatics 30:923–930 24227677 10.1093/bioinformatics/btt656

[CR59] Lindholm ME, Marabita F, Gomez-Cabrero D, Rundqvist H, Ekström TJ, Tegnér J, Sundberg CJ (2014) An integrative analysis reveals coordinated reprogramming of the epigenome and the transcriptome in human skeletal muscle after training. Epigenetics 9:1557–1569 25484259 10.4161/15592294.2014.982445 PMC4622000

[CR60] Louis E, Raue U, Yang Y, Jemiolo B, Trappe S (2007) Time course of proteolytic, cytokine, and myostatin gene expression after acute exercise in human skeletal muscle. J Appl Physiol 103:1744–1751 17823296 10.1152/japplphysiol.00679.2007

[CR61] Love MI, Huber W, Anders S (2014) Moderated estimation of fold change and dispersion for RNA-seq data with DESeq2. Genom Biol 15:1–21 10.1186/s13059-014-0550-8 PMC4302049 25516281

[CR62] Lovrić A, Rassolie A, Alam S, Mandić M, Saini A, Altun M, Fernandez-Gonzalo R, Gustafsson T, Rullman E (2022) Single-cell sequencing deconvolutes cellular responses to exercise in human skeletal muscle. Comm Biol 5:1121 10.1038/s42003-022-04088-z PMC9588010 36273106

[CR63] Lüscher B (2001) Function and regulation of the transcription factors of the Myc/Max/Mad network. Gene 277:1–14 11602341 10.1016/s0378-1119(01)00697-7

[CR64] Mahoney D, Parise G, Melov S, Safdar A, Tarnopolsky M (2005) Analysis of global mRNA expression in human skeletal muscle during recovery from endurance exercise. FASEB J 19:1498–1500 15985525 10.1096/fj.04-3149fje

[CR65] Mammucari C, Milan G, Romanello V, Masiero E, Rudolf R, Del Piccolo P, Burden SJ, Di Lisi R, Sandri C, Zhao J et al (2007) FoxO3 controls autophagy in skeletal muscle in vivo. Cell Metab 6:458–471 18054315 10.1016/j.cmet.2007.11.001

[CR66] McCarthy JJ, Andrews JL, McDearmon EL, Campbell KS, Barber BK, Miller BH, Walker JR, Hogenesch JB, Takahashi JS, Esser KA (2007) Identification of the circadian transcriptome in adult mouse skeletal muscle. Physiol Genom 31:86–95 10.1152/physiolgenomics.00066.2007 PMC6080860 17550994

[CR67] McKellar DW, Walter LD, Song LT, Mantri M, Wang MF, De Vlaminck I, Cosgrove BD (2021) Large-scale integration of single-cell transcriptomic data captures transitional progenitor states in mouse skeletal muscle regeneration. Comm Biol 4:1–12 10.1038/s42003-021-02810-x PMC8589952 34773081

[CR68] Melkani GC, Bhide S, Han A, Vyas J, Livelo C, Bodmer R, Bernstein SI (2017) TRiC/CCT chaperonins are essential for maintaining myofibril organization, cardiac physiological rhythm, and lifespan. FEBS Lett 591:3447–3458 28963798 10.1002/1873-3468.12860 PMC5683924

[CR69] Metter EJ, Talbot LA, Schrager M, Conwit R (2002) Skeletal muscle strength as a predictor of all-cause mortality in healthy men. J Gerontol Ser A 57:B359–B365 10.1093/gerona/57.10.b359 12242311

[CR70] Moberg M, Apró W, Ekblom B, Van Hall G, Holmberg H-C, Blomstrand E (2016) Activation of mTORC1 by leucine is potentiated by branched-chain amino acids and even more so by essential amino acids following resistance exercise. Am J Physiol Cell Physiol 310:C874–C884 27053525 10.1152/ajpcell.00374.2015

[CR71] Mori T, Ato S, Knudsen JR, Henriquez-Olguin C, Li Z, Wakabayashi K, Suginohara T, Higashida K, Tamura Y, Nakazato K et al (2020) C-Myc overexpression increases ribosome biogenesis and protein synthesis independent of mTORC1 activation in mouse skeletal muscle. Am J Physiol Endocrinol Metab 321:E551–E559 10.1152/ajpendo.00164.2021 34423683

[CR72] Murach K, Raue U, Wilkerson B, Minchev K, Jemiolo B, Bagley J, Luden N, Trappe S (2014) Single muscle fiber gene expression with run taper. PloS One 9:e108547 25268477 10.1371/journal.pone.0108547 PMC4182496

[CR73] Murach KA, Dimet-Wiley AL, Wen Y, Brightwell CR, Latham CM, Dungan CM, Fry CS, Watowich SJ (2021a) Late-life exercise mitigates skeletal muscle epigenetic aging. Aging Cell. 10.1111/acel.13527 10.1111/acel.13527 PMC8761012 34932867

[CR74] Murach KA, Dungan CM, von Walden F, Wen Y (2021b) Epigenetic evidence for distinct contributions of resident and acquired myonuclei during long-term exercise adaptation using timed in vivo myonuclear labeling. Am J Physiol Cell Physiol 32:C86–C93 10.1152/ajpcell.00358.2021 PMC8765804 34817266

[CR75] Murach KA, Liu Z, Jude B, Figueiredo VC, Wen Y, Khadgi S, Lim S, da Silva FM, Greene NP, Lanner JT et al (2022) Multi-transcriptome analysis following an acute skeletal muscle growth stimulus yields tools for discerning global and MYC regulatory networks. J Biol Chem 298:102515 36150502 10.1016/j.jbc.2022.102515 PMC9583450

[CR76] Murach KA, Mobley CB, Zdunek CJ, Frick KK, Jones SR, McCarthy JJ, Peterson CA, Dungan CM (2020) Muscle memory: myonuclear accretion, maintenance, morphology, and miRNA levels with training and detraining in adult mice. J Cahcexia Sarcopenia Muscle 11:1705–1722 10.1002/jcsm.12617 PMC7749570 32881361

[CR77] Murach KA, Peck BD, Policastro RA, Vechetti IJ, Van Pelt DW, Dungan CM, Denes LT, Fu X, Brightwell CR, Zentner GE et al (2021c) Early satellite cell communication creates a permissive environment for long-term muscle growth. iScience 24:102372 33948557 10.1016/j.isci.2021.102372 PMC8080523

[CR78] Neubauer O, Sabapathy S, Ashton KJ, Desbrow B, Peake JM, Lazarus R, Wessner B, Cameron-Smith D, Wagner K-H, Haseler LJ (2014) Time course-dependent changes in the transcriptome of human skeletal muscle during recovery from endurance exercise: from inflammation to adaptive remodeling. J Appl Physiol 116:274–287 24311745 10.1152/japplphysiol.00909.2013

[CR79] Newman AB, Kupelian V, Visser M, Simonsick EM, Goodpaster BH, Kritchevsky SB, Tylavsky FA, Rubin SM, Harris TB (2006) Strength, but not muscle mass, is associated with mortality in the health, aging and body composition study cohort. J Gerontol Ser A 61:72–77 10.1093/gerona/61.1.72 16456196

[CR80] Newman AM, Steen CB, Liu CL, Gentles AJ, Chaudhuri AA, Scherer F, Khodadoust MS, Esfahani MS, Luca BA, Steiner D (2019) Determining cell type abundance and expression from bulk tissues with digital cytometry. Nat Biotech 37:773–782 10.1038/s41587-019-0114-2 PMC6610714 31061481

[CR81] Pascoe AL, Johnston AJ, Murphy RM (2020) Controversies in TWEAK-Fn14 signaling in skeletal muscle atrophy and regeneration. Cell Mol Life Sci 77:3369–3381 10.1007/s00018-020-03495-x PMC11104974 32200423

[CR82] Perrin L, Loizides-Mangold U, Chanon S, Gobet C, Hulo N, Isenegger L, Weger BD, Migliavacca E, Charpagne A, Betts JA (2018) Transcriptomic analyses reveal rhythmic and CLOCK-driven pathways in human skeletal muscle. eLife 7:e34114 29658882 10.7554/eLife.34114 PMC5902165

[CR83] Perry CG, Lally J, Holloway GP, Heigenhauser GJ, Bonen A, Spriet LL (2010) Repeated transient mRNA bursts precede increases in transcriptional and mitochondrial proteins during training in human skeletal muscle. J Physiol 588:4795–4810 20921196 10.1113/jphysiol.2010.199448 PMC3010147

[CR84] Phillips BE, Williams JP, Gustafsson T, Bouchard C, Rankinen T, Knudsen S, Smith K, Timmons JA, Atherton PJ et al (2013) Molecular networks of human muscle adaptation to exercise and age. PLoS Genet 9:e1003389 23555298 10.1371/journal.pgen.1003389 PMC3605101

[CR85] Pilegaard H, Ordway GA, Saltin B, Neufer PD (2000) Transcriptional regulation of gene expression in human skeletal muscle during recovery from exercise. Am J Physiol Endocrinol Metab 279:E806–E814 11001762 10.1152/ajpendo.2000.279.4.E806

[CR86] Pilegaard H, Saltin B, Neufer PD (2003) Exercise induces transient transcriptional activation of the PGC-1α gene in human skeletal muscle. Wiley Online Libr 546:851–858 10.1113/jphysiol.2002.034850 PMC2342594 12563009

[CR87] Pillon NJ, Gabriel BM, Dollet L, Smith JA, Puig LS, Botella J, Bishop DJ, Krook A, Zierath JR (2020) Transcriptomic profiling of skeletal muscle adaptations to exercise and inactivity. Nat Commun 11:1–15 31980607 10.1038/s41467-019-13869-w PMC6981202

[CR88] Poliacikova G, Maurel-Zaffran C, Graba Y, Saurin AJ (2021) Hox proteins in the regulation of muscle development. Front Cell Dev Biol 9:731996 34733846 10.3389/fcell.2021.731996 PMC8558437

[CR89] Qin Q, Fan J, Zheng R, Wan C, Mei S, Wu Q, Sun H, Brown M, Zhang J, Meyer CA (2020) Lisa: inferring transcriptional regulators through integrative modeling of public chromatin accessibility and ChIP-seq data. Genome Biol 21:32 32033573 10.1186/s13059-020-1934-6 PMC7007693

[CR90] Rao P, Antony A, Rajalakshmi S, Sarma D (1989) Studies on hypomethylation of liver DNA during early stages of chemical carcinogenesis in rat liver. Carcinogenesis 10:933–937 2650909 10.1093/carcin/10.5.933

[CR91] Raue U, Jemiolo B, Yang Y, Trappe S (2015) TWEAK-Fn14 pathway activation after exercise in human skeletal muscle: insights from two exercise modes and a time course investigation. J Appl Physiol 118:569–578 25539934 10.1152/japplphysiol.00759.2014 PMC4346742

[CR92] Raue U, Trappe TA, Estrem ST, Qian HR, Helvering LM, Smith RC, Trappe S (2012) Transcriptome signature of resistance exercise adaptations: mixed muscle and fiber type specific profiles in young and old adults. J Appl Physiol 112:1625–1636 22302958 10.1152/japplphysiol.00435.2011 PMC3365403

[CR93] Reitzner SM, Emanuelsson EB, Arif M, Kaczkowski B, Kwon AT, Mardinoglu A, Arner E, Chapman MA, Sundberg CJ (2024) Molecular profiling of high-level athlete skeletal muscle after acute endurance or resistance exercise—a systems biology approach. Mol Metab 79:101857 38141850 10.1016/j.molmet.2023.101857 PMC10805945

[CR94] Roberts JZ, Crawford N, Longley DB (2022) The role of ubiquitination in apoptosis and necroptosis. Cell Death Differ 29:272–284 34912054 10.1038/s41418-021-00922-9 PMC8817035

[CR95] Roberts MD, McCarthy JJ, Hornberger TA, Phillips SM, Mackey AL, Nader GA, Boppart MD, Kavazis AN, Reidy PT, Ogasawara R et al (2023) Mechanisms of mechanical overload-induced skeletal muscle hypertrophy: current understanding and future directions. Physiol Rev 103:2679–2757 37382939 10.1152/physrev.00039.2022 PMC10625844

[CR96] Robinson MM, Dasari S, Konopka AR, Johnson ML, Manjunatha S, Esponda RR, Carter RE, Lanza IR, Nair KS (2017) Enhanced protein translation underlies improved metabolic and physical adaptations to different exercise training modes in young and old humans. Cell Metab 25:581–592 28273480 10.1016/j.cmet.2017.02.009 PMC5423095

[CR97] Sandri M, Lin J, Handschin C, Yang W, Arany ZP, Lecker SH, Goldberg AL, Spiegelman BM (2006) PGC-1α protects skeletal muscle from atrophy by suppressing FoxO3 action and atrophy-specific gene transcription. Proc Nat Acad Sci USA 103:16260–16265 17053067 10.1073/pnas.0607795103 PMC1637570

[CR98] Sanford JA, Nogiec CD, Lindholm ME, Adkins JN, Amar D, Dasari S, Drugan JK, Fernández FM, Radom-Aizik S, Schenk S (2020) Molecular transducers of physical activity consortium (MoTrPAC): mapping the dynamic responses to exercise. Cell 181:1464–1474 32589957 10.1016/j.cell.2020.06.004 PMC8800485

[CR99] Seaborne R, Strauss J, Cocks M, Shepherd S, O’Brien T, Van Someren K, Bell P, Murgatroyd C, Morton J, Stewart C et al (2018a) Methylome of human skeletal muscle after acute & chronic resistance exercise training, detraining & retraining. Sci Data 5:180213 30375987 10.1038/sdata.2018.213 PMC6207066

[CR100] Seaborne RA, Strauss J, Cocks M, Shepherd S, O’Brien TD, van Someren KA, Bell PG, Murgatroyd C, Morton JP, Stewart CE, Sharples AP (2018b) Human skeletal muscle possesses an epigenetic memory of hypertrophy. Sci Rep 8:1898 29382913 10.1038/s41598-018-20287-3 PMC5789890

[CR101] Segalés J, Perdiguero E, Serrano AL, Sousa-Victor P, Ortet L, Jardí M, Budanov AV, Garcia-Prat L, Sandri M, Thomson DM (2020) Sestrin prevents atrophy of disused and aging muscles by integrating anabolic and catabolic signals. Nat Commun 11:189 31929511 10.1038/s41467-019-13832-9 PMC6955241

[CR102] Sharples AP, Seaborne RA (2019) Exercise and DNA methylation in skeletal muscle. In: Sports, exercise, and nutritional genomics. Elsevier, pp. 211–229

[CR103] Sidorenko S, Klimanova E, Milovanova K, Lopina OD, Kapilevich LV, Chibalin AV, Orlov SN (2018) Transcriptomic changes in C2C12 myotubes triggered by electrical stimulation: role of Ca^2+^_i_-mediated and Ca^2+^_i_-independent signaling and elevated [Na+]_i_/[K+]_i_ ratio. Cell Calcium 76:72–86 30300758 10.1016/j.ceca.2018.09.007

[CR104] Small L, Ingerslev LR, Manitta E, Laker RC, Hansen AN, Deeney B, Carrié A, Couvert P, Barrès R (2021) Ablation of DNA-methyltransferase 3A in skeletal muscle does not affect energy metabolism or exercise capacity. PLoS Genet 17:e1009325 33513138 10.1371/journal.pgen.1009325 PMC7875352

[CR105] Smith JAB, Murach KA, Dyar KA, Zierath JR (2023) Exercise metabolism and adaptation in skeletal muscle. Nat Rev Mol Cell Biol 24:607–632 37225892 10.1038/s41580-023-00606-x PMC10527431

[CR106] Sparks LM (2017) Exercise training response heterogeneity: physiological and molecular insights. Diabetologia 60:2329–2336 29032385 10.1007/s00125-017-4461-6

[CR107] Stec MJ, Kelly NA, Many GM, Windham ST, Tuggle SC, Bamman MM (2016) Ribosome biogenesis may augment resistance training-induced myofiber hypertrophy and is required for myotube growth in vitro. Am J Physiol Endo Metab 310:E652 10.1152/ajpendo.00486.2015 PMC4835943 26860985

[CR108] Stokes T, Cen HH, Kapranov P, Gallagher IJ, Pitsillides AA, Volmar CH, Kraus WE, Johnson JD, Phillips SM, Wahlestedt C et al (2023) Transcriptomics for clinical and experimental biology research: hang on a Seq. Adv Genet 4:2200024 37288167 10.1002/ggn2.202200024 PMC10242409

[CR109] Tang Q, Chen Y, Meyer C, Geistlinger T, Lupien M, Wang Q, Liu T, Zhang Y, Brown M, Liu XS (2011) A comprehensive view of nuclear receptor cancer cistromes. Cancer Res 71:6940–6947 21940749 10.1158/0008-5472.CAN-11-2091 PMC3610570

[CR110] Tomaz da Silva M, Joshi AS, Koike TE, Roy A, Mathukumalli K, Sopariwala DH, Narkar VA, Kumar A (2022) Targeted ablation of Fn14 receptor improves exercise capacity and inhibits neurogenic muscle atrophy. FASEB J 36:e22666 36412933 10.1096/fj.202201583R PMC10587854

[CR111] Townsend JR, Stout JR, Jajtner AR, Church DD, Beyer KS, Oliveira LP, La Monica MB, Riffe JJ, Muddle TW, Baker KM (2016) Resistance exercise increases intramuscular NF-κb signaling in untrained males. Eur J Appl Physiol 116:2103–2111 27582262 10.1007/s00421-016-3463-2

[CR112] Tsujiuchi T, Tsutsumi M, Sasaki Y, Takahama M, Konishi Y (1999) Hypomethylation of CpG sites and c-myc gene overexpression in hepatocellular carcinomas, but not hyperplastic nodules, induced by a choline-deficient L-amino acid-defined diet in rats. Jpn J Cancer Res 90:909–913 10551317 10.1111/j.1349-7006.1999.tb00834.x PMC5926157

[CR113] Tsumagari K, Baribault C, Terragni J, Chandra S, Renshaw C, Sun Z, Song L, Crawford GE, Pradhan S, Lacey M (2013) DNA methylation and differentiation: HOX genes in muscle cells. Epigenetics Chromatin 6:1–17 23916067 10.1186/1756-8935-6-25 PMC3750649

[CR114] Turner DC, Gorski PP, Maasar M, Seaborne RA, Baumert P, Brown AD, Kitchen M, Erskine R, Dos-Remedios I, Voisin S et al (2020) DNA methylation across the genome in aged human skeletal muscle tissue and muscle-derived cells: the role of HOX genes and physical activity. Sci Rep 10:1–19 32958812 10.1038/s41598-020-72730-z PMC7506549

[CR115] Turner DC, Seaborne RA, Sharples AP (2019) Comparative transcriptome and methylome analysis in human skeletal muscle anabolism, hypertrophy and epigenetic memory. Sci Rep 9:1–12 30862794 10.1038/s41598-019-40787-0 PMC6414679

[CR116] Viggars MR, Sutherland H, Lanmüller H, Schmoll M, Bijak M, Jarvis JC (2022a) Adaptation of the transcriptional response to resistance exercise over 4 weeks of daily training. FASEB J 37:e22686 10.1096/fj.202201418R PMC13281843 36468768

[CR117] Viggars MR, Wen Y, Peterson CA, Jarvis JC (2022b) Automated cross-sectional analysis of trained, severely atrophied and recovering rat skeletal muscles using MyoVision 2.0. J Appl Physiol 132:593–610 35050795 10.1152/japplphysiol.00491.2021

[CR118] Villivalam SD, Ebert SM, Lim HW, Kim J, You D, Jung BC, Palacios HH, Tcheau T, Adams CM, Kang S (2021) A necessary role of DNMT3A in endurance exercise by suppressing ALDH1L1-mediated oxidative stress. EMBO J 40:e106491 10.15252/embj.2020106491 PMC8090849 33847380

[CR119] Vissing K, Andersen JL, Schjerling P (2005) Are exercise-induced genes induced by exercise? FASEB J 19:94–96 15516373 10.1096/fj.04-2084fje

[CR120] Voisin S, Jacques M, Landen S, Harvey NR, Haupt LM, Griffiths LR, Gancheva S, Ouni M, Jähnert M, Ashton KJ (2021) Meta-analysis of genome-wide DNA methylation and integrative omics of age in human skeletal muscle. J Cachexia Sarcopenia Muscle 12:1064–1078 34196129 10.1002/jcsm.12741 PMC8350206

[CR121] von Walden F (2019) Ribosome biogenesis in skeletal muscle: coordination of transcription and translation. J Appl Physiol 127:591–598 31219775 10.1152/japplphysiol.00963.2018

[CR122] von Walden F, Casagrande V, Östlund Farrants A-K, Nader GA (2012) Mechanical loading induces the expression of a Pol I regulon at the onset of skeletal muscle hypertrophy. Am J Physiol Cell Physiol 302:C1523–C1530 22403788 10.1152/ajpcell.00460.2011

[CR123] von Walden F, Fernandez-Gonzalo R, Norrbom J, Emanuelsson EB, Figueiredo VC, Gidlund E-K, Norrbrand L, Liu C, Sandström P, Hansson B (2021) Acute endurance exercise stimulates circulating levels of mitochondrial-derived peptides in humans. J Appl Physiol 131:1035–1042 34351816 10.1152/japplphysiol.00706.2019 PMC12854548

[CR124] von Walden F, Rea M, Mobley CB, Fondufe-Mittendorf Y, McCarthy JJ, Peterson CA, Murach KA (2020) The myonuclear DNA methylome in response to an acute hypertrophic stimulus. Epigenetics 15:1151–1162 32281477 10.1080/15592294.2020.1755581 PMC7595631

[CR125] Walton RG, Kosmac K, Mula J, Fry CS, Peck BD, Groshong JS, Finlin BS, Zhu B, Kern PA, Peterson CA (2019) Human skeletal muscle macrophages increase following cycle training and are associated with adaptations that may facilitate growth. Sci Rep 9:1–14 30700754 10.1038/s41598-018-37187-1 PMC6353900

[CR126] Wang C, Ros RR, Martinez-Redondo P, Ma Z, Shi L, Xue Y, Guillen-Guillen I, Huang L, Hishida T, Liao H-K (2021) In vivo partial reprogramming of myofibers promotes muscle regeneration by remodeling the stem cell niche. Nat Commun 12:1–15 34035273 10.1038/s41467-021-23353-z PMC8149870

[CR127] Wang H, Lu J, Stevens T, Roberts A, Mandel J, Avula R, Ma B, Wu Y, Wang J, Van’t Land C (2023) Premature aging and reduced cancer incidence associated with near-complete body-wide Myc inactivation. Cell Rep 42:112830 37481724 10.1016/j.celrep.2023.112830 PMC10591215

[CR128] Wang H-Q, Tuominen LK, Tsai C-J (2011) SLIM: a sliding linear model for estimating the proportion of true null hypotheses in datasets with dependence structures. Bioinformatics 27:225–231 21098430 10.1093/bioinformatics/btq650

[CR129] Wang S, Sun H, Ma J, Zang C, Wang C, Wang J, Tang Q, Meyer CA, Zhang Y, Liu XS (2013) Target analysis by integration of transcriptome and ChIP-seq data with BETA. Nat Protoc 8:2502–2515 24263090 10.1038/nprot.2013.150 PMC4135175

[CR130] Wang X, Blagden C, Fan J, Nowak SJ, Taniuchi I, Littman DR, Burden SJ (2005) Runx1 prevents wasting, myofibrillar disorganization, and autophagy of skeletal muscle. Genes Dev 19:1715–1722 16024660 10.1101/gad.1318305 PMC1176009

[CR131] Wen Y, Alimov AP, McCarthy JJ (2016) Ribosome biogenesis is necessary for skeletal muscle hypertrophy. Exerc Sport Sci Rev 44:110 27135313 10.1249/JES.0000000000000082 PMC4911282

[CR132] Wen Y, Murach KA, Vechetti JrIJ, Fry CS, Vickery CD, Peterson CA, McCarthy JJ, Campbell KS (2017) MyoVision: software for automated high-content analysis of skeletal muscle immunohistochemistry. J Appl Physiol 124:40–51 28982947 10.1152/japplphysiol.00762.2017 PMC6048460

[CR133] West DW, Baehr LM, Marcotte GR, Chason CM, Tolento L, Gomes AV, Bodine SC, Baar K (2016) Acute resistance exercise activates rapamycin-sensitive and-insensitive mechanisms that control translational activity and capacity in skeletal muscle. J Physiol 594:453–468 26548696 10.1113/JP271365 PMC4713726

[CR134] Xie Z, Bailey A, Kuleshov MV, Clarke DJ, Evangelista JE, Jenkins SL, Lachmann A, Wojciechowicz ML, Kropiwnicki E, Jagodnik KM (2021) Gene set knowledge discovery with Enrichr. Curr Protoc 1:e90 33780170 10.1002/cpz1.90 PMC8152575

[CR135] Yang Y, Creer A, Jemiolo B, Trappe S (2005) Time course of myogenic and metabolic gene expression in response to acute exercise in human skeletal muscle. J Appl Physiol 98:1745–1752 15618316 10.1152/japplphysiol.01185.2004

[CR136] Zambon AC, McDearmon EL, Salomonis N, Vranizan KM, Johansen KL, Adey D, Takahashi JS, Schambelan M, Conklin BR (2003) Time- and exercise-dependent gene regulation in human skeletal muscle. Genome Biol 4:1–12 10.1186/gb-2003-4-10-r61 PMC328450 14519196

[CR137] Zhao J, Brault JJ, Schild A, Cao P, Sandri M, Schiaffino S, Lecker SH, Goldberg AL (2007) FoxO3 coordinately activates protein degradation by the autophagic/lysosomal and proteasomal pathways in atrophying muscle cells. Cell Metab 6:472–483 18054316 10.1016/j.cmet.2007.11.004

