# Supplementary material for: The 24-hour molecular landscape after exercise in humans reveals MYC is sufficient for muscle growth
Source: EMBO Rep. 2024 Oct 31;25(12):5810–37. doi: 10.1038/s44319-024-00299-z (PMC11624283; doi:10.1038/s44319-024-00299-z)
Supplement: Supplementary file 1 — Appendix [file 44319_2024_299_MOESM1_ESM.pdf]

Appendix Figures

Table of Content

- Appendix Figure S1. 2
    - Figure Text Appendix Figure S1. 3
- Appendix Figure S2. 4
    - Figure Text Appendix Figure S2. 5
- Appendix Figure S3. 6
    - Figure Text Appendix Figure S3. 7

Appendix Figure S1

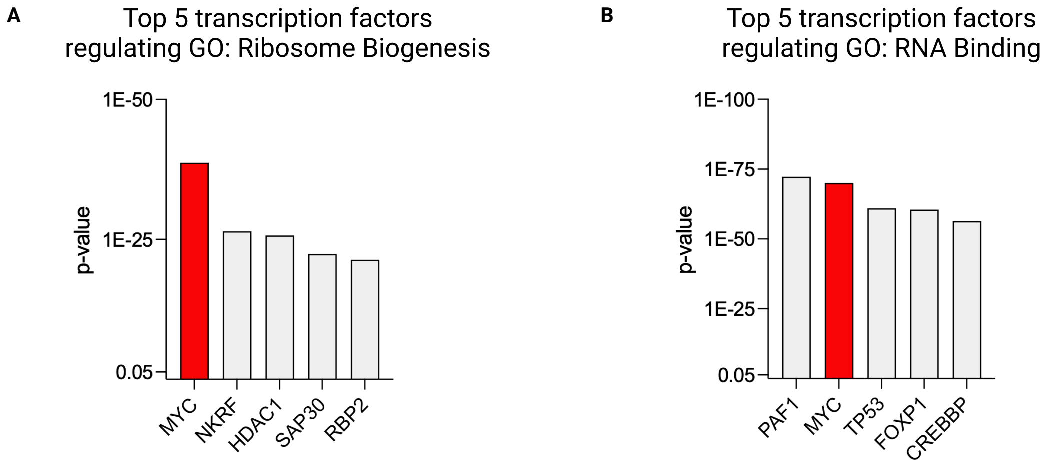

Appendix Figure S1. Transcription factors associated with the most highly enriched Gene Ontology pathways. (A) Top 5 transcription factors predicted to be associated with genes regulating Ribosomal Biogenesis in our data set. (B) Top 5 transcription factors predicted to be associated with genes regulating RNA-binding in our data set.

Appendix Figure S2

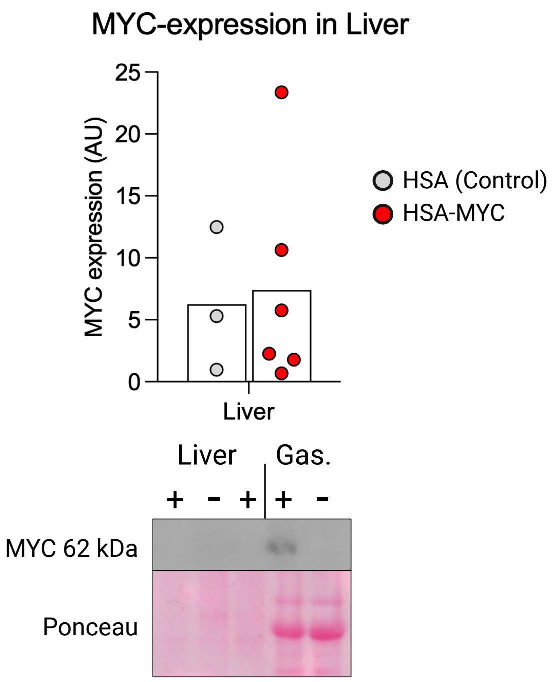

Appendix Figure S2. Levels of MYC protein in liver following 48 hours of doxycycline administration. Expression of MYC in liver samples of HSA-control, and HSA-MYC mice following 48 hours of administration. Gas. = Gastrocnemius. Dots are biological replicates.

Appendix Figure S3

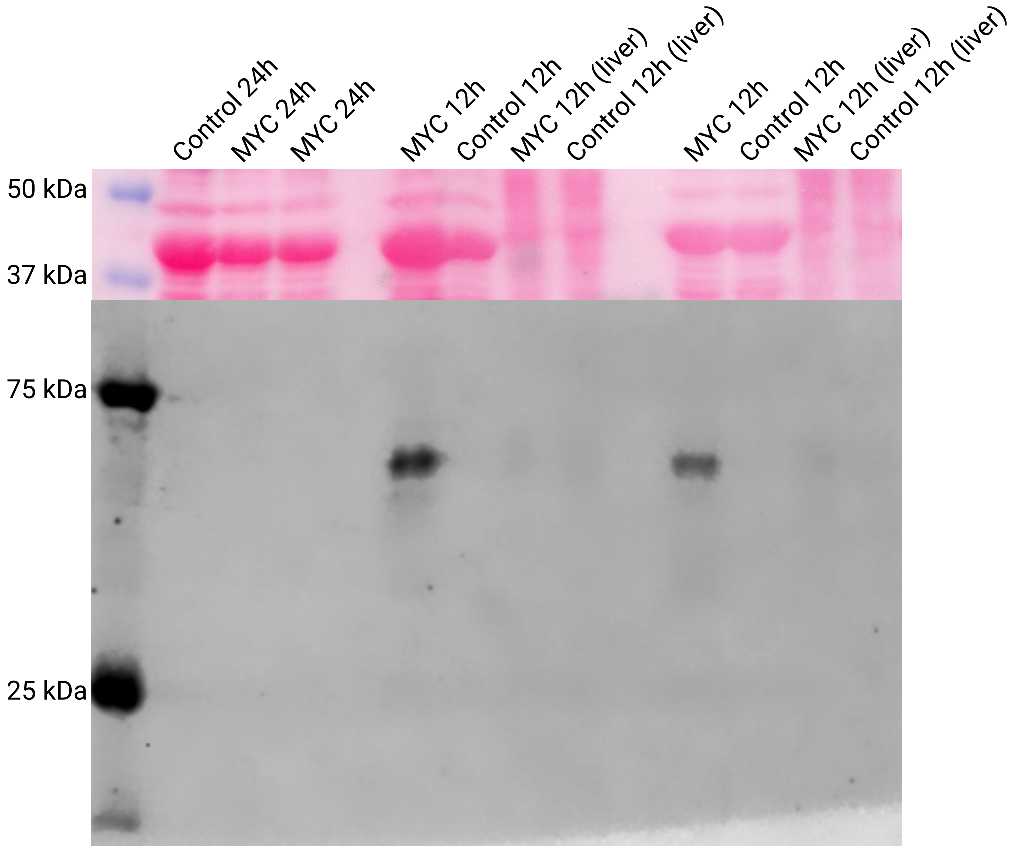

Appendix Figure S3. Levels of MYC protein return to baseline after 24 hours cessation from supplemented water administration. HSA-MYC and HSA littermate control mice were treated with doxycycline in drinking water for 12 hours, followed by a 24-hour treatment with un-supplemented drinking water. Quadriceps muscle was harvested in the morning and western blots for MYC were carried out as described in our previous publication (Jones III *et al*, 2022). Lanes 1-3 show one doxycycline-treated HSA and two HSA-MYC mice following 12 hours of doxycycline and 24 hours of un-supplemented water. MYC is not expressed in HSA mice and levels return to baseline levels by 24 hours in HSA-MYC mice (no MYC protein detectable). Lanes 4 & 8 show quadriceps muscle expression in HSA-MYC mice after 12 hours of treatment with doxycycline followed by a 12-hour chase (positive control, see Jones III et al 2022). Lanes 5 & 9 show the HSA control mice after the same 12-hour chase (negative control). Lanes 6 & 10 and 7 & 11 show MYC expression in liver of corresponding animals (another negative control).
