# Supplementary material for: The 24-hour molecular landscape after exercise in humans reveals MYC is sufficient for muscle growth
Source: EMBO Rep. 2024 Oct 31;25(12):5810–37. doi: 10.1038/s44319-024-00299-z (PMC11624283; doi:10.1038/s44319-024-00299-z)
Supplement: Supplementary file 11 — Source data Fig. 6 [file 44319_2024_299_MOESM11_ESM.zip › Figure 6/6G/Rep Images for Revision.pptx]

## Slide 1
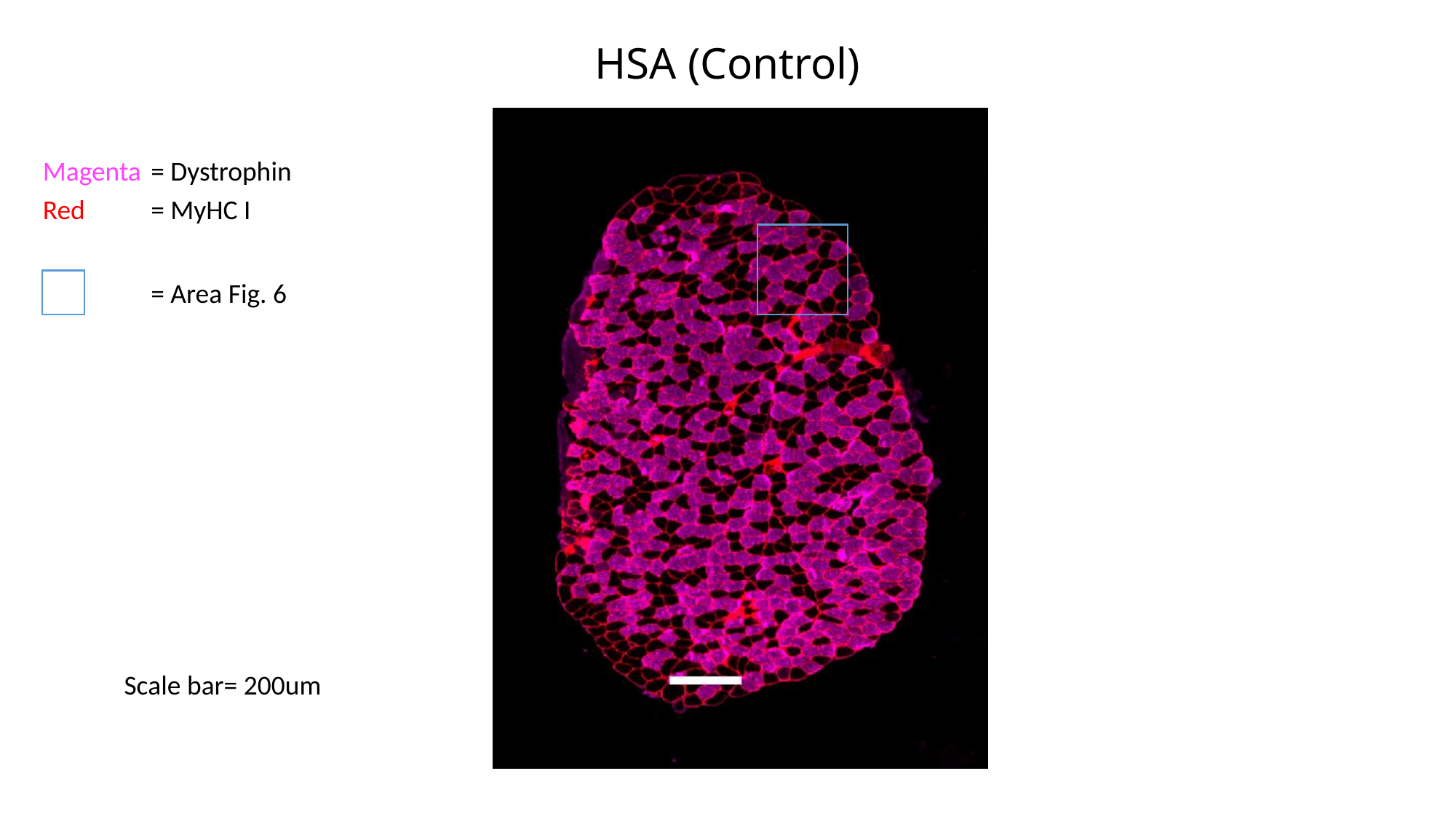

HSA (Control)
Magenta
= Dystrophin
Red
= MyHC I
= Area Fig. 6
Scale bar= 200um

## Slide 2
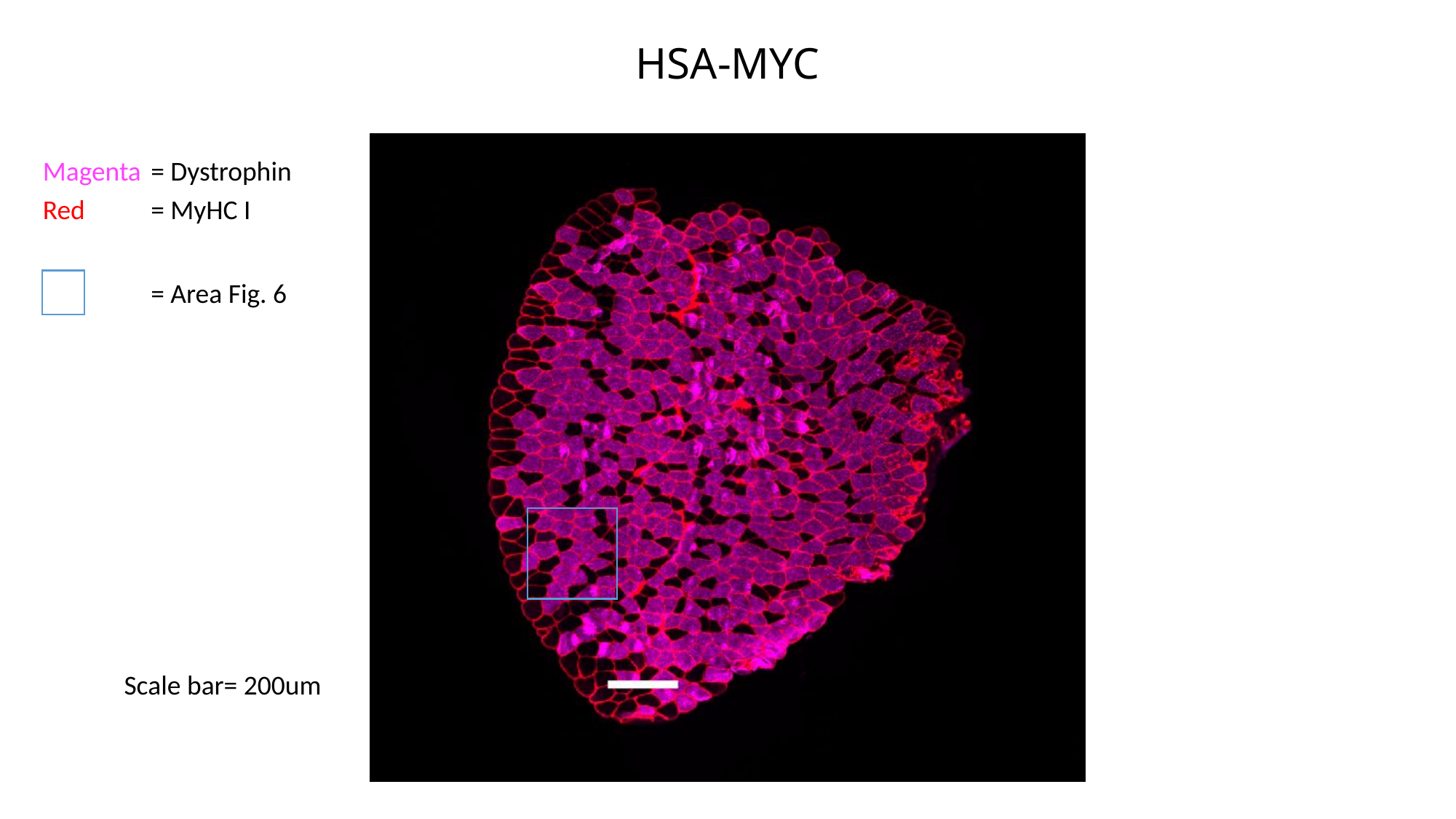

HSA-MYC
Magenta
= Dystrophin
Red
= MyHC I
= Area Fig. 6
Scale bar= 200um
