# Supplementary material for: The 24-hour molecular landscape after exercise in humans reveals MYC is sufficient for muscle growth
Source: EMBO Rep. 2024 Oct 31;25(12):5810–37. doi: 10.1038/s44319-024-00299-z (PMC11624283; doi:10.1038/s44319-024-00299-z)
Supplement: Supplementary file 11 — Source data Fig. 6 [file 44319_2024_299_MOESM11_ESM.zip › Figure 6/6B/Western images Muscle data.pptx]

## Slide 1
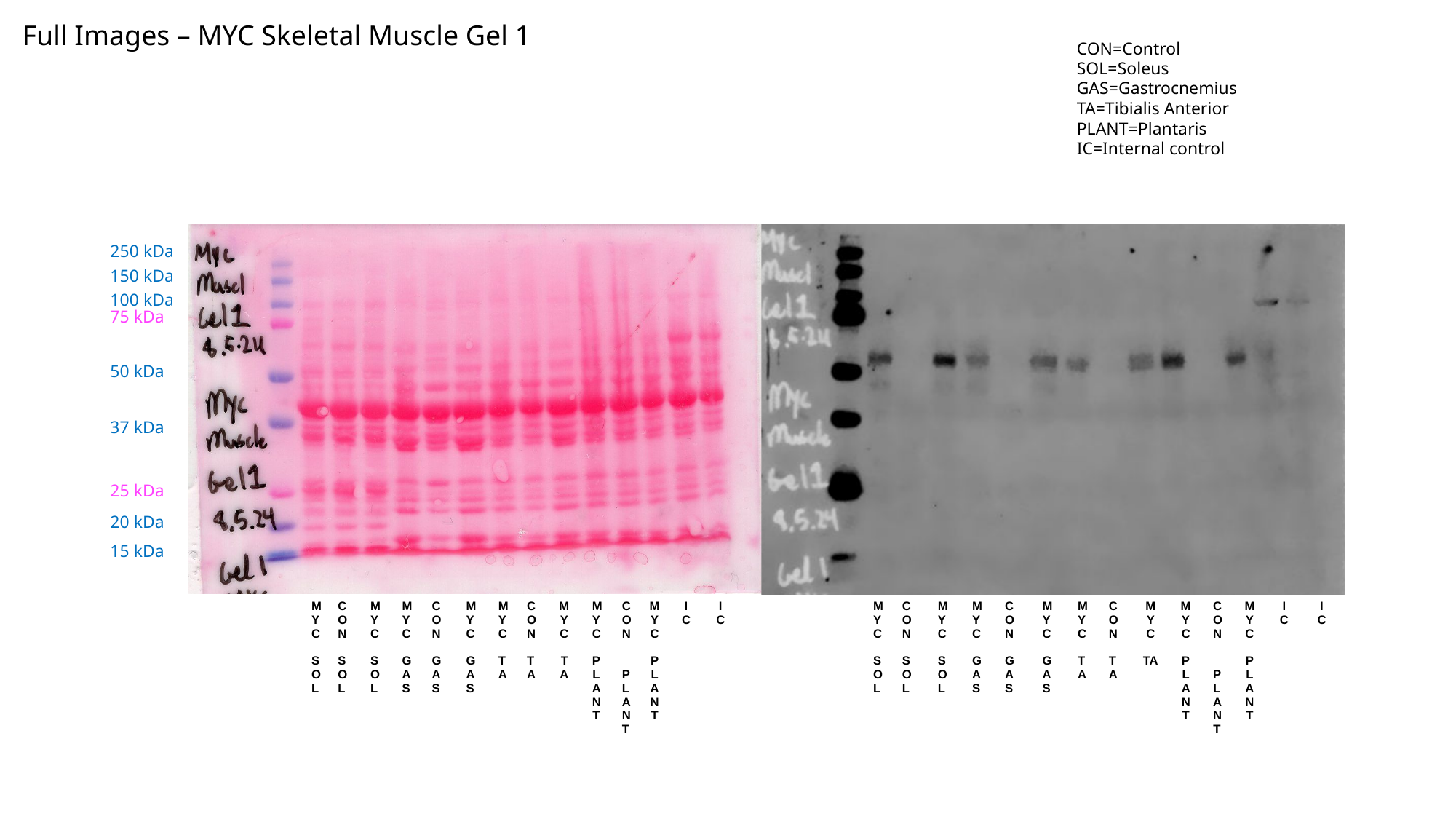

Full Images – MYC Skeletal Muscle Gel 1
CON=Control
SOL=Soleus
GAS=Gastrocnemius
TA=Tibialis Anterior
PLANT=Plantaris
IC=Internal control
250 kDa
150 kDa
100 kDa
75 kDa
50 kDa
37 kDa
25 kDa
20 kDa
15 kDa
| MYC SOL | CON SOL | MYC SOL | MYC GAS | CON GAS | MYC GAS | MYC TA | C O N T A | MYC TA | MYC PLANT | CON PLANT | MYC PLANT | IC | I C |
| --- | --- | --- | --- | --- | --- | --- | --- | --- | --- | --- | --- | --- | --- |
| MYC SOL | CON SOL | MYC SOL | MYC GAS | CON GAS | MYC GAS | MYC TA | C O N T A | MYC TA | MYC PLANT | CON PLANT | MYC PLANT | IC | I C |
| --- | --- | --- | --- | --- | --- | --- | --- | --- | --- | --- | --- | --- | --- |

## Slide 2
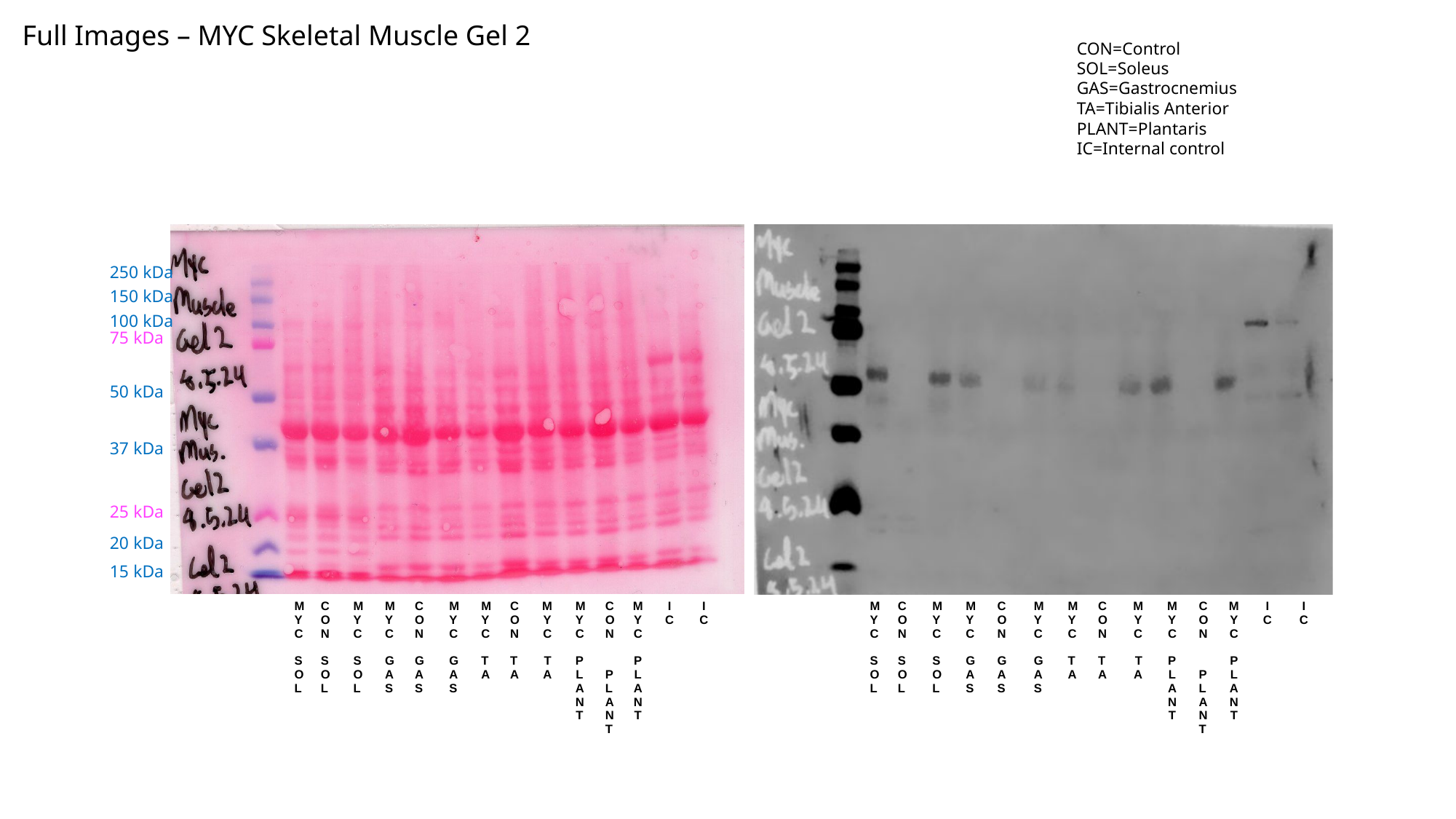

Full Images – MYC Skeletal Muscle Gel 2
CON=Control
SOL=Soleus
GAS=Gastrocnemius
TA=Tibialis Anterior
PLANT=Plantaris
IC=Internal control
250 kDa
150 kDa
100 kDa
75 kDa
50 kDa
37 kDa
25 kDa
20 kDa
15 kDa
| MYC SOL | CON SOL | MYC SOL | MYC GAS | CON GAS | MYC GAS | MYC TA | C O N T A | MYC TA | MYC PLANT | CON PLANT | MYC PLANT | IC | I C |
| --- | --- | --- | --- | --- | --- | --- | --- | --- | --- | --- | --- | --- | --- |
| MYC SOL | CON SOL | MYC SOL | MYC GAS | CON GAS | MYC GAS | MYC TA | C O N T A | MYC TA | MYC PLANT | CON PLANT | MYC PLANT | IC | I C |
| --- | --- | --- | --- | --- | --- | --- | --- | --- | --- | --- | --- | --- | --- |

## Slide 3
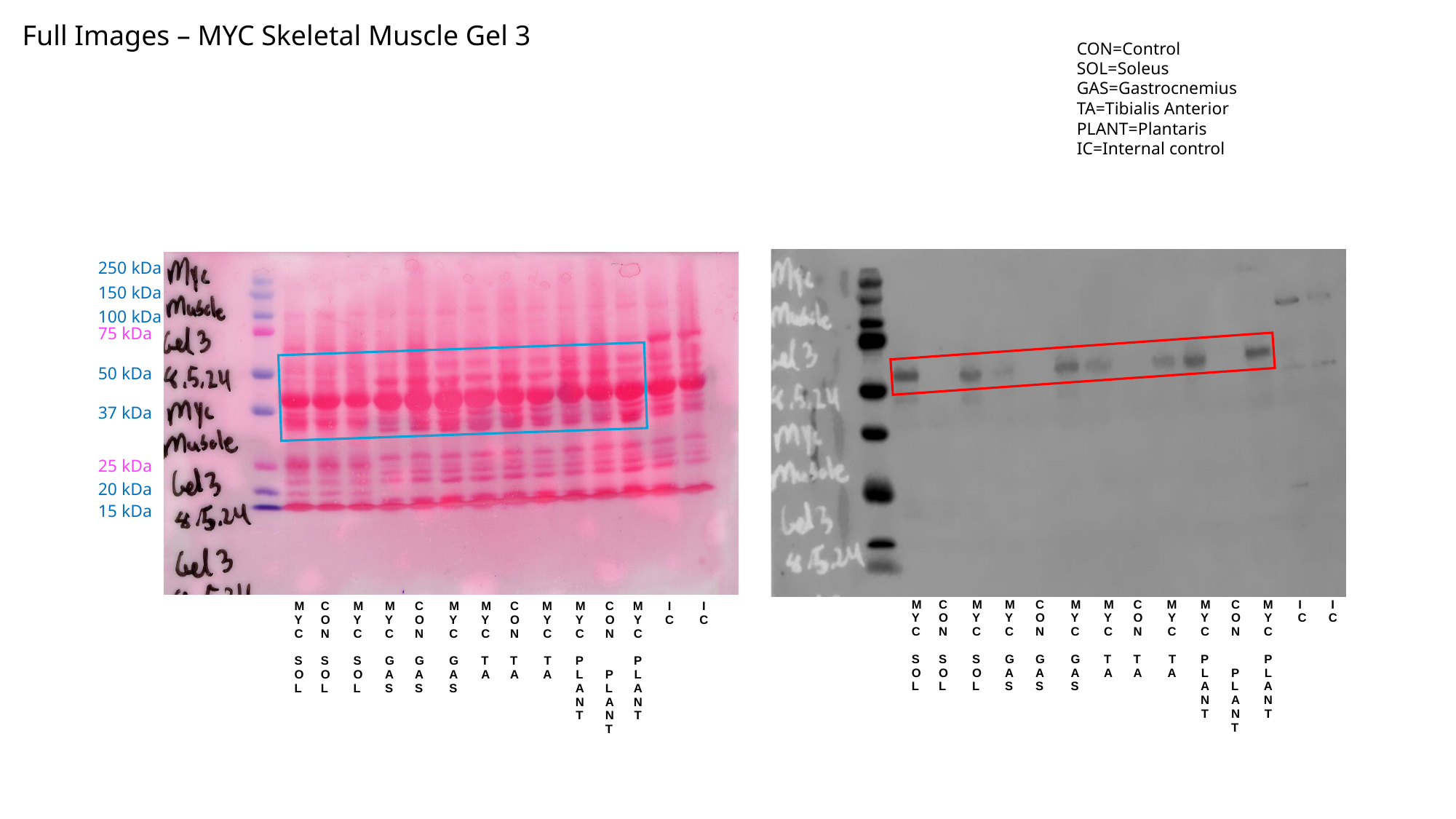

Full Images – MYC Skeletal Muscle Gel 3
CON=Control
SOL=Soleus
GAS=Gastrocnemius
TA=Tibialis Anterior
PLANT=Plantaris
IC=Internal control
250 kDa
150 kDa
100 kDa
75 kDa
50 kDa
37 kDa
25 kDa
20 kDa
15 kDa
| MYC SOL | CON SOL | MYC SOL | MYC GAS | CON GAS | MYC GAS | MYC TA | C O N T A | MYC TA | MYC PLANT | CON PLANT | MYC PLANT | IC | I C |
| --- | --- | --- | --- | --- | --- | --- | --- | --- | --- | --- | --- | --- | --- |
| MYC SOL | CON SOL | MYC SOL | MYC GAS | CON GAS | MYC GAS | MYC TA | C O N T A | MYC TA | MYC PLANT | CON PLANT | MYC PLANT | IC | I C |
| --- | --- | --- | --- | --- | --- | --- | --- | --- | --- | --- | --- | --- | --- |
