# Supplementary figures and images for: The 24-hour molecular landscape after exercise in humans reveals MYC is sufficient for muscle growth
Source: EMBO Rep. 2024 Oct 31;25(12):5810–37. doi: 10.1038/s44319-024-00299-z (PMC11624283; doi:10.1038/s44319-024-00299-z)

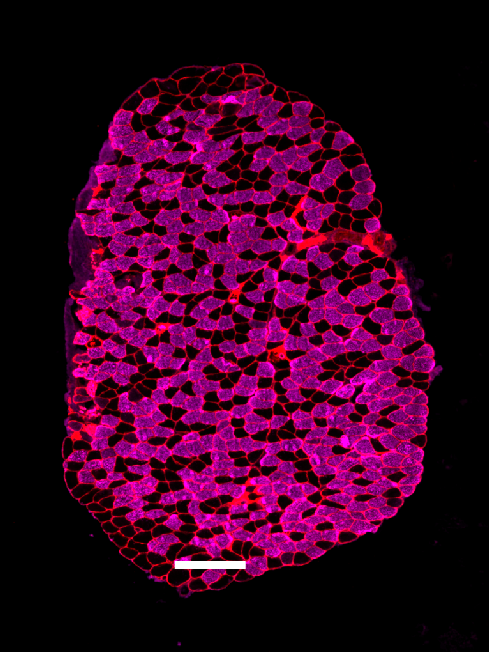

Supplement: Supplementary file 11 — Source data Fig. 6 [file 44319_2024_299_MOESM11_ESM.zip › Figure 6/6G/HSA-Control.tif]

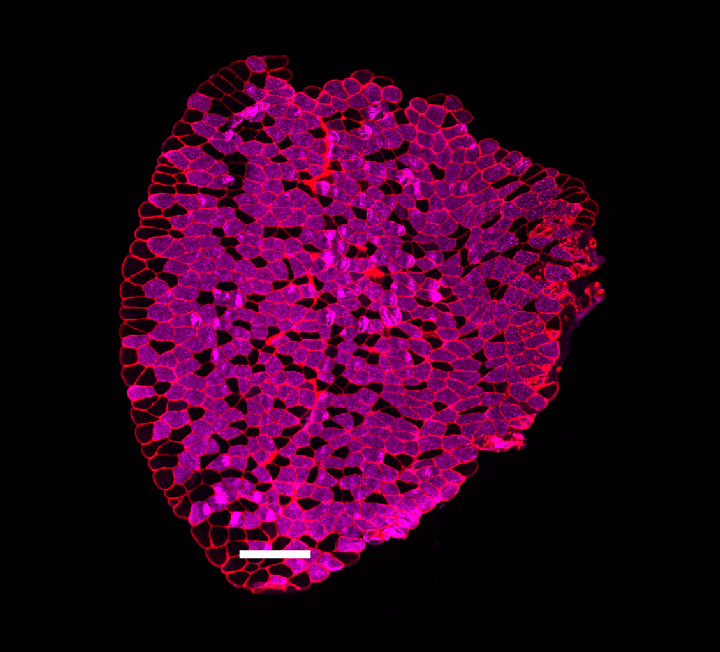

Supplement: Supplementary file 11 — Source data Fig. 6 [file 44319_2024_299_MOESM11_ESM.zip › Figure 6/6G/HSA-MYC.tif]
