# Supplementary material for: The 24-hour molecular landscape after exercise in humans reveals MYC is sufficient for muscle growth
Source: EMBO Rep. 2024 Oct 31;25(12):5810–37. doi: 10.1038/s44319-024-00299-z (PMC11624283; doi:10.1038/s44319-024-00299-z)
Supplement: Supplementary file 12 — Appendix and EV Figure Source Data [file 44319_2024_299_MOESM12_ESM.zip › Appendix Figure S1/Liver image.pptx]

## Slide 1
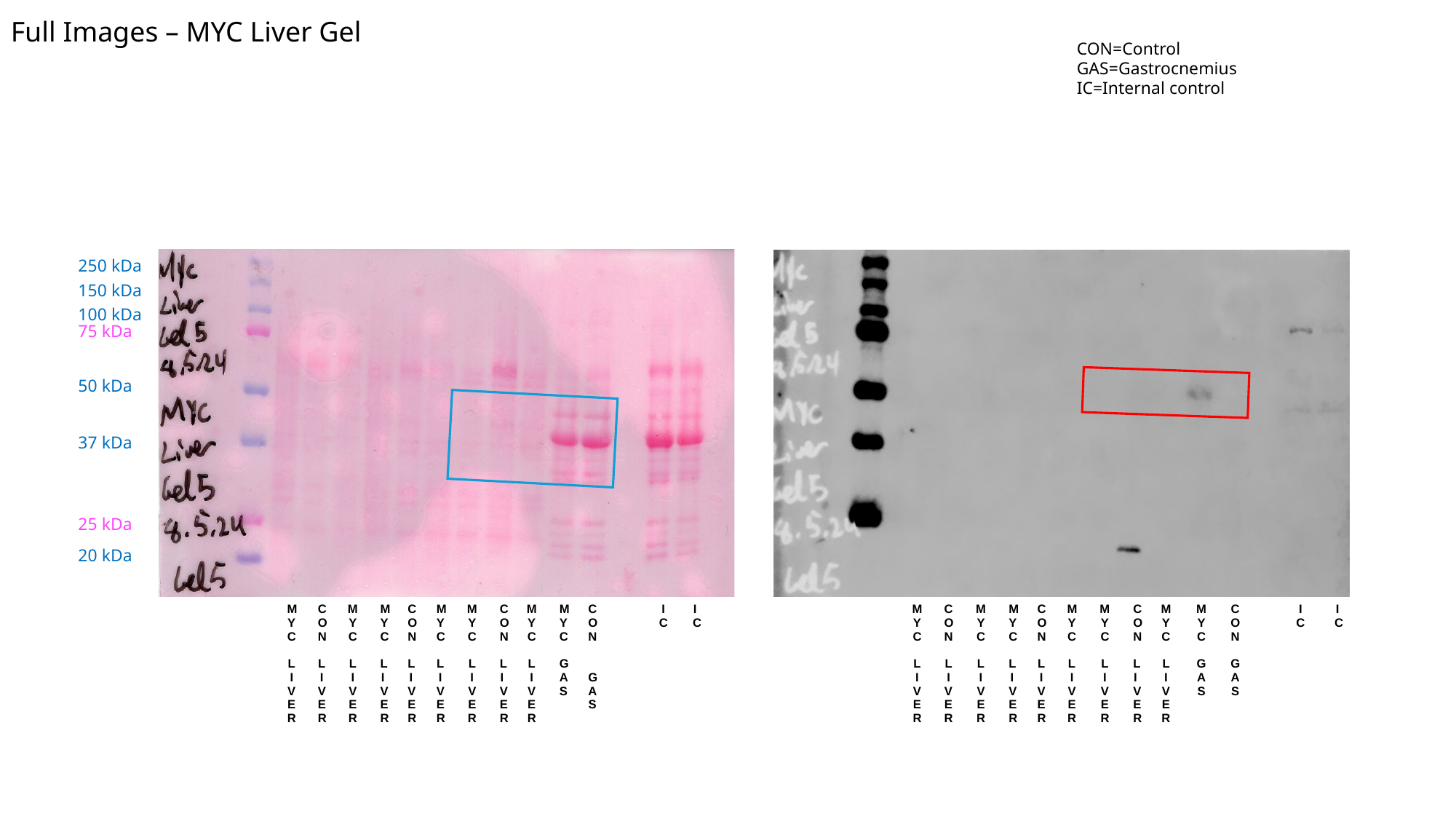

Full Images – MYC Liver Gel
CON=Control
GAS=Gastrocnemius
IC=Internal control
250 kDa
150 kDa
100 kDa
75 kDa
50 kDa
37 kDa
25 kDa
20 kDa
| MYC L IVER | CON L IVER | MYC L I VER | MYC L IVER | CON L IVER | MYC L IVER | MYC L I VER | C O N L IVER | MYC L I VER | MYC GAS | CON GAS | | I C | IC |
| --- | --- | --- | --- | --- | --- | --- | --- | --- | --- | --- | --- | --- | --- |
| MYC L IVER | CON L IVER | MYC L I VER | MYC L IVER | CON L IVER | MYC L IVER | MYC L I VER | C O N L IVER | MYC L I VER | MYC GAS | CON GA S | | I C | IC |
| --- | --- | --- | --- | --- | --- | --- | --- | --- | --- | --- | --- | --- | --- |
