# Supplementary material for: The 24-hour molecular landscape after exercise in humans reveals MYC is sufficient for muscle growth
Source: EMBO Rep. 2024 Oct 31;25(12):5810–37. doi: 10.1038/s44319-024-00299-z (PMC11624283; doi:10.1038/s44319-024-00299-z)
Supplement: Supplementary file 13 — Expanded View Figures [file 44319_2024_299_MOESM13_ESM.pdf]

Expanded View Figures

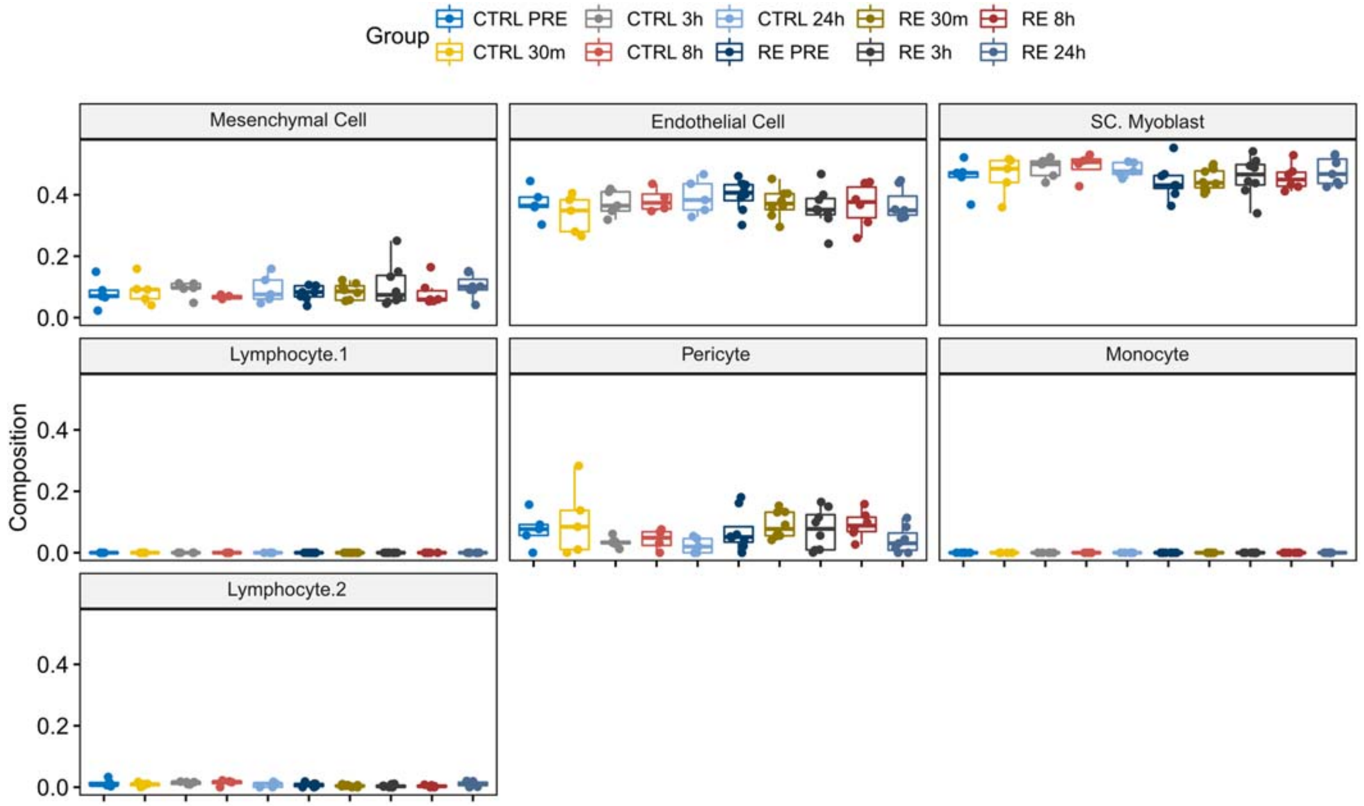

Figure EV1. Cell composition of muscle biopsies through data deconvolution.

Cell composition of all skeletal muscle biopsy time points excluding "myocytes" (CTRL $n = 5$, RE $n = 8$). The box represents the 25th–75th percentile, the line represents the median, and the whiskers represent Min to Max, excluding outliers. SC satellite cell, CTRL control group, RE resistance exercise group.

 © The Author(s)

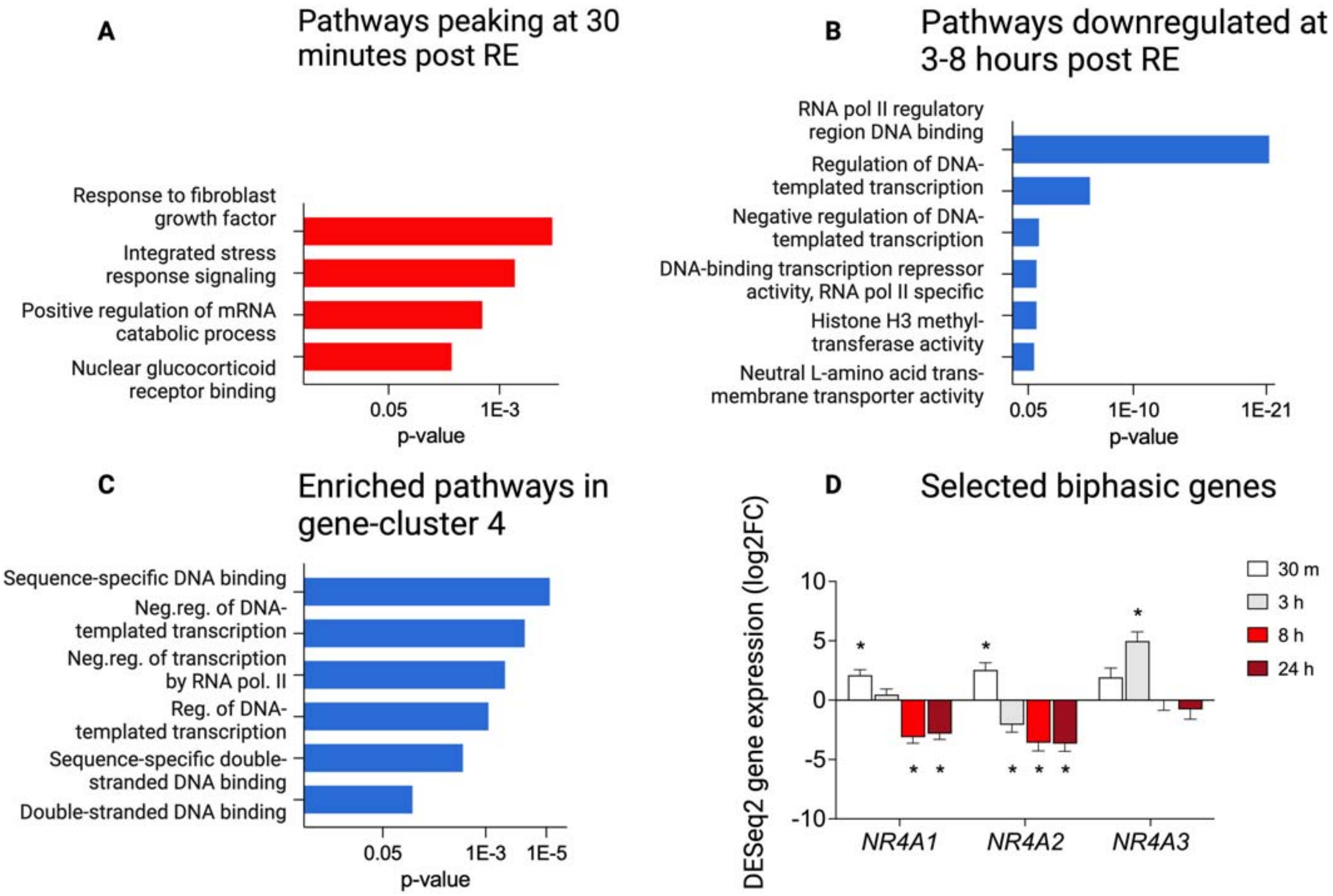

Figure EV2. Targeted gene set enrichment analysis.

Pooled gene ontology (GO) biological processes and molecular function gene sets. (**A**) Targeted analysis of gene sets peaking at 30 min post resistance exercise (RE). (**B**) Targeted analysis of gene sets significantly enriched in genes downregulated 3–8 h post RE. (**C**) Targeted analysis of biphasic DEGs composing cluster 4, as presented in Fig. 2D. *NR4A1* adj. $p = 0.0049$ at 30 min, adj. $p = 6.5E{-}8$ at 8 h, adj. $p = 1.0E{-}6$ at 24 h, *NR4A2* adj. $p = 0.0135$ at 30 min, adj. $p = 0.0081$ at 3 h, adj. $p = 4.7E{-}6$ at 8 h, adj. $p = 2.4E{-}6$ at 24 h, *NR4A3* adj. $p = 1.8E{-}8$ at 8 h. (**A–C**) Gene ontology (GO) gene set enrichment analysis is analyzed using a Fisher exact test with Benjamini–Hochberg *p*-value correction. (**D**) Gene expression of selected genes with a biphasic gene expression pattern, up early/down late, $n = 8$. DESeq2 was calculated using a Wald test with a Benjamini–Hochberg *p*-value correction. *$p < 0.05$ vs Pre values. Neg. negative, Reg. regulation.

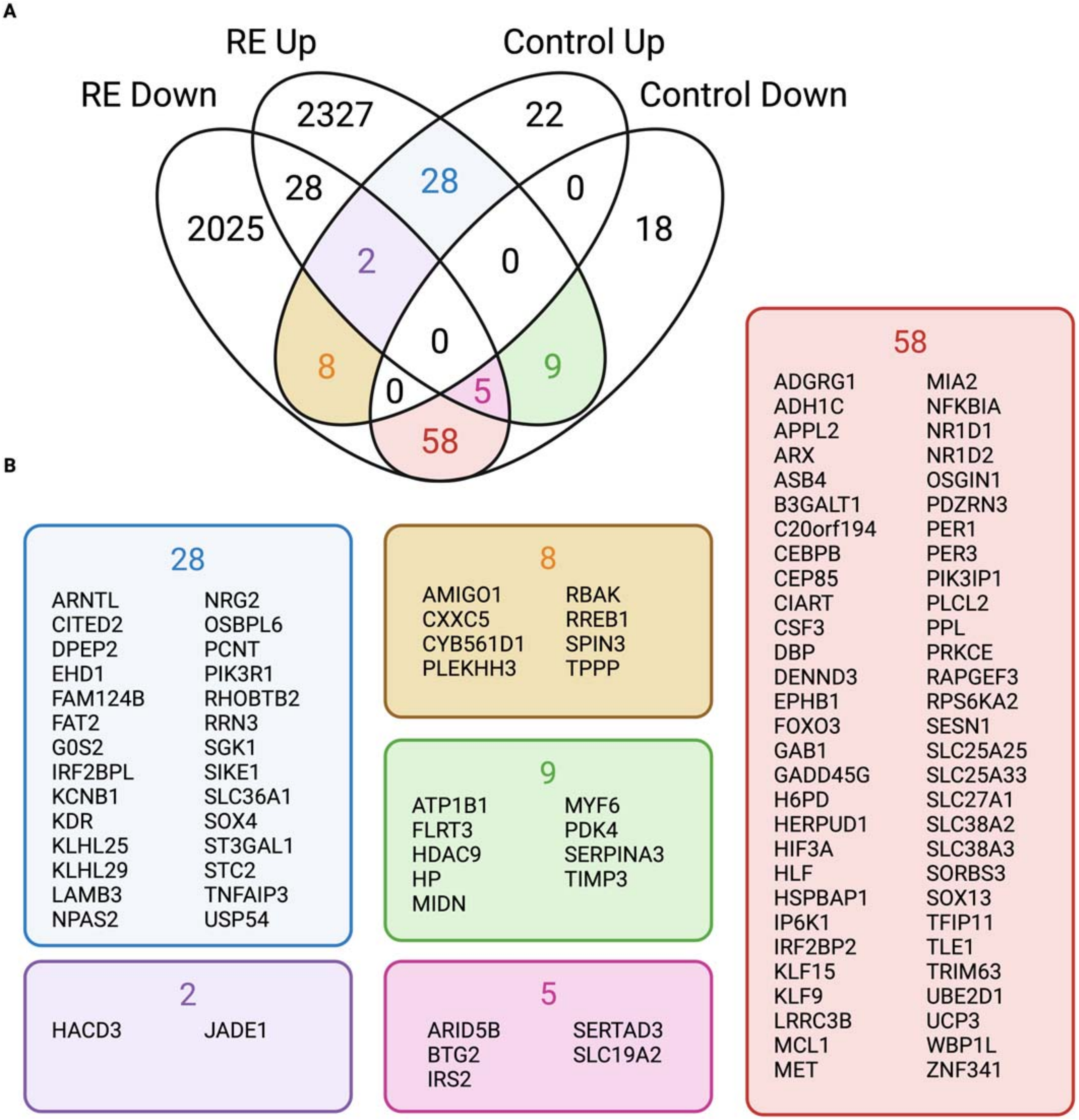

Figure EV3. Venn diagram illustrating overlap in gene expression between the biopsy-only control group and the post-RE response at 3 and 8 h.

(**A**) Venn-diagram of up and downregulated differentially expressed gene lists from the resistance exercise and control group. RE resistance exercise. (**B**) Genes corresponding to the overlap presented in (**A**).

 © The Author(s)

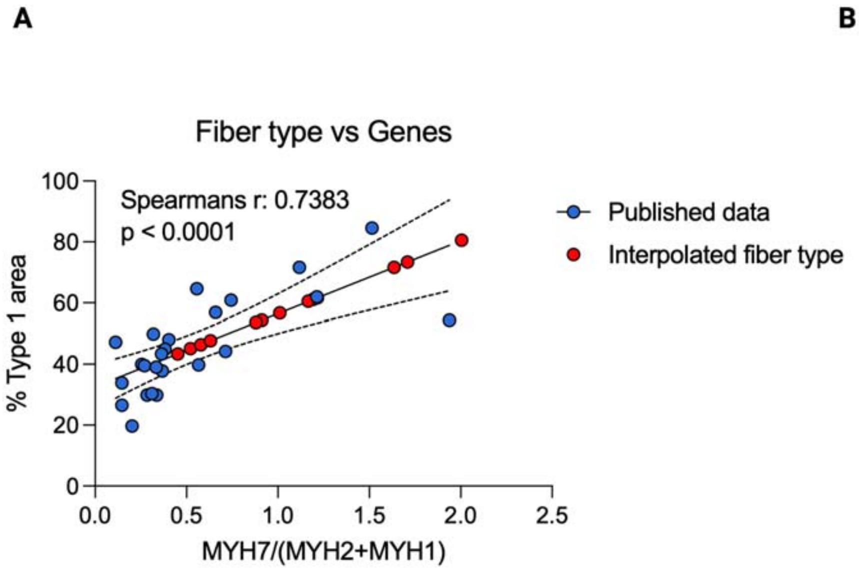

B

Interpolated fiber type area distribution

Participant	Type I area %
Con 1	45.1
Con 2	43.4
Con 3	61.6
Con 4	61.1
Con 5	60.5
Avg.	54.3 ± 9.2
RE 1	46.4
RE 2	56.7
RE 3	54.4
RE 4	71.6
RE 5	53.6
RE 6	80.5
RE 7	47.7
RE 8	73.4
Avg.	60.5 ± 12.2

Figure EV4. Interpolated fiber type area distribution of muscle samples.

(**A**) Correlation of gene expression data with type I fiber area asses by muscle histology ($p = 3.0E{-5}$), data from Reitzner et al (2024). Blue dots = Data points from Reitzner et al (2024), Red dots = interpolated values based on gene data. (**B**) Data table of type I fiber area % in each participant.

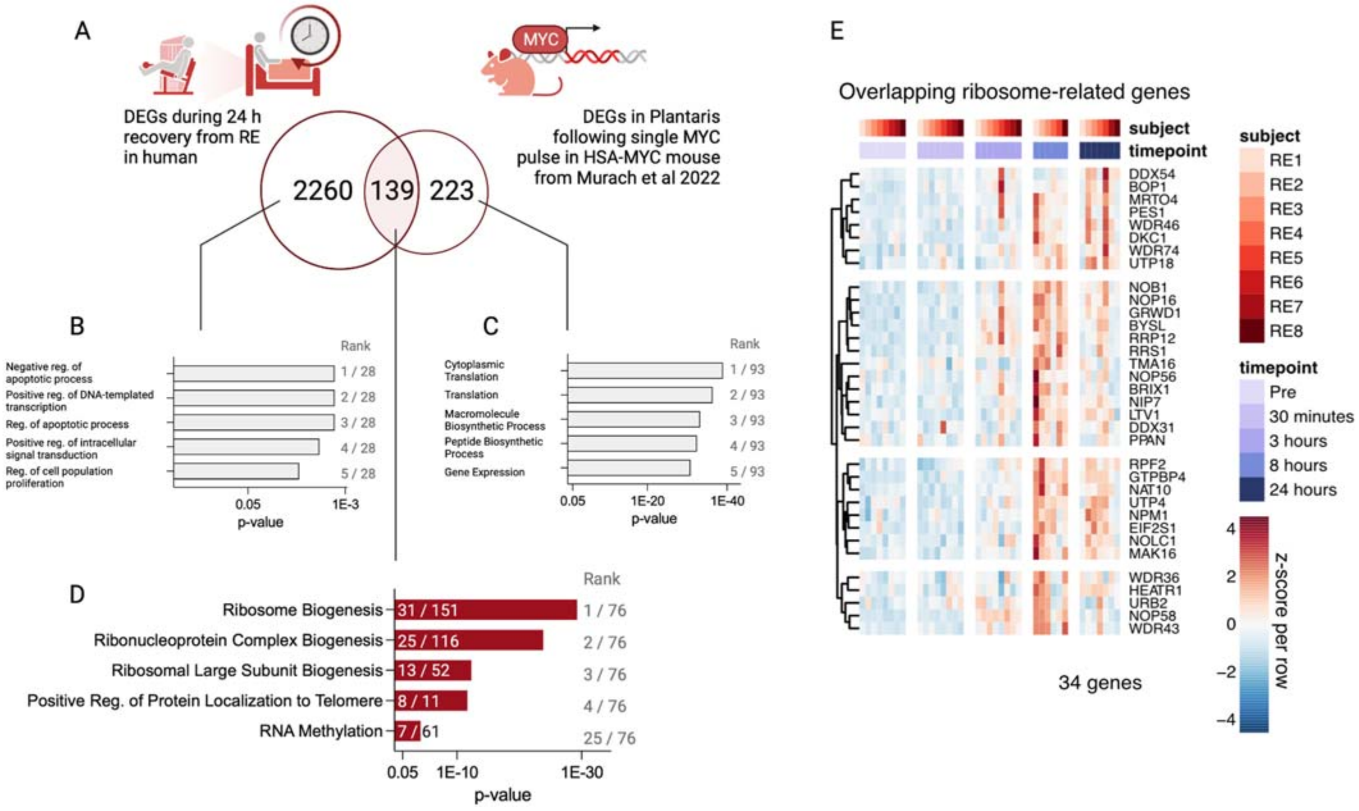

Figure EV5. Transcriptional similarities between human RE recovery and MYC overexpression in mouse plantaris muscle.

(**A**) Comparison of upregulated DEGs across 24 h of RE recovery in humans ($n = 8$) vs plantaris muscle from MYC-overexpressing mice from Murach et al (2022). (**B–D**) Top gene sets (GO: Biological processes) based on DEGs in (**B**) the human exclusive gene list, (**C**) MYC mouse exclusive gene list, and (**D**) overlapping gene list, respectively. (**B–D**) Gene ontology (GO) gene set enrichment analysis is analyzed using a Fisher exact test with Benjamini–Hochberg *p*-value correction. Gene sets are ranked according to their adj. *p*-values. (**G**) Heatmap showing DEG pattern for ribosome-related genes overlapping human RE response to a MYC response in mouse plantaris muscle. Source data are available online for this figure.

 © The Author(s)
